# Supplementary material for: National survey to estimate sodium and potassium intake and knowledge attitudes and behaviours towards salt consumption of adults in the Sultanate of Oman
Source: BMJ Open. 2020 Oct 23;10(10):e037012. doi: 10.1136/bmjopen-2020-037012 (PMC7590363; doi:10.1136/bmjopen-2020-037012)
Supplement: Supplementary data [file bmjopen-2020-037012supp001.pdf]

# Supplementary Material

# Text S1. Material and Methods

## 1.1 Study Design

A national cross-sectional population-based survey was conducted using a representative sample of the Sultanate of Oman based on the WHO Stepwise approach to Surveillance (STEPS) of NCD risk factors (1), comprising the following aspects:

- **Step 1:** This consisted of face-to-face interviews using advanced standardized nation-specific version of the STEPS questionnaire and locally-adapted show cards to facilitate the understanding and operationalization of some questions. Socio-demographic characteristics, key behavioural risk factors, lifestyles, eye and ear health, history of chronic diseases and health care coverage were also elicited to better define exposure and health care seeking and control.
- **Step 2:** This involved physical measurements (e.g. weight, height, waist, hip circumference), and determination of blood pressure, heart rate, and vision function to investigate biological risk factors such as hypertension, overweight, obesity, and vision issues.
- **Step 3:** This aimed at determinations of biochemical markers levels (e.g. fasting capillary blood for glucose and lipid profile, and non-fasting urinary samples for sodium and creatinine) to identify hypercholesterolemia, hyperglycaemia, and sodium intake.

## 1.2 Sample Design

**2.1** **Target Population:** The population of interest was all non-institutionalized persons, which included all men and women 18 years of age or older (Omani & non-Omani) who reside in the country.

**2.2** **Inclusion population:** The target population includes all persons who consider the country to be their usual place of residence. This definition comprises those individuals residing in the country even though they may not be considered a citizen of the country.

**2.3** **Exclusion population:** Those household members who were younger than 18 years of age; persons who have cognitive impairment that hampered understanding the questions to provide clear feedback; visitors (tourists) to the country; and, institutionalized people or those who indicated their usual place of residence was a military base, labour camps or group quarters, were excluded from the survey.

## 1.3 Sample Frame

For the 2010 National census, the whole of the Sultanate of Oman was divided into 15,077 census blocks. A census block was defined as a collection of units (residential units), which



Al-Mawali A, *et al. BMJ Open* 2020; 10:e037012. doi: 10.1136/bmjopen-2020-037012

includes 60 units or less, and it could involve one or more enumeration areas. The census block is the smallest unit of field work for census data collectors. A total of 399,274 households[1] were identified in these blocks (260,120 Omani and 139,154 non-Omani). Households for Omanis and non-Omanis could not be differentiated unless physically visited as they lived closely together and there were no specific places (blocks) for non-Omani. The number of blocks and households in each governorate was provided in the table below according to the National Census 2010 and the map below shows the distribution of blocks over the Sultanate of Oman by governorates (National Centre for Statistics & Information (NCSI), Sultanate of Oman). The same census blocks were updated and used in this NCD Risk Factor Survey as shown in Table 1.

**Table 1: Number of blocks and households by governorate based on 2010 National Census comprising the sampling frame for Oman NCD risk factors survey, 2017**

| Governorate | Number of blocks | Number of Omani households |
|---|---|---|
| **Muscat** | 3514 | 62299 |
| **Dhofar** | 1768 | 17926 |
| **Ad Dakhiliyah** | 1712 | 34611 |
| **North Sharqiyah** | 1173 | 20044 |
| **South Sharqiyah** | 1097 | 22455 |
| **North Batinah** | 2248 | 47193 |
| **South Batinah** | 1335 | 29355 |
| **Al-Dhahirah** | 884 | 14386 |
| **Al-Buraimi** | 531 | 5939 |
| **Musandam** | 513 | 3216 |
| **Al-Wusta** | 302 | 2696 |
| **Total** | **15077** | **260120** |

### 1.3.1 Mapping and listing update

Two persons were appointed and trained to perform the mapping and listing operation for each governorate by using 2010 census maps from the National Centre for Statistics and Information. Personnel visited selected blocks to update maps that depicted all the households within the selected block and update the list of households within those blocks, including

[1] A "Household (HH)" was defined as "either a one-person household, defined as an arrangement in which one person makes provision for his or her own food or other essentials for living without combining with any other person to form part of multi-person household or a multi-person household, defined as a group of two or more persons both related and unrelated living together who make common provision for food or other essentials for living".



Al-Mawali A, *et al. BMJ Open* 2020; 10:e037012. doi: 10.1136/bmjopen-2020-037012

additional information (e.g. house number - Name of household head – nationality – language – description if needed).

## 1.4 Sampling Design

A multi-stage stratified cluster sampling was designed to select 9053 eligible subjects. Stratification was made on two factors: governorate and nationality (Omani and Non-Omani). The sample was drawn from the 2010 census block area (clusters). An ***equal size sample (cluster)*** was systematically randomly selected from each governorate. Selected clusters (blocks) had to be updated before the households were selected for survey (see previous section).

### 1.4.1 Rationale for preferring equal sample selection rather than proportional to population size

1. To get the desired precision and estimates of overall results and figures at the governorate level.
2. Proportional allocation would have resulted in a very small sample in a small governorate e.g. Al-Wusta with low precision in the estimate.
3. Equal sampling allowed high precision in individual stratum.
4. The experience from previous surveys in Oman. The lowest response rate was in Muscat governorate and one third of population live in Muscat so if proportional selection was used this would have directly affected the overall response rate.

In the first stage, all governorates in the Sultanate of Oman (11 governorates) were selected.

In the second stage, we stratify blocks (PSU) based on geographical location, 550 clusters, from all over the Sultanate of Oman (50 from each governorate) using systematic random sampling (Secondary Sampling Units (SSU)).

In the third stage: [Tertiary Sampling Unit (TSU)], the following was considered:

i) The households in each cluster were listed within each governorate i.e. households from the first cluster, then households from the second cluster till households from the 50th cluster.

ii) The households in all clusters were aggregated into two lists by nationality (Omani and Non-Omani households) in each governorate

iii) 823 households were systematically and randomly selected from list of all households according to above in each governorate, from these two lists (Omani and Non-Omani household lists) according to the ratio of Omani and Non-Omani households in each governorate.

In the fourth stage: Ultimate sampling unit USU), One eligible individual from each household aged 18 years old or older was selected randomly by a program on the Android tablet.

### 1.4.2 Sample Size

A WHO STEPS standard formula was used in the calculation of the sample size based on the guidelines/recommendations of the STEPS survey.

n= $Z_2$ * P (1-P)/$d_2$

Where;

n= the required sample size

Z= the probability value associated with the confidence level

P= the prevalence rate of NCDs risk factors in the country

d= the desired margin of error (precision).

In turn:

Z= 1.96 (95% confidence interval as recommended)

P= 0.5 (the conservative value of prevalence rate)

e= 0.05 (as recommended in the guidelines)

Using these values, the initial calculation was: n= 384 households

Also taken into account for sample size calculations were: a value of design effect, as recommended in STEPS surveys, to be 1.5; and, an anticipated response rate of 70% was estimated. By adjusting the sample by these factors, the sample size per cluster (governorate) results in:

$$n = \frac{384\ x\ 1.5}{0.7} = 823 \text{ households}$$

To get the desired precision and overall figures adequate for age-sex groups and for overall estimate on governorates level, the sample size was:

n= 823 x 11 = 9053 households, with one individual selected per household

The 9053 households were distributed equally by governorate (823), proportional to nationality, according to the ratio of Omani and non-Omani households in each governorate as in Table 2.

**Table 2: Oman STEPS survey sample size distributed, by governorate and nationality, 2017**

| Governorate | Selected no. of blocks in each governorate | Omani | Non-Omani | Total Households |
|---|---|---|---|---|

| | | | | |
|---|---|---|---|---|
| **Muscat** | 50 | 318 | 505 | **823** |
| **Dhofar** | 50 | 495 | 328 | **823** |
| **Ad Dakhiliyah** | 50 | 763 | 60 | **823** |
| **North Sharqiyah** | 50 | 520 | 303 | **823** |
| **South Sharqiyah** | 50 | 667 | 156 | **823** |
| **North Batinah** | 50 | 677 | 146 | **823** |
| **South Batinah** | 50 | 699 | 124 | **823** |
| **Al-Dhahirah** | 50 | 586 | 237 | **823** |
| **Al-Buraimi** | 50 | 722 | 101 | **823** |
| **Musandam** | 50 | 713 | 110 | **823** |
| **Al-Wusta** | 50 | 663 | 160 | **823** |
| **Total** | **550** | **6823** | **2230** | **9053** |

## 1.5 Data collection instruments and procedures used in the survey

### 1.5.1 Selection of participants

Upon selection of a household, all potential individuals for interview were identified and their age and gender recorded in a household list, subsequently used to determine selection probabilities and response rate. To randomly select an eligible individual from a household, the Kish method was used using an electronic tablet and software. Once individuals were selected, they were informed about the survey aims and asked to provide their consent to participate in the interview and subsequent measurement procedures.

### 1.5.2 Questionnaire (STEP 1)

An advanced standardized[2] country-specific version of the questionnaire, based on the Core and Expanded version of the WHO STEPS Instrument for Chronic Disease Risk Factor Surveillance[3] and country-specific requirements, contained 11 Core, 1 Optional and 4 country-specific modules, to determine socio-demographic characteristics of participants, key behavioural risk factors (tobacco use, harmful alcohol consumption, diet with low fruits and vegetable intake, high salt intake, physical inactivity), given lifestyle advises, eye and ear health profile, history of chronic diseases, and health care coverage for diabetes, hypertension and dyslipidaemia, as well as cervical and breast screening.

In order to enhance the comparability with other countries in Arab region, the questionnaires from the Kuwait (2014), the Qatar (2012), the Bahrain (2007) and the Saudi Arabia (2005) STEPS surveys were taken into consideration. In addition, the questionnaire was translated from the original English version into Arabic as well as back translated, adapted to the local environment and needs, and piloted on 10 eligible respondents in terms of wording and

[2] validated
[3] Reference: The WHO STEPS Instrument for Chronic Disease Risk Factor Surveillance (1)

understanding. The data collection was conducted in two languages, namely: Arabic and English.

**Table 3: Questionnaire content by STEPS survey and county-specific modules and questions in the Oman survey, by type, 2017**

| | STEP and county-specific modules content | Questions | | |
|---|---|---|---|---|
| **I** | **Core modules (11)** | **Core** | **Expanded** | **Country-specific** |
| 1 | Demographic information | 4 | 5 | 3 |
| 2 | Tobacco use | 11 | 7 | |
| 3 | Alcohol consumption | 12 | | |
| 4 | Diet, including dietary salt | 8 | 3 | 2 |
| 5 | Physical activity | 15 | 1 | |
| 6 | History or raised blood pressure | 5 | | 1 |
| 7 | History of diabetes | 6 | | 1 |
| 8 | History of raised total cholesterol | 5 | | 1 |
| 9 | History of cardiovascular disease | 3 | | |
| 10 | Lifestyle advise | 1 | | |
| 11 | Cancers (cervical cancer screening) | 1 | | 3 |
| | **Subtotal 1** | 71 | 16 | 11 |
| **II** | **Optional Module (1)** | | | |
| 1 | Tobacco policy | 7 | | 8 |
| | **Subtotal 2** | 7 | | 8 |
| **III** | **Country-specific Modules (4)** | | | |
| 1 | Family history of chronic disease | | | 8 |
| 2 | Asthma | | | 11 |
| 3 | Eye health | | | 3 |
| 4 | Ear health | | | 4 |
| | **Subtotal 3** | | | 26 |
| **Grand Total: 139** | | **78** | **16** | **45** |

## 1.5.2.1 Demographic information

All eligible household members aged 18 and above were listed with one eligible member selected randomly to answer the demographic information questionnaire. Demographic information was assessed in terms of age, sex, marital status, educational status, employment status, and family income.

## 1.5.2.2 Tobacco use

Tobacco use was assessed in terms of current smoking (past 30 days), whether daily or non-daily, and former smoking or never smoking status, age of initiation and duration of smoking, type and quantity of tobacco use daily or weekly, smokeless tobacco use type and frequency, and exposure to second-hand smoke at home or workplace. Smoking cessation attempts, having

received health professionals' advice, age and time since stopping to smoke, recognizing tobacco advertisement, promotion or sponsorship, all while applying 33 questions[4] were also asked. To facilitate recognition of types of tobacco use, data collectors used show cards, depicting types of commonly used tobacco products. Likewise, the above metrics were determined for users of smokeless tobacco products.

### 1.5.2.3 Alcohol consumption

Alcohol consumption was assessed using the concept of a standard drink. A standard drink is any drink containing about 10 g of pure alcohol. Accordingly, determinations have been made from different types of alcoholic beverages consumed, as follows: e.g. 30 ml of spirits, 120 ml of wine or 285 ml of beer. Again, data collectors used show cards depicting types of containers commonly used to consume alcoholic beverages as standard drinks, to determine consumption over 30 and 7 days prior to interview. Also, in an attempt to quantify and estimate total alcohol consumption, interviewers considered not only the most frequent but all types of alcohol consumed (e.g. wine, beer and spirits) and the amount of drinks on such occasions. Also included in the questionnaire were aspects about stopping alcohol consumption for health reasons or impacts.

Respondents who reported consuming alcohol within the past 30 days were classified as current drinkers, while those who identified absence of alcoholic beverages within previous 12 months as abstainers or ex-drinkers. Three risk categories were used to classify respondents who consumed alcohol according to the average amount of alcohol consumed per occasion. Furthermore, heavy ("binge") drinking patterns were determined according to largest number of drinks per drinking occasion and the percentage of those having consumed six or more standard drinks on one occasion during the past 7 days.

### 1.5.2.4 Diet

To assess the dietary patterns of the surveyed population, the respondents were asked about frequency of fruit and vegetable consumption, mean number of portions of these foods consumed daily and weekly, type of oils and fat used for meal preparation, number of meals eaten outside the household per week and the amount of salt added, and/or salty sauces used or processed food or consumed daily, using 13 questions.

Sufficient consumption of fruit and vegetables was assessed in terms of the number of servings and also compared to WHO recommended number of ≥ 5 servings/day and ≥ 5 day/week, with

[4] Based on STEPS and GATS questionnaires (2)

a serving being equal to 80 g. Show cards were used to facilitate the collection data on fruit and vegetable consumption on a typical day. Oil and fat intake show cards were also shown to assess about the type of oil or fat most frequently used for preparing food or cooking.

In turn, salt consumption was assessed by asking about frequency of addition of salt or a salty sauce to food during preparation, or before or while eating; and/or frequency of consumption of processed food high in salt. Participants were also asked about their perception of the quantity of salt they consumed and its link with health problems, as well as about the importance of reducing salt intake, and the measures undertaken to control it. WHO recommends a reduction to <2 g/day sodium (5 g/day salt) in adults (3).[5]

Population mean number of daily portions and of days per week consuming fruits and/or vegetables were calculated. Also, the percentage distribution of respondents according to their servings consumed per day and those meeting the WHO recommendation of fruits and vegetables intake/day were determined.

Regarding salt intake, the proportions of people reporting how often they added salt to foods before eating or when preparing foods, as well as those who think they eat too much salt were determined. Percentage of participants were further determined according to their belief on the importance of salt in diet and whether they thought their consuming to much salt can cause serious problems.

#### 1.5.2.5 Physical activity

Physical activity was assessed based on frequency, duration and intensity of physical activity at three segments: work (paid/ unpaid in and outside home), during transportation and on leisure time, for at least 10 minutes or more continuously per day, using a set of 16 questions[6]. Show cards were used to depict different types and places of physical activity.

According to WHO global recommendations on physical activity for (good) health, throughout a normal week adult should do at least the following amount of exercise (including activity for work, as well as during transport and leisure time): 150 minutes of moderate-intensity physical activity; or 75 minutes of vigorous-intensity physical activity; or an equivalent combination of moderate- and vigorous-intensity physical activity. Mean and median minutes of physical activity per day according to place were computed; as a complement, time spent on sedentary activities on average per day was also calculated. The proportion of respondents not meeting

[5] http://www.who.int/nutrition/publications/guidelines/sodium_intake_printversion.pdf
[6] Based on WHO Global Physical Activity Questionnaire (version 2) (4)



Al-Mawali A, *et al. BMJ Open* 2020; 10:e037012. doi: 10.1136/bmjopen-2020-037012

the WHO recommendations was also calculated. Likewise, the proportion of participants according to levels of physical activity as recommended by WHO were determined.

### 1.5.3 Physical measurements (STEP 2)

#### 1.5.3.1 Blood pressure

Resting blood pressure levels, both systolic (SBP) and diastolic (DBP), were measured using Omron M3 digital blood pressure device as recommended by WHO. The measurements were repeated three times and the three readings were recorded. In order to obtain the measurements under relaxed conditions, persons were asked to void their bladder if needed, resting for 10-15 minutes after the interview, and not having drunk coffee before or during the measurement. In preparation for measurements, participants were asked to sit straight without crossing their legs. Blood pressure was measured placing a universal cuff on the left arm, which was placed with the palm face upward on a table surface at the level of the heart. While taking the readings participants were asked to remain silent. Repeat measurements were taken at 3-minute interval.

Participants were classified according to their blood pressure readings in the following categories: *normal* if their SBP and DBP readings were <140 mm Hg and <90 mmHg, respectively, and *high* if their SBP was ≥ 140 mm Hg and/or the DBP was ≥ 90 mm Hg, or if their readings were normal but they were under treatment for raised blood pressure in the past two weeks. In addition, *high risk* levels of SBP ≥ 160 mm Hg and/or DBP ≥ 100 mm Hg were also determined among participants to assess a higher probability or risk of hypertensive disorder.

Survey participants were also asked whether they were under medication for high blood pressure during the previous two weeks, as prescribed by a physician or other health professional. Respondents with treated and/or controlled raised blood pressure among those with raised blood pressure (SBP ≥140 and/or DBP ≥ 90 mmHg) or currently taking medication for raised blood pressure were further categorized to determine treatment success, treatment failure or being undetected and untreated, as follows:

- Under medication and controlled (treatment success) = those taking medication and having SBP <140 mmHg and DBP <90 mmHg;
- Under medication and uncontrolled (treatment failure) = those taking medication and having SBP ≥140 mmHg and/or DBP ≥90 mmHg;
- Undetected and uncontrolled (health system failure) = those not taking medication and having SBP ≥140 mmHg and/or DBP ≥90 mmHg**.**



Al-Mawali A, *et al. BMJ Open* 2020; 10:e037012. doi: 10.1136/bmjopen-2020-037012

#### 1.5.3.2 Body mass index (BMI)

The height and weight of participants was taken to estimate their body mass index (BMI) as the ratio of weight/height2 (at the nearest decimal kilogram and decimal centimetre, respectively).

Measurements were carried out while standing with the heels together, feet apart, arms at sides and chin parallel to the floor on a flat, horizontal and firm surface (like tile, cement or wooden floor). Standardized and calibrated SECA® 813 digital floor scales and 213 portable stadiometers were used for weight and height measurements, respectively. To measure height and weight more precisely, participants were asked to follow standard procedures, including removal of their shoes and any bulky or heavy clothing to avoid overestimations.

Once BMI ratios determined, sample population was categorized according to the following WHO recommendations: underweight if BMI $< 18.5$, normal weight if BMI was between 18.5 - 24.9, overweight if BMI was between 25.0 - 29.9, and obesity if BMI was $\geq 30$.

Average population BMI levels and proportion distribution among the sample population groups were determined.

#### 1.5.3.3 Waist and hip circumferences

Waist circumference and hip circumference and their ratio were also assessed as other measures of obesity, in particular of central obesity.

Waist and hip circumference measurements were made while a participant remained standing, with feet together and hands on each side of the body, with a non-stretch Seca 201 measuring tape with millimetre precision. Waist circumference was measured by placing a tape measure around the bare abdomen at the midpoint between the lower margin of the last palpable rib and the top of iliac crest (hip bone). Hip circumference was measured by placing a tape measure around the maximum circumference over the buttocks.

The WHO cut-off points of waist circumference that determine waist obesity and categorized risk of metabolic complications and CVD are different for men and women. The waist–hip ratio (WHR) was computed among all respondents, excluding pregnant women.

## 1.6 Data collection

### 1.6.1 Staff recruitment

Interviewers and field supervisors in each governorate were recruited from among staff working in the Ministry of Health.

Overall, 66 data collectors and 11 field supervisors were recruited to participate in the training of data collection along with 1 regional coordinator, 1 IT technician and 1 laboratory technician in each governorate. Data collectors were mainly nurses and health educators nominated for data collection and for measurement of height, weight, waist and hip circumference, blood pressure, blood glucose, lipid profile, and vision testing. Urine samples were measured by the laboratory technicians in the health centre laboratories.

### 1.6.2 Data collection procedures

Field operations were carried out in the governorates during a four-month period in 2017, with the survey period chosen appropriately to avoid Ramadan/Eid periods. A media and advocacy action plan was implemented to raise awareness of the population about the survey, including disseminating information through leaflets, posters, press releases, radio and TV broadcasting, community and local Ministry of Health (MoH) staff participation. In addition to this focused publicity campaign, official identity cards to the field staff issued by MoH were of great help to secure sufficient recognition, cooperation and good responses for the interview in most cases. The respective authorities were also requested to provide the necessary assistance and co-operation to the field staff.

The interviews were conducted in all the governorates. The supervisors approached the selected households in each cluster, explained the aim and objectives of the survey, and sought their consent to participate in the survey. After recording the eligible members within a selected household, one participant was randomly selected from eligible members by the Android tablet. Each interview took place in a secure setting with adequate privacy at the household level. Each participant was interviewed at his/her household. As biomedical tests require 12 hours of fasting, appointments were given based on agreement between the interviewers and the respondents. Interviewers also explained the protocol for 24-hour urine collection to the respondent, obtained informed consent and provided the record sheet on which participants note the start and finish times of their 24-hour urine collection and any missed urine collections in the container.

### 1.6.3 Training for supervisors and data collectors

A one-week training program was conducted by the central research team from the Centre of Studies & Research in collaboration with WHO experts. This training program, which was held in Muscat in December 2016, included the survey objectives and field work staff duties, how to fill in the questionnaire in a polite, motivating and persuasive manner, how to understand the content of the questions if needed to clarify to respondents, how to enter the data and navigate



Al-Mawali A, *et al. BMJ Open* 2020; 10:e037012. doi: 10.1136/bmjopen-2020-037012

the Android tablet, how to ask for written consent and organize data collection and protection of confidentiality of the informant (consent, ID barcode labels, patterns of verbal and nonverbal behaviour), how to perform physical measurements and take biomarkers' samples using equipment applying standardized methodology, and role playing and mock interviews.

The field supervisors were given special instructions afterwards on coordinating process. Trainees who failed to show interest in the survey and those who did not attend the training program on a regular basis were not selected for the fieldwork and were replaced by other staff from the same Governorate after training.

### 1.6.4 Pilot Study

The pilot testing of the survey implementation process was implemented for 2 days (using Android tablets) selecting about 100 households which included males and females as well as Omani and non-Omani households. The pilot was also used to train the key survey personnel, test all survey materials prior to full implementation (skip errors, translation errors, awkward wording, and inadequate response categories), check quality of data collected. Lessons learned, logistic issues, and challenges identified were considered which maximized opportunities for improving the quality in the full survey implementation.

## 1.7 Data Management

### 1.7.1 Data collection

Each participant was allocated an identifier code (PID). The PID code consisted of seven digits the first two digits were the device number; the next three digits were the house listing number in the cluster and the last two digits were the person number in the household.

Data from STEP 1 and STEP 2 were submitted electronically online to the server from a household with identification of individual PID, household geographical location, including cluster with individual PID, day and time of completion. This was done either daily or at least once a week. After being analysed, blood and urine samples' results (STEP 3) were uploaded to the server and merged into the unified dataset, following conversion into SPSS and Microsoft Excel format in a single file.

### 1.7.2 Data validation

The central team (at the Centre of Studies & Research) downloaded data daily from server for data cleaning and management over a period of 6 months. Data management included continuously monitoring data collection, uploading and consolidation processes in the field,

validating quality of the data, creating weights, removing inconsistencies, namely "jump" errors/outliers, absence of data, excess data and invalid data. Moreover, to increase reliability of the collected data, verification of data in field was organized among 500 randomly selected households from all governorates. Accuracy of recording categorical and continued variables was checked using range and logic functions. The team also provided advice on software support and reported any problems or interview errors to the data collection field supervisors.

### 1.7.3 Data analysis

Weightage and Adjustment for sampling variation

Survey data analyses have to take into account whether the results are representative of the sample alone (unweighted analysis) or of the entire target population (weighted analysis). Since the primary objective was to be able to determine the estimates for the whole country, a weighted analysis was considered necessary. Weights adjusting for this complex survey design were required to decrease the risk of biases resulting from diverse factors. The sample weight is comprised of the inverse probability of selection. The household weights took into account the selection probability of the clusters within each stratum and the size (the number of households) of the cluster. The sample weight was also adjusted for non-response at the household level. The individual weight assumed that adults in the same cluster were selected by simple random sampling but the calculation scheme did not take into account the household size. This approach could have biased any key indicators, which was strongly associated with the household size. The individual weight was also adjusted for non-response.

Means, medians, proportions, standard errors, and 95% confidence intervals (95% CI) values were calculated to estimate central and dispersion measures and used to assess prevalence differences of NCD risk factors. Statistical procedures for data calculation and analyses were performed through two programs: EpiInfo in collaboration with WHO, and IBM SPSS (Version 20). All the figures and indicators in the tables were calculated using SPSS complex samples analysis. The figures presented as footnote (with an asterism) under each table were calculated after using population proportion weight. To allow for international comparisons of Oman survey results, age- and sex-adjusted overall values were calculated for all indicators using the direct method and the WHO standard population. Values are presented as footnotes on the tables to limit confusion with the national unadjusted data. It should also be noted that the estimates shown for governorates in the tables should be treated with caution as they represent the respondents in the respective governorate, and not the governorate itself.

## 1.8 Ethical considerations

Two informed consent forms, one for filling in the questionnaire and performing physical measurements (e.g. STEP 1 and STEP 2) and another for taking blood and urine samples for biomarkers (STEP 3), were requested to be signed by each participant. To enhance participation, an information letter was sent to all selected households in advance of data collection, identifying purpose, benefits and the voluntary participation in the survey.

To guarantee the high level of confidentiality and data security, every eligible subject was granted a unique identification number which was used for any reference from the register, with the exception of providing a personal feedback to a particular eligible subject for medical reasons.

Prior to its implementation, the survey was approved by the Research and Ethical Review & Approval Committee (RERAC) of the Ministry of Health.



Al-Mawali A, *et al. BMJ Open* 2020; 10:e037012. doi: 10.1136/bmjopen-2020-037012
