# Supplementary material for: National survey to estimate sodium and potassium intake and knowledge attitudes and behaviours towards salt consumption of adults in the Sultanate of Oman
Source: BMJ Open. 2020 Oct 23;10(10):e037012. doi: 10.1136/bmjopen-2020-037012 (PMC7590363; doi:10.1136/bmjopen-2020-037012)
Supplement: Supplementary data [file bmjopen-2020-037012supp002.pdf]

Table S1. Comparison of the general characteristics of the study participants with those of the national sample of the 2017 WHO STEPS Survey carried out in Oman.

	Salt and Potassium Survey mean (SD)			2017 WHO STEPS National Survey † mean (95% CI)		
	**All (n=569)**	**Men (n=193)**	**Women (n=376)**	**All (n=6,582)**	**Men (n=3,365)**	**Women (n=3,217)**
Age (yrs)	39.4 (13.1)	38.7 (14.3)	39.8 (12.5)	38.2 (37.4-39.0)	38.0 (37.1-38.8)	38.6 (37.3-39.8)
Height (cm)	159.4 (11.2)	167.9 (9.7)	154.9 (9.2)	-	167.4 (166.6-168.2)	156.1 (155.5-156.6)
Weight (kg)	74.9 (21.5)	81.4 (22.5)	71.4 (20.1)	-	74.9 (73.6-76.2)	68.1 (66.6-69.7)
BMI (kg/m²)	29.3 (7.2)	28.9 (7.6)	29.5 (7.0)	27.3 (26.9-27.6)	26.7 (26.3-27.2)	27.9 (27.3-28.5)
Waist circ. (cm)	93.8 (15.7)	95.0 (15.7)	93.2 (15.7)	-	90.0 (89.0-91.0)	87.6 (86.1-89.1)
Hip circ. (cm)	104.5 (15.0)	102.6 (13.7)	105.5 (15.6)	-	101.2 (98.8-103.5)	102.5 (99.2-105.7)
Systolic BP (mmHg)	125.9 (18.2)	134.0 (17.0)	121.7 (17.3)	124.9 (124.0-125.7)	130.3 (129.2-131.4)	119.1 (117.7-120.4)
Diastolic BP (mmHg)	80.9 (10.7)	83.4 (11.5)	79.7 (10.1)	79.5 (78.9-80.1)	81.5 (80.6-82.5)	77.4 (76.7-78.1)
Pulse rate (b/min)	79.8 (10.5)	78.5 (11.8)	80.4 (9.8)	79.4 (78.7-80.2)	77.7 (76.4-78.9)	81.3 (80.6-82.0)
Hypertension (%)	30.8	39.9	26.1	27.5	33.1	21.5

†https://www.who.int/ncds/surveillance/steps/Oman_STEPS_2017_Data_Book.pdf?ua=1

Al-Mawali A, *et al. BMJ Open* 2020; 10:e037012. doi: 10.1136/bmjopen-2020-037012
