# Supplementary material for: National survey to estimate sodium and potassium intake and knowledge attitudes and behaviours towards salt consumption of adults in the Sultanate of Oman
Source: BMJ Open. 2020 Oct 23;10(10):e037012. doi: 10.1136/bmjopen-2020-037012 (PMC7590363; doi:10.1136/bmjopen-2020-037012)
Supplement: Supplementary data [file bmjopen-2020-037012supp003.pdf]

Table S2. Characteristics of excluded participants and comparison with those included in the final analysis.

Variable	Included (n=569)	Excluded (n=159)	P value*
Age (years)	39.4 (13.1)	37.5 (13.9)	0.037
Height (cm)	159.4 (11.2)	158.7 (8.6)	>0.05
Weight (kg)	74.9 (21.5)	73.4 (18.3)	>0.05
BMI (kg/m^2)	29.3 (7.2)	29.1 (7.0)	>0.05
Waist circumference (cm)	93.8 (15.7)	92.3 (17.9)	>0.05
Hip circumference (cm)	104.5 (15.0)	102.6 (16.4)	>0.05
Systolic blood pressure (mm Hg)	125.9 (18.2)	124.2 (18.8)	>0.05
Diastolic blood pressure (mm Hg)	80.9 (10.7)	79.1 (11.6)	0.025
Pulse rate (b/min)	79.8 (10.5)	80.9 (10.5)	>0.05
Hypertension (%)	27.4	23.9	

Results are mean (SD), *by Mann-Whitney U-test
Hypertension: SBP/DBP >=140/90 mmHg

Al-Mawali A, *et al. BMJ Open* 2020; 10:e037012. doi: 10.1136/bmjopen-2020-037012
