# Supplementary material for: National survey to estimate sodium and potassium intake and knowledge attitudes and behaviours towards salt consumption of adults in the Sultanate of Oman
Source: BMJ Open. 2020 Oct 23;10(10):e037012. doi: 10.1136/bmjopen-2020-037012 (PMC7590363; doi:10.1136/bmjopen-2020-037012)
Supplement: Author's manuscript [file bmjopen-2020-037012.draft_revisions.pdf]

BMJ Open is committed to open peer review. As part of this commitment we make the peer review history of every article we publish publicly available.

When an article is published we post the peer reviewers' comments and the authors' responses online. We also post the versions of the paper that were used during peer review. These are the versions that the peer review comments apply to.

The versions of the paper that follow are the versions that were submitted during the peer review process. They are not the versions of record or the final published versions. They should not be cited or distributed as the published version of this manuscript.

BMJ Open is an open access journal and the full, final, typeset and author-corrected version of record of the manuscript is available on our site with no access controls, subscription charges or pay-per-view fees (http://bmjopen.bmj.com).

If you have any questions on BMJ Open's open peer review process please email info.bmjopen@bmj.com

BMJ Open

Sodium and potassium intake, and knowledge attitudes and behaviour towards salt consumption: a national survey of adults in the Sultanate of Oman

Journal:	*BMJ Open*
Manuscript ID	bmjopen-2020-037012
Article Type:	Original research
Date Submitted by the Author:	16-Jan-2020

Complete List of Authors:	Al-Mawali, Adhra; Ministry of Health Oman, Centre of Studies & Research<br>D'Elia, L; "Federico II" University of Naples Medical School, Department of Clinical Medicine and Surgery<br>Jayapal, Sathish; Ministry of Health Oman, Centre of Studies & Research<br>Morsi, Magdi; Ministry of Health Oman, Centre of Studies & Research<br>Al-Shekaili, Waleed ; Ministry of Health Oman, Centre of Studies & Research<br>Pinto, Avinash; Ministry of Health Oman, Centre of Studies & Research<br>Al-Kharusi, Hilal ; Ministry of Health Oman, Centre of Studies & Research<br>Al Bulushi, Zainab; Ministry of Health Oman, Centre of Studies & Research<br>Idikula, John; Ministry of Health Oman, Centre of Studies & Research<br>Al Harrasi, Ayaman; Ministry of Health Oman, Centre of Studies & Research<br>Cappuccio, Francesco Paolo; University of Warwick, Warwick Medical School, Division of Health Sciences

Keywords:	EPIDEMIOLOGY, NUTRITION & DIETETICS, PUBLIC HEALTH

SCHOLARONE™
Manuscripts

I, the Submitting Author has the right to grant and does grant on behalf of all authors of the Work (as defined in the below author licence), an exclusive licence and/or a non-exclusive licence for contributions from authors who are: i) UK Crown employees; ii) where BMJ has agreed a CC-BY licence shall apply, and/or iii) in accordance with the terms applicable for US Federal Government officers or employees acting as part of their official duties; on a worldwide, perpetual, irrevocable, royalty-free basis to BMJ Publishing Group Ltd ("BMJ") its licensees and where the relevant Journal is co-owned by BMJ to the co-owners of the Journal, to publish the Work in this journal and any other BMJ products and to exploit all rights, as set out in our licence.

The Submitting Author accepts and understands that any supply made under these terms is made by BMJ to the Submitting Author unless you are acting as an employee on behalf of your employer or a postgraduate student of an affiliated institution which is paying any applicable article publishing charge ("APC") for Open Access articles. Where the Submitting Author wishes to make the Work available on an Open Access basis (and intends to pay the relevant APC), the terms of reuse of such Open Access shall be governed by a Creative Commons licence – details of these licences and which Creative Commons licence will apply to this Work are set out in our licence referred to above.

Other than as permitted in any relevant BMJ Author's Self Archiving Policies, I confirm this Work has not been accepted for publication elsewhere, is not being considered for publication elsewhere and does not duplicate material already published. I confirm all authors consent to publication of this Work and authorise the granting of this licence.

For peer review only

Sodium and potassium intake, and knowledge attitudes and behaviour towards salt consumption: a national survey of adults in the Sultanate of Oman

Adhra Al-Mawali[1,2], Lanfranco D'Elia[3,4], Sathish Kumar Jayapal[1], Magdi Morsi[1], Waleed Nasser Al-Shekaili[1], Avinash D Pinto[1], Hilal Al-Kharusi[1], Zainab Al Bulushi[1], John Idikula[1], Ayaman Al Harrasi[1] and Francesco P Cappuccio[3,5,6].

[1]Centre of Studies & Research, Ministry of Health, Muscat, Sultanate of Oman
[2] The Research Council , Sultanate of Oman
[3]WHO Collaborating Centre for Nutrition, University of Warwick, Coventry, UK
[4]Department of Clinical Medicine and Surgery, "Federico II" University of Naples Medical School, Naples, Italy.
[5]Division of Health Sciences, Warwick Medical School, University of Warwick, Coventry, UK
[6]University Hospitals Coventry & Warwickshire NHS Trust, Coventry, UK

Keywords: Sultanate of Oman; salt; sodium; potassium; population.
Short title: Sodium and potassium intake in Oman
Word count: 3,526

Correspondence to:

Prof. Francesco P Cappuccio
WHO Collaborating Centre for Nutrition
University of Warwick, Warwick Medical School, Division of Health Sciences
Gibbet Hill Road, Coventry CV4 7AL, UK
f.p.cappuccio@warwick.ac.uk; Tel.: +442476573129

For peer review only - http://bmjopen.bmj.com/site/about/guidelines.xhtml

ABSTRACT

Objectives. To estimate population sodium and potassium intakes, and explore knowledge, attitudes and behaviour (KAB) towards the use of salt.

Design. National cross-sectional population-based survey.

Setting. Proportional random samples, representative of Omani adults (18 years or older), were obtained from all Governorates of the Sultanate of Oman.

Participants. Five hundred and sixty-nine (193 men, 376 women18 years or older) were included in the analysis (response rate 57%). Mean age was 39.4 years (SD 13.1). They attended a screening including demographic, anthropometric and physical measurements.

Primary and secondary outcome measures. We assessed dietary sodium, potassium and creatinine by 24h urinary sodium (UNa), potassium (UK) and creatinine (UCr) excretions. We collected KAB by questionnaire.

Results. Mean UNa was 144.3 (78.8) mmoL/day, equivalent to 9.0 g of salt/day and potassium excretion 52.6 (32.6) mmoL/day, equivalent to 2.36 g/day. Men ate more sodium and potassium than women did. Only 22% of the sample had a salt intake below the World Health Organization (WHO) recommended target of 5 g/day and less than a quarter met WHO targets for potassium excretion (>90 mmoL/day). Whilst 89.1% of those interviewed knew that consuming too much salt could cause serious health problems and only 6.9% felt they were using too much added salt, 1 in 2 participants used always or often salt, salty seasonings or salty sauces in cooking or when preparing food at home.

Conclusions. In the Sultanate of Oman, salt consumption is higher, and potassium consumption lower, than recommended by WHO, both in men and in women. The present data provides, for the first time, evidence to back up a national programme of population salt reduction to prevent the increasing burden of CVD in the area.

Article summary

Strengths and Limitations of the study

- This is the first study to measure salt and potassium consumption in men and women in the Sultanate of Oman, using 24h urine collections, and to explore knowledge attitudes and behaviour towards the use of salt.
- The study is nationally representative of all Omani men and women.
- The study has adopted a stringent quality control process to minimize the use of incomplete urine collections.
- The overall response rate was 57%, which is comparable with other similar population surveys.
- Whilst we cannot rule out the risk of selection bias, limiting the overall generalizability of the results, the non-responders did not differ substantially in their baseline characteristics from the responders.

Funding statement

The Ministry of Health of the Sultanate of Oman and the EMRO Regional Office of the World Health Organization supported the study.

Competing interests

AA-M, MM, ADP, HA-K, WNA-S, AA-H are all staff of the Ministry of Health of the Sultanate of Oman. FPC is a technical advisor to the World Health Organization, unpaid member of Action on Salt and WASH. LD was a technical advisor to the World Health Organization and is a member of the Scientific Committee of the Italian Society of Human Nutrition.

For peer review only - http://bmjopen.bmj.com/site/about/guidelines.xhtml

INTRODUCTION

Non-communicable diseases (NCDs) are the leading, yet preventable, causes of death worldwide[1]. The reduction of its burden is now a global health priority of the United Nations[2], endorsed by the World Health Organization (WHO) Action Plan that has identified a set of cost-effective policy options ('best buys'), of which reduction in population salt consumption is one[3].

In the Sultanate of Oman, NCDs are amongst the leading causes of death, accounting for 72% of all deaths[4]. Cardiovascular disease (CVD) represents an increasingly common cause of population morbidity and mortality, accounting for 36% of all deaths[4]. It represents a major public health challenge undermining socio-economic development[5].

High blood pressure (BP) and unhealthy diets are the leading risk factors for CVD in the world[1]. Raised BP is a determinant of the CVD risk in the Sultanate of Oman, where the prevalence of raised blood pressure in people aged 18 years or older is 33%, higher in men (39%) than in women (27%)[5-6].

High salt (i.e., sodium chloride, 1 g = 17.1 mmoL of sodium) consumption is an important determinant of high BP. A high salt intake is associated with raised BP that leads to increased risk of vascular diseases[7-10]. In addition, high salt intake is related to adverse health effects independent of its effects on BP[11-13]. A moderate reduction in salt consumption reduces BP[7-8] and it can improve the health outcomes and indirectly reduce the overall mortality through beneficial effect on the BP[9-10].

The World Health Organization (WHO) recommends that adults should consume no more than 5 g of salt daily[14]. However, mean daily intakes of salt in most of the countries in the world exceed this recommendation[15-16]. Whilst there is no definitive estimate of population dietary salt intake in the Sultanate of Oman, average consumption could be high, similar to some countries in the sub-region[17-18]. In the Sultanate of Oman it is a common habit to add salt and salty condiments to food at the table and when cooking, and the habit of eating out is increasing, especially in urban areas. Salt reduction strategies in the Eastern Mediterranean region, including the Sultanate of Oman, encompass monitoring and evaluation actions as one of their important pillars[19].

In contrast to sodium, epidemiological and intervention studies suggest beneficial effects of dietary potassium on BP and cardiovascular health[20-22]. The Sultanate of Oman lacks data on actual potassium consumption. The WHO currently recommends that adults should consume not less than 90 mmoL of potassium daily[23]. Hence, we need reliable data on sodium and potassium intake in the Sultanate of Oman.

The primary aim of the present study was to establish current baseline average consumption of sodium and potassium by 24h urine collection, in a national random sample of Omani men and women. The study also aimed to explore knowledge, attitudes and behaviour towards dietary salt.

MATERIAL AND METHODS

Participants and Recruitment

We nested the salt survey within the main Oman NCD survey of 6,833 households (Supplementary material). We recruited only one member per household. We designed the salt survey to collect 24h urinary samples from a subgroup of at least 90 participants from each Governorate. The survey included Omani citizens only. We included a total of 999 randomly selected Omani men and women. They were all aged 18 years or older. They comprised residents of all Governorates of the Sultanate of Oman (Table 1).

From the sampling frame and according to the EMRO-WHO Protocol[24], we excluded the following groups: people unable to provide informed consent, those with known history of heart or kidney failure, stroke, liver disease, those who recently began therapy with diuretics (less than two weeks), pregnant women, any other conditions that would make 24h urine collection difficult.

The survey took place between December 2017 and May 2018. From the 999 individuals interviewed in the sampling frame, 569 of them (57.0%) provided suitable data for inclusion in the survey analysis. Originally, 262 (26.0%) did not provide complete urine collections, 87 (8.7%) had missing data, 48 (4.8%) provided urine collections with volume less than 500 mL (conventionally taken as not plausible), and 24 (2.4%) had urinary creatinine excretion outside 2 standard deviations (SDs) of the sex-specific distribution of urinary creatinine in the sample (Figure 1).

Ethical considerations

We carried out the survey in accordance with the Declaration of Helsinki and Good Clinical Practice[25]. We obtained ethical approval for the survey from the Research and Ethics Review and Approval Committee (RERAC) of the Ministry of Health of the Sultanate of Oman and participants provided written informed consent to take part.

Patient and Public Involvement

No patient involved

Data Collection

We performed the examination in a quiet and comfortable room, with the participants who did not smoke, exercise, eat, and consume caffeine before attending, and had a full bladder for 30 minutes before measurements. We carried out the survey in three steps: a) questionnaire survey, b) physical measurements and c) 24h urine collections.

We based the questionnaire on the Core and Expanded version of the WHO STEPS Instrument for Chronic Disease Risk Factor Surveillance[26] and country-specific requirements. It contained 11 Core, 1 Optional and 4 country-specific modules, to determine socio-demographic characteristics of participants, key behavioural risk factors (tobacco use, harmful alcohol consumption, diet with frequency of fruit and vegetable consumption, high dietary salt consumption, oil and fat use, physical inactivity), knowledge attitudes and behaviour on dietary salt, given lifestyle advises, and additional health-related information not presented here.

We measured anthropometric indices, BP and heart rate in all participants. Height was in cm and body weight in kg using a Standardized and calibrated SECA®813 digital floor scales and 213 portable stadiometers, respectively. Body mass index (BMI) was calculated as weight (kg) divided by height squared (m^2). Waist and hip circumferences were measured by a non-stretch SECA®201 measuring tape to the nearest mm[24]. We took systolic and diastolic BP and heart rate measurements three times in the right arm on a sitting position, using an appropriate cuff and a validated digital device (OMRON M3). We ignored the first measurement, and used the mean of second and third measurements for analysis. We took measurements after the participant had rested for 15 minutes and each with three minutes of rest between the measurements (maximum deviation of cuff pressure measurement ± 3 mmHg and of pulse rate display ± 5%). Hypertension is defined as systolic and/or diastolic BP ≥ 140/90 mmHg or regular antihypertensive treatment[27]. We obtained a single 24h urine collection from the participants. We gave each participant a leaflet with explanations along with the necessary equipment and a record sheet on which participant noted the start and the finish times of their urine collection, any missed urine aliquots and any medication taken during the collection. We instructed the participants carefully on urine collection methodology[24]. In an effort to minimize bias, we also requested participants not to change their diet before or during the day of the urine collection. They discarded the first void upon waking on the day of collection. Participants then filled the 24-hour urine container over the 24-hour period. On the following day, the field team-members measured total volume, mixed it thoroughly, and obtained a urine sample, which was kept in a cool-box for transport to the respective laboratory. On arrival at the laboratory, we either carried out determinations immediately or

For peer review only - http://bmjopen.bmj.com/site/about/guidelines.xhtml

stored samples in the fridge until the determination (as soon as possible). We determined sodium, potassium and creatinine immediately. Sodium and potassium concentration in the urine samples were determined using an Ion Selective Electrode with an Abott C8000 & Roche Cobas 6000 and expressed in mmol/L[28]. Creatinine concentration was determined through either the kinetic (Abbott C8000) or enzymatic (Roche Cobas 6000) method and expressed in mg/dL[29]. These determinations were carried out in one reference laboratory in each of the 11 governorates, except for 2 regions (Dhofar and Musandam) which had 2 receiving reference laboratories each. All laboratories underwent joint quality control.

Statistical Analysis

We performed all statistical analyses using the SPSS software, version 20 (SPSS Inc., Chicago, IL, USA). To detect approximately 1 g reduction in salt intake over time using 24h urinary sodium excretion (difference ~20 mmoL/24h), with a standard deviation of 75 mmoL/day (alpha = 0.05, power = 0.80), a minimum sample of 120 individuals per stratum is recommended[24]. Thus, we estimated a minimum recommended sample size of 240 per age and sex groups and adjusted for an anticipated non-response rate of 50%[24]. We stratified the population in groups by sex (men and women). Therefore 480 individuals were originally needed to be selected (total n=120*2 groups/0.5 attrition=480). We used T-test for unpaired samples or analysis of variance (ANOVA) to assess differences between group means and Pearson chi-square test to test the association between categorical variables. To convert urinary output into dietary intake, we first converted the urinary excretion of sodium (UNa) or potassium (UK) values (mmoL/day) into mg/day (for sodium 1 mmol = 23 mg of sodium, for potassium 1 mmol = 39 mg). We then multiplied the sodium value by 2.542 to convert dietary sodium (Na) intake into salt (NaCl) intake. We finally multiplied sodium values by 1.05 (assuming that approximately 95% of sodium ingested is excreted)[30]. We calculated potassium dietary intake assuming that 80% of the potassium ingested is excreted in the urine[31]. The results were reported as mean (SD and/or 95%CI) or as percentages, as appropriate. We considered two-sided p below 0.05 as statistically significant.

RESULTS

The final population sample included 569 participants between 18 and 69 years old (n = 193 or 34% men and n = 376 or 66% women), recruited nationally.

For peer review only - http://bmjopen.bmj.com/site/about/guidelines.xhtml

Characteristics of the Participants

Table 2 shows the characteristics of the participants. There was no statistically significant difference in the mean age and in body mass index between men and women, however men had significantly higher BP and slower heart rate than women did. The prevalence of hypertension was on average 27.4%, significantly higher in men than in women (38.5% v 21.7%, $p<0.001$).

Daily Urinary Excretions of Volume, Sodium, Potassium and Creatinine and Salt and Potassium Intake

Average urinary volume excretion was 1354 mL per day, being higher in men than women (Table 3). Average urinary creatinine excretion was 1.33 g per day, being again higher in men than women (Table 3). Urinary sodium excretion showed a normal distribution with a tail skewed to the right (i.e., towards higher values). Mean urinary sodium was 144.3 (SD 78.8) mmoL/24h (Table 3), equivalent to a mean consumption of 9.0 (4.9) g of salt per day (Table 3). Men excreted more sodium than women did (mean difference 15.0 mmoL/24h, $p < 0.05$), equivalent to ~1.0 g of higher salt consumption than women did. Only 22% of the participants met the levels of salt intake of 5 g or less recommended by the WHO, with no difference between sexes. Urinary potassium excretion showed a normal distribution with a tail skewed to the right (i.e. towards higher values). Mean urinary potassium was 52.6 (32.6) (Table 3), equivalent to a mean consumption of 2.36 (1.46) g of potassium per day (Table 3).

Men excreted more potassium than women did; 22.5% of participants met the levels of potassium intake of 90 mmoL/day or more recommended by the WHO, with no difference between sexes.

Knowledge, Attitude and Behaviours towards Salt Intake and other eating patterns.

Knowledge, attitude and behaviours toward the consumption of salt was assessed by asking participants about the frequency, quantity and type of salt used in the household, as well as their cooking habits and their attitudes towards dietary salt (Table 4). A total of 28.1% of respondents mentioned that they added salt or salty sauces always or often to food. The percentage of women who added salt or salty sauces always or often to their meal was significantly higher than that of men (30.8% vs 22.8%; p=0.005). A total of 47.0% of respondents reported that they always or often added salt, salty seasonings or sauces when cooking or preparing food at home, women more than men (48.1% vs 44.8%; p=0.04). More than 1 in 5 (22.3%) mentioned that they consumed processed foods high in salt always or often. Very few (6.9%), however, felt they consumed too much salt or salty sauces, although 89.1% knew that

For peer review only - http://bmjopen.bmj.com/site/about/guidelines.xhtml

consuming too much salt could cause serious health problems. We also asked participants about dietary attitudes about the consumption of fruit and vegetables, oil or fats (Table 5). Interestingly, 68.0% consumed fruit at least five days a week, and 54.5% at least three servings on these days. Men appeared to report more fruit consumption than women did (40.4% vs 27.7%; p=0.002). Vegetables were also consumed frequently (75.9% at least 5 days a week), with 40.1% having at least 3 servings on one of those days (women more frequently than men). The majority (90.8%) used vegetable oil for meal preparation in the household and more than half (54.2%) consumed food prepared outside home at least once a week. Men were more likely than women to do so (67.9% vs 47.2%; p<0.001).

DISCUSSION

This the first nationally representative population-based survey carried out in the Sultanate of Oman assessing dietary sodium and potassium consumption in adult Omani men and women, using the gold standard measure of 24h urinary sodium and potassium excretions as a biomarker of intake. The results show that salt consumption is higher and potassium consumption is lower than recommended by the World Health Organization[14,23], both in men and women. Average population salt consumption was 9.0 g per day, almost double the World Health Organization recommended maximum population target of 5 g per day[14]. Less than 1 in 4 participants met these targets. Salt consumption varied across Governorates, being the lowest in South Sharqiah (5.3 g per day) and the highest in Al-Dhahirah (14.8 g per day). Average population excretion of potassium was 53 mmol per day (equivalent to about 2.36 g per day), lower than the World Health Organization recommended maximum population target of >90 mmol per day, equivalent to approx. 3.90 g per day (assuming urinary potassium being 85% of the intake)[23]. Potassium consumption also varied across Governorates, being the lowest in Al-Wasta (1.41 g per day) and the highest in North Sharqiah (4.25 g per day).

Our questionnaire revealed that half of the population seen often used sauces and condiments (invariably containing high concentrations of salt) but only 10% believed this was too much. A quarter of the surveyed population added salt to food regularly, one in five ate processed food often and more than half of the population ate out at least once a week, with men more likely than women. These results, in addition to those obtained in previous surveys on unhealthy dietary habits, support the national Health Vision set by the Sultanate of Oman to reduce the burden of CVD[32].

For peer review only - http://bmjopen.bmj.com/site/about/guidelines.xhtml

Comparison with countries of the Gulf Cooperation Council (GCC) and of the Arab Peninsula. In the Gulf Cooperation Council (GCC) countries, populations lead a sedentary lifestyle, both hypertension and obesity are common[17] and they are major contributors to NCDs[33]. The estimated total mortality in GCC countries attributable to NCDs vary from 65% to 78%, with the highest estimates in Bahrain and Saudi Arabia and the lowest in Oman and Qatar, respectively[33]. Salt intake is deemed high in most countries of the Eastern Mediterranean Region (EMR), although there are only a few studies that directly measured population levels, with inconsistent results due to methodological inadequacies[17.18]. The Global Burden of Disease (GBD) estimates of average salt consumption using a Bayesian model suggest that salt consumption in GCC countries may vary from 8.0 g per day in Saudi Arabia to 13.5 g per day in Bahrain[34]. Estimates of salt intake in neighbouring countries would also range between 7.8 g per day in Lebanon and 10.3 g per day in Jordan[34]. The present study is one of the few nationally representative surveys in GCC countries using the gold standard method of assessment of dietary salt intake. Its results suggest an intake close to that estimated by the GBD. In addition to the GBD, however, our study also provides, for the first time, direct measures of average population potassium consumption, also targeted by WHO recommendations for cardiovascular prevention[20,23].

Strengths and Limitations

Our study has several strengths. First, it is population-based survey across the whole country. Second, it included all adults. Thirds, it included both men and women. These study characteristics would allow with greater confidence the extrapolation of results to the whole country population, rather than those conducted in selected groups including patients[35], young female University students[36] or children[37]. Fourth, it used the current preferred methodology for estimating salt consumption. Fifth, we applied a rigorous quality control protocol to ensure completeness of urine collections, and to minimize both under and over-estimations. Current recommendations suggest the use of single complete 24-hour urine samples, collected from a representative population sample to assess the population's current 24-hour dietary sodium ingestion[38]. The role of single spot or short duration timed urine collections in assessing population average sodium intake requires more research. Single or multiple spot or short duration timed urine collections are, on the other hand, not recommended for assessing an individual's sodium intake especially in relationship to health outcomes[38]. Twenty-four hour diet recall and diet records inaccurately measure dietary sodium intake in individuals compared with the gold standard 24 hours urinary excretion[39]. Furthermore, there is poor agreement between estimates of

For peer review only - http://bmjopen.bmj.com/site/about/guidelines.xhtml

sodium intake from food-frequency questionnaires and 24-hour urine samples[40]. Sixth, it has measured directly the amount of potassium consumption, additional nutrient targeted for cardiovascular prevention[20,23]. Seventh, we standardised fieldwork and used standardised laboratory methodologies across the country. Eighth, all laboratories underwent joint quality control[25].

There are limitations too. First, we analysed only 57% of the urine samples originally collected from willing individuals. This was due to the stringent quality control that has led to the exclusion of incomplete or erroneous collections[24]. This could have introduced a self-selection bias. The comparison of the baseline characteristics of the studies sample versus the excluded group suggests that the two groups were comparable for general characteristics, with the exception of the latter being two years younger and having a 1.8 mmHg lower diastolic blood pressure. Second, we assessed urinary sodium and potassium excretions only once. Whilst we cannot characterise an individual's intake in such a way[38], there is less likelihood of a bias of group estimates. Third, although we administered a questionnaire to derive knowledge, attitudes and behaviours towards the use of salt, we were unable to establish the relative contribution of discretionary sources of salt and commonest foods contributing to salt as well as potassium consumption.

Potential impact

The population in the Sultanate of Oman is of just over 5 million (Ministry of Health Annual Health Report, 2018 estimates), of which about 2.3 million are Omani nationals[4] (surveyed in the present study). Approximately 51% are 25 years or older. To meet a 30% reduction in population salt consumption set by WHO by 2025, the Sultanate of Oman should aim at a 2.7 g per day salt reduction nationally. This reduction would avert 8.1% CVD deaths per year, and more non-fatal events and disabilities[2].

Policy Implications

The Sultanate of Oman has embraced amongst its health priorities the prevention and control of noncommunicable diseases and improvement in nutrition[4] in line with the strategic directions of WHO endorsed by the Eastern Mediterranean Region (EMRO) in 2012 and 2013[18]. Since then several countries have conducted dietary assessment studies in an attempt to assess the population's salt intake[18]. Studies in the area have also attempted to identify the major dietary contributors to sodium intake. Studies are still limited and there are large variations in dietary habits in the Region due to cultural, ethnic, religious and social heterogeneity. The commonest source of salt

For peer review only - http://bmjopen.bmj.com/site/about/guidelines.xhtml

consumption across the Region is bread[18,41], in all its different forms, with other sources being more relevant in different countries. In Lebanon[42] and in Bahrain[43], dairy products are common sources, whilst in Morocco[44] major contributors to salt consumption include cereal-based products, spices and condiments and milk products. These indications, together with the awareness and behaviours measured, suggest that policy priorities[45] to reduce population salt consumption in the Sultanate of Oman would require (a) improvement of salt-related knowledge through health promotion campaigns, (b) assessment of major sources of salt consumption, (c) establishing collaborations with the local authorities to reduce the amount of salt used in traditional bread making and other identified sources like locally produced condiments, (d) adopting a labelling strategy for imported foods with high salt content. The policy priorities will be adapted in the Sultanate of Oman. In addition, the Ministry of Health will develop strategies and methodologies to measure the indicators of population salt consumption[45].

Conclusions

This study demonstrates that salt consumption in the Sultanate of Oman is high and should be reduced through a public health action aiming at the entire population. Education of the dangers of high salt consumption and where salt is hidden, accurate labelling and marketing of food, surveillance to measure and monitor salt use, creation of a healthy environment for adults as well as children, driving reformulation of some commonly consumed foods (like bread) are all important elements of an effective national salt reduction programme[18,19,46].

Supplementary materials: The following are available online at

Table S1. Characteristics of the excluded participants and comparison with those included in the study. **Text S1**: Material and Methods (full text)

Acknowledgments: We carried out the present analysis under the terms of reference of the WHO Collaborating Centre for Nutrition at the University of Warwick. The authors would like to express gratitude to the EMRO WHO Regional Office (Dr Ayoub Al-Jawaldeh) and the WHO Country Office of Oman (Dr Ruth Mabry) for facilitating the study, the Department of Food Science and Human Nutrition of Sultan Qaboos University (Dr Lyutha Al-Subhi) and other members of the Ministry of Health (Dr Amel Ibrahim, Dr Ruqaya Balushi and Dr Salima for their helpful discussions in the preparation of the protocol. Special thanks go to the team of the Ministry of Health of the Sultanate of Oman involved in the preparation of the survey and data collection.

For peer review only - http://bmjopen.bmj.com/site/about/guidelines.xhtml

Finally, we wish to express our gratitude to His Excellency the Undersecretary for Health Affairs of the Sultanate of Oman, Dr Mohammed bin Saif Al Hosni, and His Advisor, Dr Mahmood Shaban, for their advice, endorsements and support at a National level.

Author Contributions: FPC developed the study design and protocol, contributed to the analysis and drafted the manuscript, AA-M trained local teams, co-ordinated quality control and data collection. MM and LD carried out quality control and statistical analysis. AA-M, ADP, HA-K, WNA-S coordinated the study, carried out the fieldwork and liaised with the local laboratory. MM helped with the drawing of the stratified random sample from the sampling frame. All authors contributed to the interpretation of the findings and they contributed significantly to the final version of the manuscript.

Data Sharing Statement: No individual participant data will be available. Study protocol available in Supplementary Material. Any other data sharing proposal must be submitted in writing to the Ministry of Health of the Sultanate of Oman.

References

1. GBD 2015 Mortality and Causes of Death Collaborators. Global, regional, and national life expectancy, all-cause mortality, and cause-specific mortality for 249 causes of death, 1980–2015: A systematic analysis for the Global Burden of Disease Study 2015. *Lancet* 2016; 388: 1459–1544.
2. World Health Organization. Global Action Plan for the Prevention and Control of NCDs 2013–2020; World Health Organization: Geneva, Switzerland, 2013; pp. 1–103.
3. World Health Organization. Tackling NCDs: “Best buys” and other recommended interventions for the prevention and control of noncommunicable diseases; 2017. http://www.who.int/ncds/management/best-buys/en/
4. World Health Organization. Oman Health Profile 2018. World Health Organization. Regional Office for the Eastern Mediterranean, Cairo, Egypt, 2016; pp. 1-37. WHO-EM/HST/227/E
5. Al-Mawali A. Non-communicable diseases: shining a light on cardiovascular disease, Oman’s biggest killer. *Oman Med J* 2015; 30(4): 227-228.
6. Al Riyami A, Elaty MAA, Morsi M, et al. Oman World Health Survey: Part 1: Methodology, sociodemographic profile and epidemiology of non-communicable diseases in Oman. *Oman Med J* 2012; 27(5): 425-443.

7. Aburto NJ, Ziolkovska A, Hooper L, Elliott P, Cappuccio FP, Meerpohl J. Effect of lower sodium intake on health: Systematic review and meta-analysis. *Br. Med. J.* 2013; 346: f1326.
8. He FJ, Li J, MacGregor GA. Effect of longer-term modest salt reduction on blood pressure: Cochrane systematic review and meta-analysis of randomised trials. *Br. Med. J.* 2013; 346: f1325.
9. Strazzullo P, D'Elia L, Kandala N.-B, Cappuccio FP. Salt intake, stroke, and cardiovascular disease: Meta-analysis of prospective studies. *Br. Med. J.* 2009; 339: b4567.
10. Cook NR, Cutler JA, Obarzanek E, et al. Long term effects of dietary sodium reduction on cardiovascular disease outcomes: Observational follow-up of the Trials of Hypertension Prevention (TOHP). *Br. Med. J.* 2007; 334: 885–888.
11. Cappuccio FP. Cardiovascular and other effects of salt consumption. *Kidney Int.* 2013; 3: 312–315.
12. D'Elia L, Rossi G, Schiano di Cola M, et al. Meta-analysis of the effect of dietary sodium restriction with or without concomitant renin–angiotensin–aldosterone system-inhibiting treatment on albuminuria. *Clin. J. Am. Soc. Nephrol.* 2015; 10: 1542–1552.
13. D'Elia L, Galletti F, La Fata E, Sabino P, Strazzullo P. Effect of dietary sodium restriction on arterial stiffness: Systematic review and meta-analysis of the randomized controlled trials. *J. Hypertens.* 2018; 36: 734–743.
14. World Health Organization. Guideline. Sodium Intake for Adults and Children. World Health Organization (WHO): Geneva, Switzerland, 2012.
15. Brown IJ, Tzoulaki I, Candeias V, Elliott P. Salt intakes around the world: Implications for public health. *Int. J. Epidemiol.* 2009; 38: 791–813.
16. Cappuccio FP, Capewell S. Facts, issues, and controversies in salt reduction for the prevention of cardiovascular disease. *Funct. Food Rev.* 2015; 7: 41–61
17. Alhamad N, Almalt E, Alamir N, Subhakaran M. An overview of salt intake reduction efforts in the Gulf Cooperation Council countries. *Cardiovasc. Diagn. Ther.* 2015; 5(3): 172-177.
18. Al Jawaldeh A, Rafii B, Nasreddine L. Salt intake reduction strategies in the East Mediterranean Region. *East Mediterr. Health J.* 2018; 24(12): 1172-1180.
19. Cappuccio FP, Capewell S, Lincoln P, McPherson K. Policy options to reduce population salt intake. *Br. Med. J.* 2011; 343: 402–405.
20. Aburto NJ, Hanson S, Gutierrez H, Hooper L, Elliott P, Cappuccio FP. Effect of increased potassium intake on cardiovascular risk factors and disease: Systematic review and meta-analyses. *Br. Med. J.* 2013; 346: f1378.

For peer review only - http://bmjopen.bmj.com/site/about/guidelines.xhtml

21. D'Elia L, Barba G, Cappuccio, FP, Strazzullo P. Potassium Intake, Stroke, and Cardiovascular Disease. A meta-analysis of Prospective Studies. *J. Am. Coll. Cardiol.* 2011; 57: 1210–1219.
22. D'Elia L, Iannotta C, Sabino P, Ippolito R. Potassium rich-diet and risk of stroke: Updated meta-analysis. *Nutr. Metab. Cardiovasc. Dis.* 2014; 24: 585–587.
23. World Health Organization. Guideline: Potassium Intake for Adults and Children. World Health Organization (WHO): Geneva, Switzerland, 2012; pp. 1–52.
24. World Health Organization. How to Obtain Measures of Population-Level Sodium Intake in 24-Hour Urine Samples. World Health Organization. Regional Office of the Eastern Mediterranean, Cairo, Egypt, 2018; pp. 1–51. WHO-EM/NUT/279/E.
25. World Medical Association. Declaration of Helsinki. Recommendations guiding doctors in clinical research. *Bull. World Health Org.* 2008; 86: 650–651.
26. World Health Organization. The WHO STEPwise approach to chronic disease risk factor surveillance (STEPS). World Health Organization, Geneva, Switzerland, 2014. https://www.who.int/ncds/surveillance/steps/STEPS_Instrument_v2.1.pdf
27. Mancia G, Fagard R, Narkiewicz K, et al. 2013 ESH/ESC Guidelines for the management of arterial hypertension: The Task Force for the management of arterial hypertension of the European Society of Hypertension (ESH) and of the European Society of Cardiology (ESC). *J. Hypertens.* 2013; 31: 1281–1357.
28. Oesch U, Ammann D, Simon W. Ion-selective membrane electrodes for clinical use. *Clin. Chem.* 1986; 32: 1448–1459.
29. Junge W, Wilke B, Halabi A, Klein G. Determination of reference intervals for serum creatinine, creatinine excretion and creatinine clearance with an enzymatic and a modified Jaffé method. *Clin. Chim. Acta* 2004; 344: 137–148.
30. Lucko AM, Doktorchik C, Woodward M, et al. Percentage of ingested sodium excreted in 24-hour urine collections: A systematic review and meta-analysis. *J Clin. Hypertens.* 2018; 20: 1220–1229.
31. Stamler J, Elliott P, Chan Q, for the INTERMAP Research group. INTERMAP Appendix tables. *J. Hum. Hypertens.* 2003; 17: 665–758.
32. Ministry of Health. *Health Vision 2050. The Main Document*. Undersecretariat for Planning Affairs, Ministry of Health, Sultanate of Oman 2014; pp. 1-212. http://www.nationalplanningcycles.org/sites/default/files/planning_cycle_repository/oman/health_vision_2050_oman.pdf (accessed Jan 14, 2020).
33. World Health Organization. Noncommunicable Diseases Country Health Profiles, 2019. http://www.emro.who.int/entity/statistics/country-health-profiles.html

For peer review only - http://bmjopen.bmj.com/site/about/guidelines.xhtml

34. Powles J, Fahimi S, Micha R, et al. Global, regional and national sodium intakes in 1990 and 2010: a systematic analysis of 24h urinary sodium excretion and dietary survey worldwide. *BMJ Open* 2013; 3: e003733.
35. Alkhunaizi AM, Al Jishi HA, Al Sadah ZA. Salt intake in Eastern Saudi Arabia. *East Mediterr. Health J.* 2013; 19(11): 915-918.
36. Tayel DI, Amine AK, Elzawi AK. Dietary intake of nutrients related to bone health among Alexandria University female students, Egypt. *Food and Public Health* 2013; 3(6): 329-335.
37. Kelishadi R, Gheisari A, Zare N, Farajian S, Shariatinejad K. Salt intake and the association with blood pressure in young Iranian children: first report from the Middle East and North Africa. *Int. J. Prev. Med.* 2013; 4(4): 475-483.
38. Campbell NRC, He FJ, Tan M, et al. The International Consortium for Quality Research on Dietary Sodium/Salt (TRUE) position statement on the use of 24-hour, spot and short duration (<24-hours) timed urine collections to assess dietary sodium intake. *J Clin Hypertens* 2019; 21: 700-709.
39. McLean RM, Farmer VL, Nettleton A, et al. Twenty-Four– Hour Diet recall and Diet records compared with 24-hour urinary excretion to predict an individual's sodium consumption: A Systematic Review. *J Clin Hypertens* 2018; 20: 1360–1376.
40. McLean RM, Farmer VL, Nettleton A, et al. Assessment of dietary sodium intake using a food frequency questionnaire and 24-hour urinary sodium excretion: a systematic literature review. J *Clin Hypertens* 2017; 19: 1214–1230.
41. Almedawar MM, Nasreddine L, Olabi A, et al. Sodium intake reduction efforts in Lebanon. *Cardiovasc Diagn Ther* 2015; 5(3): 178-185.
42. Nasreddine L, Akl C, Al-Shaar L, Almedawar MM, Isma'eel H. Consumer knowledge, attitudes and salt-reated behaviour in the Middle-East: the case of Lebanon. *Nutrients* 2014; 6: 5079-5102.
43. Ministry of Health (Bahrain). National nutrition survey for adult Bahrainis aged 19 years and above. 2002
44. Derouiche A, Jafri A, El Kardi Y. Assessment of salt, fat content, and intake in largely consumed foods in Morocco. Report of a pilot study. 2014; p. 1-92
45. Bonita R, Magnusson R, Bovet P, et al. Country actions to meet UN commitments on non-communicable diseases: a stepwise approach. *Lancet* 2013; 381: 575-584.
46. Farrand C, He FJ, MacGregor GA. Reducing population salt intake in the Eastern Mediterranean Region – time for urgent action. *Eastern Med. Health J.* 2014; 20(12): 761-764.

Figure 1. Stepwise procedure for the selection of valid participants according to protocol adherence, quality control and completeness of 24h urine collections.

For peer review only - http://bmjopen.bmj.com/site/about/guidelines.xhtml

Table 1. Geographical sampling from the Sultanate of Oman.

**Governorate**	**Valid 24h urine collections**	**%**
Muscat	67	11.8
Dhofar	79	13.9
Al-Dakhlia	45	7.9
North Sharqiah	36	6.3
South Sharqiah	45	7.9
North Batina	81	14.2
South Batina	53	9.3
Al-Dhahirah	46	8.1
Al Buraymi	84	14.8
Musandam	9	1.6
Al-Wasta	24	4.2
**Total**	**569**	**100.0**

For peer review only - http://bmjopen.bmj.com/site/about/guidelines.xhtml

Table 2. Characteristics of the participants

**Variable**	**All (n=569)**	**Men (n=193)**	**Women (n=376)**
Age (years)	39.4 (13.1)	38.7 (14.3)	39.8 (12.5)
Height (cm) ‡	159.4 (11.2)	167.9 (9.7)	154.9 (9.2)*
Weight (kg) ‡	74.9 (21.5)	81.4 (22.5)	71.4 (20.1)*
BMI (kg/m^2) ‡	29.3 (7.2)	28.9 (7.6)	29.5 (7.0)
Waist circumference (cm) ‡	93.8 (15.7)	95.0 (15.7)	93.2 (15.7)
Hip circumference (cm) ‡	104.5 (15.0)	102.6 (13.7)	105.5 (15.6)†
Systolic blood pressure (mm Hg) ‡	125.9 (18.2)	134.0 (17.0)	121.7 (17.3)*
Diastolic blood pressure (mm Hg) ‡	80.9 (10.7)	83.4 (11.5)	79.7 (10.1)*
Pulse rate (b/min) ‡	79.8 (10.5)	78.5 (11.8)	80.4 (9.8)†
Hypertension (%) ‡	27.4	38.5	21.7*

Results are mean (SD), ‡ reduced numbers due to missing values;
Hypertension: SBP/DBP >=140/90 mmHg

For peer review only - http://bmjopen.bmj.com/site/about/guidelines.xhtml

Table 3. Daily urinary excretions of volume, sodium, potassium and creatinine, estimates of salt and potassium intake, and proportion of participants meeting WHO recommended targets for salt and potassium consumption.

Variable	All (n=569)	Men (n=193)	Women (n=376)
Volume (mL/24h)	1354 (725)	1392 (712)	1335 (731)*
Sodium (mmol/24h)	144.3 (78.8)	154.2 (87.4)	139.2 (73.6)*
Potassium (mmol/24h)	52.6 (32.6)	56.4 (32.4)	50.6 (32.5)*
Creatinine (g/24h)	1.33 (0.71)	1.72 (0.87)	1.13 (0.52)[†]
Salt intake (g/day)	9.0 (4.9)	9.6 (5.5)	8.7 (4.6)*
Potassium intake (g/day)	2.36(1.46)	2.53 (1.45)	2.27 (1.46)*
Salt <5g/day N (%)	124 (21.8)	40 (20.7)	84 (22.3)
Potassium >90 mmol/day N (%)	128 (22.5)	56 (29.0)	72 (19.1)[#]

Results are mean (SD) or N (%); *p<0.05; [#]p=0.008, [†]p<0.001 vs men

For peer review only - http://bmjopen.bmj.com/site/about/guidelines.xhtml

Table 4. Knowledge, attitudes and behaviours towards salt consumption.

**Question**	**All (n=569)**	**Men (n=193)**	**Women (n=376)**
How often do you add salt or salty sauces to your food?			
Often/Always	28.1 %	22.8 %	30.8 % *
Sometimes	21.3 %	17.1 %	23.4 %
Rarely/Never	50.6 %	60.1 %	45.8 %
How often is salt, salty seasoning or salty sauces added in cooking or preparing food at home? ‡			
Often/Always	47.0 %	44.8 %	48.1 % #
Sometimes	16.5 %	12.5 %	18.6 %
Rarely/Never	36.5 %	42.7 %	33.3 %
How often do you eat processed food? ‡			
Often/Always	22.3 %	22.8 %	22.1 %
Sometimes	38.3 %	35.8 %	39.5 %
Rarely/Never	39.4 %	41.4 %	38.4 %
How much salt or salty sauces do you think you consume? ‡			
Too much/Far too much	6.9 %	7.8 %	6.4 %
Just the right amount	66.8 %	61.3 %	69.7 %
Too little/Far too little	26.3 %	30.9 %	23.9 %
Do you think that too much salt or salty sauces could cause a serious health problem? ‡			
Yes	89.1 %	90.1 %	88.6 %

Results are percentages *p=0.005; #p=0.04 vs men. ‡ reduced numbers due to missing values

For peer review only - http://bmjopen.bmj.com/site/about/guidelines.xhtml

Table 5. Frequency of other dietary patterns

Question	All (n=569)	Men (n=193)	Women (n=376)
In a typical week, on how many days do you eat fruit? ‡			
< 5	32.0 %	40.4 %	27.7 %**
≥ 5	68.0 %	59.6 %	72.3 %
How many servings of fruit do you eat on one of those days? ‡			
<3	45.5 %	41.8 %	47.2 %
≥3	54.5 %	58.2 %	52.8 %
In a typical week, on how many days do you eat vegetables? ‡			
< 5	24.1 %	24.9 %	23.7 %
≥ 5	75.9 %	75.1 %	76.3 %
How many servings of vegetables do you eat on one of those days? ‡			
<3	59.9 %	65.9 %	56.9 %#
≥3	40.1 %	34.1 %	43.1 %
What type of oil or fat is most often used for meal preparation in your household? ‡			
Vegetable oil	90.8 %	91.1 %	90.7 %
Other (lard, suet, butter, ghee)	9.0 %	8.9 %	9.0 %
None used	0.2 %	0	0.3 %
On average, how many meals per week do you eat that were not prepared at home? ‡			
0	45.8 %	32.1 %	52.8 %*
≥1	54.2 %	67.9 %	47.2 %

Results are percentages *p<0.0001; **p=0.002; #p=0.04 vs men. ‡ reduced numbers due to missing values

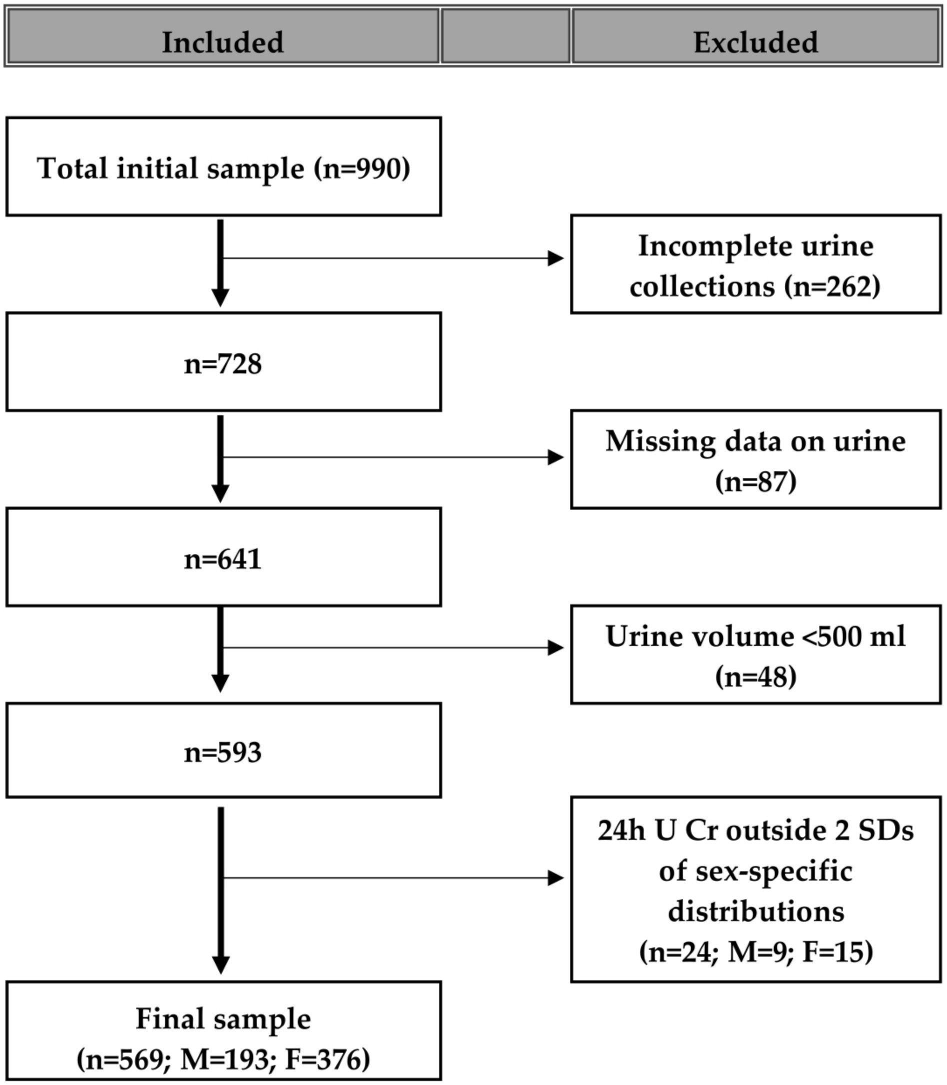

Figure 1. Stepwise procedure for the selection of valid participants according to protocol adherence, quality control and completeness of 24h urine collections.

Supplementary Materials

Table S1. Characteristics of excluded participants and comparison with those included in the final analysis.

Variable	Included (n=569)	Excluded (n=159)	P value*
Age (years)	39.4 (13.1)	37.5 (13.9)	0.037
Height (cm)	159.4 (11.2)	158.7 (8.6)	>0.05
Weight (kg)	74.9 (21.5)	73.4 (18.3)	>0.05
BMI (kg/m^2)	29.3 (7.2)	29.1 (7.0)	>0.05
Waist circumference (cm)	93.8 (15.7)	92.3 (17.9)	>0.05
Hip circumference (cm)	104.5 (15.0)	102.6 (16.4)	>0.05
Systolic blood pressure (mm Hg)	125.9 (18.2)	124.2 (18.8)	>0.05
Diastolic blood pressure (mm Hg)	80.9 (10.7)	79.1 (11.6)	0.025
Pulse rate (b/min)	79.8 (10.5)	80.9 (10.5)	>0.05
Hypertension (%)	27.4	23.9	

Results are mean (SD), *by Mann-Whitney U-test
Hypertension: SBP/DBP >=140/90 mmHg

For peer review only

TEXT S1. MATERIAL AND METHODS

1.1 Study Design

A national cross-sectional population-based survey was conducted using a representative sample of the Sultanate of Oman based on the WHO Stepwise approach to Surveillance (STEPS) of NCD risk factors (1), comprising the following aspects:

- **Step 1:** This consisted of face-to-face interviews using advanced standardized nation-specific version of the STEPS questionnaire and locally-adapted show cards to facilitate the understanding and operationalization of some questions. Socio-demographic characteristics, key behavioural risk factors, lifestyles, eye and ear health, history of chronic diseases and health care coverage were also elicited to better define exposure and health care seeking and control.
- **Step 2:** This involved physical measurements (e.g. weight, height, waist, hip circumference), and determination of blood pressure, heart rate, and vision function to investigate biological risk factors such as hypertension, overweight, obesity, and vision issues.
- **Step 3:** This aimed at determinations of biochemical markers levels (e.g. fasting capillary blood for glucose and lipid profile, and non-fasting urinary samples for sodium and creatinine) to identify hypercholesterolemia, hyperglycaemia, and sodium intake.

1.2 Sample Design

2.1 Target Population: The population of interest was all non-institutionalized persons, which included all men and women 18 years of age or older (Omani & non-Omani) who reside in the country.

2.2 Inclusion population: The target population includes all persons who consider the country to be their usual place of residence. This definition comprises those individuals residing in the country even though they may not be considered a citizen of the country.

2.3 Exclusion population: Those household members who were younger than 18 years of age; persons who have cognitive impairment that hampered understanding the questions to provide clear feedback; visitors (tourists) to the country; and, institutionalized people or those who indicated their usual place of residence was a military base, labour camps or group quarters, were excluded from the survey.

1.3 Sample Frame

For the 2010 National census, the whole of the Sultanate of Oman was divided into 15,077 census blocks. A census block was defined as a collection of units (residential units), which includes 60 units or less, and it could involve one or more enumeration areas. The census block

For peer review only - http://bmjopen.bmj.com/site/about/guidelines.xhtml

is the smallest unit of field work for census data collectors. A total of 399,274 households[1] were identified in these blocks (260,120 Omani and 139,154 non-Omani). Households for Omanis and non-Omanis could not be differentiated unless physically visited as they lived closely together and there were no specific places (blocks) for non-Omani. The number of blocks and households in each governorate was provided in the table below according to the National census of 2010 and the map below shows the distribution of blocks over the Sultanate of Oman by governorates (NCSI). (Figure 1).

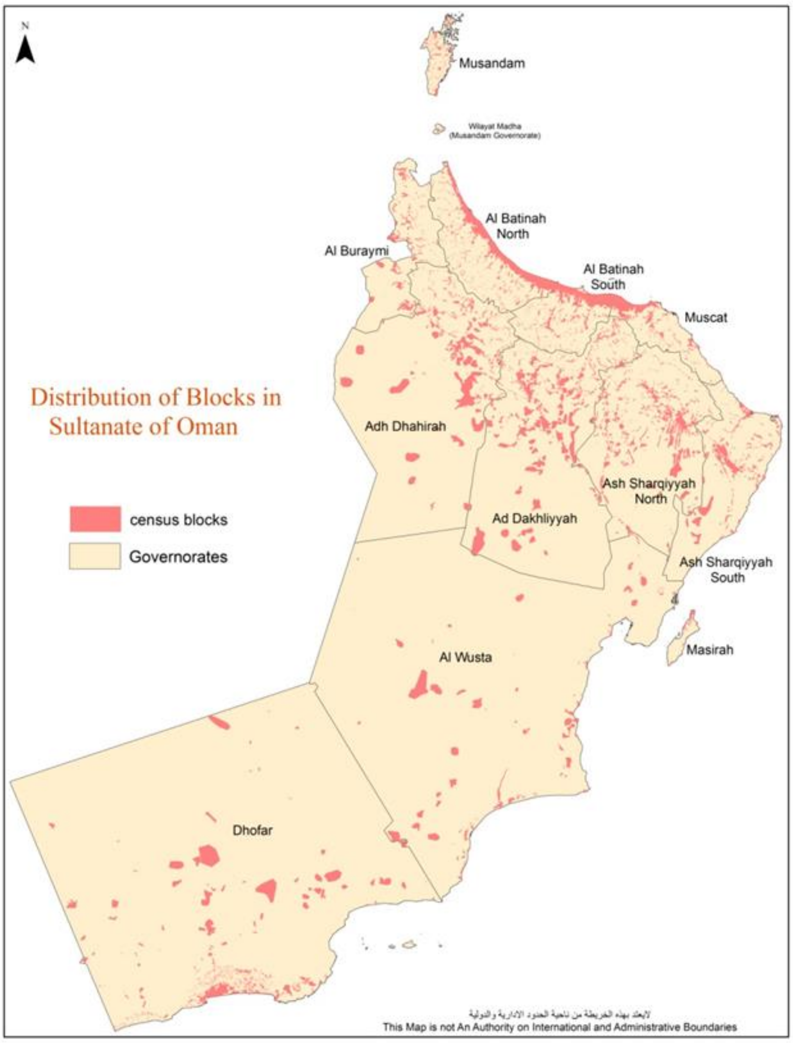

Figure 1: Map of the Sultanate of Oman of census blocks for sampling, by governorate

*Source: National Centre for Statistics and Information (NCSI)

[1] A "Household (HH)" was defined as "either a one-person household, defined as an arrangement in which one person makes provision for his or her own food or other essentials for living without combining with any other person to form part of multi-person household or a multi-person household, defined as a group of two or more persons both related and unrelated living together who make common provision for food or other essentials for living".

The same census Block was updated and used in this NCD Risk Factor Survey also and is as shown in Table 1.

Table 1: As 2010 Number of blocks and households by governorate comprising the sampling frame for Oman NCD risk factors survey, 2017

Governorate	Number of blocks	Number of Omani households
**Muscat**	3514	62299
**Dhofar**	1768	17926
**Ad Dakhiliyah**	1712	34611
**North Sharqiyah**	1173	20044
**South Sharqiyah**	1097	22455
**North Batinah**	2248	47193
**South Batinah**	1335	29355
**Al-Dhahirah**	884	14386
**Al-Buraimi**	531	5939
**Musandam**	513	3216
**Al-Wusta**	302	2696
**Total**	**15077**	**260120**

1.3.1 Mapping and listing update

Two persons were appointed and trained to perform the mapping and listing operation for each governorate by using 2010 census maps from the National Centre for Statistics and Information. Personnel visited selected blocks to update maps that depicted all the households within the selected block and update the list of households within those blocks, including additional information (e.g. house number - Name of household head – nationality – language – description if needed).

1.4 Sampling Design

A multi-stage stratified cluster sampling was designed to select 9053 eligible subjects. Stratification was made on two factors: governorate and nationality (Omani and Non-Omani). The sample was drawn from the 2010 census block area (clusters). An ***equal size sample (cluster)*** was systematically randomly selected from each governorate. Selected clusters (blocks) had to be updated before the households were selected for survey (see previous section).

For peer review only - http://bmjopen.bmj.com/site/about/guidelines.xhtml

1.4.1 Rationale for preferring equal sample selection rather than proportional to population size

1. To get the desired precision and estimates of overall results and figures at the governorate level.
2. Proportional allocation would have resulted in a very small sample in a small governorate e.g. Al-Wusta with low precision in the estimate.
3. Equal sampling allowed high precision in individual stratum.
4. The experience from previous surveys in Oman. The lowest response rate was in Muscat governorate and one third of population live in Muscat so if proportional selection was used this would have directly affected the overall response rate.

For peer

In the first stage, all governorates in the Sultanate of Oman (11 governorates) were selected. In the second stage, we stratify blocks (PSU) based on geographical location, 550 clusters, from all over the Sultanate of Oman (50 from each governorate) using systematic random sampling (Secondary Sampling Units (SSU)), see Figure 2 of the Sultanate of Oman, by governorate.

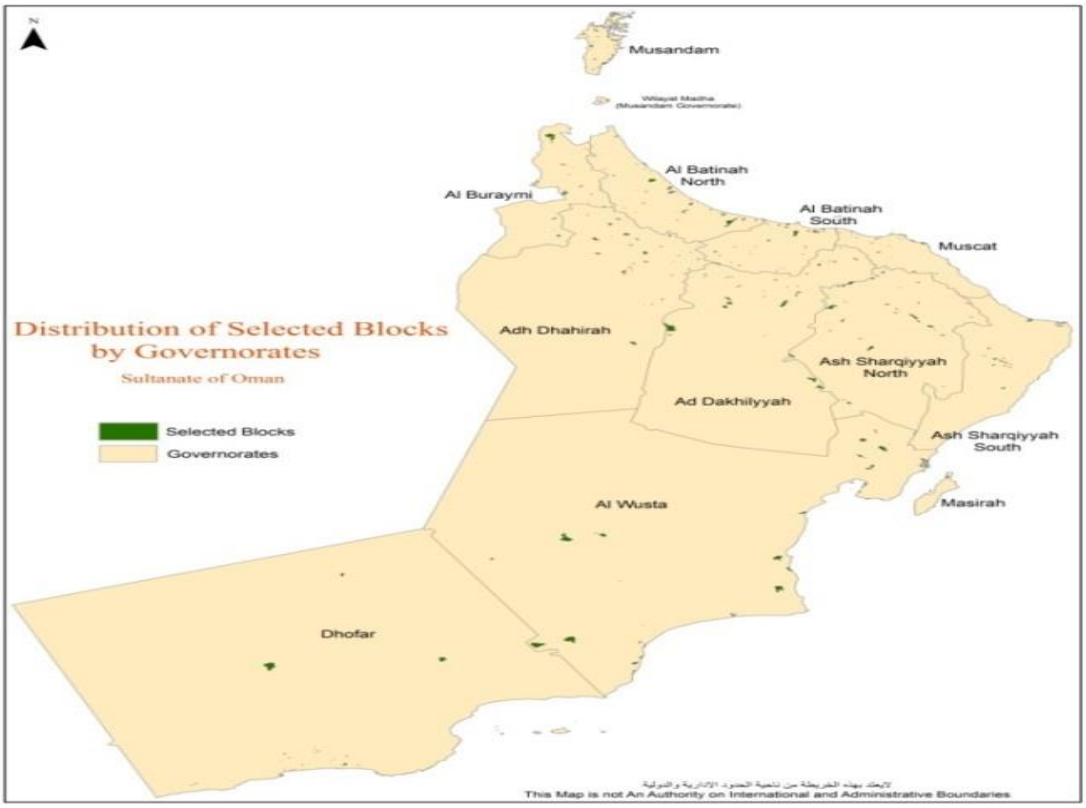

Figure 2: Distribution of Selected Blocks by governorate across the Sultanate of Oman
*Source: National Centre for Statistics and Information (NCSI)

For peer review only - http://bmjopen.bmj.com/site/about/guidelines.xhtml

In the third stage: [Tertiary Sampling Unit (TSU)], the following was considered:

i) The households in each cluster were listed within each governorate i.e. households from the first cluster, then households from the second cluster till households from the 50th cluster.

ii) The households in all clusters were aggregated into two lists by nationality (Omani and Non-Omani households) in each governorate

iii) 823 households were systematically and randomly selected from list of all households according to above in each governorate, from these two lists (Omani and Non-Omani household lists) according to the ratio of Omani and Non-Omani households in each governorate.

In the fourth stage: Ultimate sampling unit USU), One eligible individual from each household aged 18 years old or older was selected randomly by a program on the Android tablet.

1.4.2 Sample Size

A WHO STEPS standard formula was used in the calculation of the sample size based on the guidelines/recommendations of the STEPS survey.

$n= Z^2 * P (1-P)/d^2$

Where;

n= the required sample size

Z= the probability value associated with the confidence level

P= the prevalence rate of NCDs risk factors in the country

d= the desired margin of error (precision).

In turn:

Z= 1.96 (95% confidence interval as recommended)

P= 0.5 (the conservative value of prevalence rate)

e= 0.05 (as recommended in the guidelines)

Using these values, the initial calculation was: n= 384 households

Also taken into account for sample size calculations were: a value of design effect, as recommended in STEPS surveys, to be 1.5; and, an anticipated response rate of 70% was estimated. By adjusting the sample by these factors, the sample size per cluster (governorate) results in:

For peer review only - http://bmjopen.bmj.com/site/about/guidelines.xhtml

$$n = \frac{384\ x\ 1.5}{0.7} = 823 \text{ households}$$

To get the desired precision and overall figures adequate for age-sex groups and for overall estimate on governorates level, the sample size was:

n= 823 x 11 = 9053 households, with one individual selected per household

The 9053 households were distributed equally by governorate (823), proportional to nationality, according to the ratio of Omani and non-Omani households in each governorate as in Table 2.

Table 2: Oman STEPS survey sample size distributed, by governorate and nationality, 2017

Governorate	Selected no. of blocks in each governorate	Omani	Non-Omani	Total Households
**Muscat**	50	318	505	**823**
**Dhofar**	50	495	328	**823**
**Ad Dakhiliyah**	50	763	60	**823**
**North Sharqiyah**	50	520	303	**823**
**South Sharqiyah**	50	667	156	**823**
**North Batinah**	50	677	146	**823**
**South Batinah**	50	699	124	**823**
**Al-Dhahirah**	50	586	237	**823**
**Al-Buraimi**	50	722	101	**823**
**Musandam**	50	713	110	**823**
**Al-Wusta**	50	663	160	**823**
**Total**	**550**	**6823**	**2230**	**9053**

1.5 Data collection instruments and procedures used in the survey

1.5.1 Selection of participants

Upon selection of a household, all potential individuals for interview were identified and their age and gender recorded in a household list, subsequently used to determine selection probabilities and response rate. To randomly select an eligible individual from a household, the Kish method was used using an electronic tablet and software. Once individuals were selected, they were informed about the survey aims and asked to provide their consent to participate in the interview and subsequent measurement procedures.

1.5.2 Questionnaire (STEP 1)

An advanced standardized[2] country-specific version of the questionnaire, based on the Core and Expanded version of the WHO STEPS Instrument for Chronic Disease Risk Factor Surveillance[3] and country-specific requirements, contained 11 Core, 1 Optional and 4 country-

[2] validated

[3] Reference: The WHO STEPS Instrument for Chronic Disease Risk Factor Surveillance (1)

For peer review only - http://bmjopen.bmj.com/site/about/guidelines.xhtml

specific modules, to determine socio-demographic characteristics of participants, key behavioural risk factors (tobacco use, harmful alcohol consumption, diet with low fruits and vegetable intake, high salt intake, physical inactivity), given lifestyle advises, eye and ear health profile, history of chronic diseases, and health care coverage for diabetes, hypertension and dyslipidaemia, as well as cervical and breast screening.

In order to enhance the comparability with other countries in Arab region, the questionnaires from the Kuwait (2014), the Qatar (2012), the Bahrain (2007) and the Saudi Arabia (2005) STEPS surveys were taken into consideration. In addition, the questionnaire was translated from the original English version into Arabic as well as back translated, adapted to the local environment and needs, and piloted on 10 eligible respondents in terms of wording and understanding. The data collection was conducted in two languages, namely: Arabic and English.

Table 3: Questionnaire content by STEPS survey and county-specific modules and questions in the Oman survey, by type, 2017

	STEP and county-specific modules content	Questions		
**I**	**Core modules (11)**	**Core**	**Expanded**	**Country-specific**
1	Demographic information	4	5	3
2	Tobacco use	11	7	
3	Alcohol consumption	12		
4	Diet, including dietary salt	8	3	2
5	Physical activity	15	1	
6	History or raised blood pressure	5		1
7	History of diabetes	6		1
8	History of raised total cholesterol	5		1
9	History of cardiovascular disease	3		
10	Lifestyle advise	1		
11	Cancers (cervical cancer screening)	1		3
	Subtotal 1	71	16	11
**II**	**Optional Module (1)**			
1	Tobacco policy	7		8
	Subtotal 2	7		8
**III**	**Country-specific Modules (4)**			
1	Family history of chronic disease			8
2	Asthma			11
3	Eye health			3
4	Ear health			4
	Subtotal 3			26
**Grand Total: 139**		**78**	**16**	**45**

For peer review only - http://bmjopen.bmj.com/site/about/guidelines.xhtml

1.5.2.1 Demographic information

All eligible household members aged 18 and above were listed with one eligible member selected randomly to answer the demographic information questionnaire. Demographic information was assessed in terms of age, sex, marital status, educational status, employment status, and family income.

1.5.2.2 Tobacco use

Tobacco use was assessed in terms of current smoking (past 30 days), whether daily or non-daily, and former smoking or never smoking status, age of initiation and duration of smoking, type and quantity of tobacco use daily or weekly, smokeless tobacco use type and frequency, and exposure to second-hand smoke at home or workplace. Smoking cessation attempts, having received health professionals' advice, age and time since stopping to smoke, recognizing tobacco advertisement, promotion or sponsorship, all while applying 33 questions[4] were also asked. To facilitate recognition of types of tobacco use, data collectors used show cards, depicting types of commonly used tobacco products. Likewise, the above metrics were determined for users of smokeless tobacco products.

1.5.2.3 Alcohol consumption

Alcohol consumption was assessed using the concept of a standard drink. A standard drink is any drink containing about 10 g of pure alcohol. Accordingly, determinations have been made from different types of alcoholic beverages consumed, as follows: e.g. 30 ml of spirits, 120 ml of wine or 285 ml of beer. Again, data collectors used show cards depicting types of containers commonly used to consume alcoholic beverages as standard drinks, to determine consumption over 30 and 7 days prior to interview. Also, in an attempt to quantify and estimate total alcohol consumption, interviewers considered not only the most frequent but all types of alcohol consumed (e.g. wine, beer and spirits) and the amount of drinks on such occasions. Also included in the questionnaire were aspects about stopping alcohol consumption for health reasons or impacts.

Respondents who reported consuming alcohol within the past 30 days were classified as current drinkers, while those who identified absence of alcoholic beverages within previous 12 months as abstainers or ex-drinkers. Three risk categories were used to classify respondents who consumed alcohol according to the average amount of alcohol consumed per occasion. Furthermore, heavy ("binge") drinking patterns were determined according to largest number

[4] Based on STEPS and GATS questionnaires (2)

For peer review only - http://bmjopen.bmj.com/site/about/guidelines.xhtml

of drinks per drinking occasion and the percentage of those having consumed six or more standard drinks on one occasion during the past 7 days.

1.5.2.4 Diet

To assess the dietary patterns of the surveyed population, the respondents were asked about frequency of fruit and vegetable consumption, mean number of portions of these foods consumed daily and weekly, type of oils and fat used for meal preparation, number of meals eaten outside the household per week and the amount of salt added, and/or salty sauces used or processed food or consumed daily, using 13 questions.

Sufficient consumption of fruit and vegetables was assessed in terms of the number of servings and also compared to WHO recommended number of ≥ 5 servings/day and ≥ 5 day/week, with a serving being equal to 80 g. Show cards were used to facilitate the collection data on fruit and vegetable consumption on a typical day. Oil and fat intake show cards were also shown to assess about the type of oil or fat most frequently used for preparing food or cooking.

In turn, salt consumption was assessed by asking about frequency of addition of salt or a salty sauce to food during preparation, or before or while eating; and/or frequency of consumption of processed food high in salt. Participants were also asked about their perception of the quantity of salt they consumed and its link with health problems, as well as about the importance of reducing salt intake, and the measures undertaken to control it. WHO recommends a reduction to <2 g/day sodium (5 g/day salt) in adults (3).[5]

Population mean number of daily portions and of days per week consuming fruits and/or vegetables were calculated. Also, the percentage distribution of respondents according to their servings consumed per day and those meeting the WHO recommendation of fruits and vegetables intake/day were determined.

Regarding salt intake, the proportions of people reporting how often they added salt to foods before eating or when preparing foods, as well as those who think they eat too much salt were determined. Percentage of participants were further determined according to their belief on the importance of salt in diet and whether they thought their consuming to much salt can cause serious problems.

[5] http://www.who.int/nutrition/publications/guidelines/sodium_intake_printversion.pdf

For peer review only - http://bmjopen.bmj.com/site/about/guidelines.xhtml

1.5.2.5 Physical activity

Physical activity was assessed based on frequency, duration and intensity of physical activity at three segments: work (paid/ unpaid in and outside home), during transportation and on leisure time, for at least 10 minutes or more continuously per day, using a set of 16 questions[6]. Show cards were used to depict different types and places of physical activity.

The total time spent with diverse intensity (low, moderate, high or vigorous) was assessed using WHO recommended cut-off criteria (Table 4). According to WHO global recommendations on physical activity for (good) health, throughout a normal week adult should do at least the following amount of exercise (including activity for work, as well as during transport and leisure time): 150 minutes of moderate-intensity physical activity; or 75 minutes of vigorous-intensity physical activity; or an equivalent combination of moderate- and vigorous-intensity physical activity.

Table 4: Physical Activity Category for adults, by days/intensity/time

Physical activity category	Criteria, day/intensity/time
**High level**	Vigorous-intensity activity ≥ 3 days/week, attaining at least 75 minutes per week; or
**Moderate level**	Vigorous-intensity activity ≥ 2 days/week for at least 20 min a day; or<br>Moderate-intensity physical activity ≥ 5 days for at least 30 minutes a day;
**Low level**	Not meeting any of the above mention criteria

Mean and median minutes of physical activity per day according to place were computed; as a complement, time spent on sedentary activities on average per day was also calculated. The proportion of respondents not meeting the WHO recommendations was also calculated. Likewise, the proportion of participants according to levels of physical activity as recommended by WHO were determined.

1.5.3 Physical measurements (STEP 2)

1.5.3.1 Blood pressure

Resting blood pressure levels, both systolic (SBP) and diastolic (DBP), were measured using Omron M3 digital blood pressure device as recommended by WHO. The measurements were repeated three times and the three readings were recorded. In order to obtain the measurements

[6] Based on WHO Global Physical Activity Questionnaire (version 2) (4)

For peer review only - http://bmjopen.bmj.com/site/about/guidelines.xhtml

under relaxed conditions, persons were asked to void their bladder if needed, resting for 10-15 minutes after the interview, and not having drunk coffee before or during the measurement. In preparation for measurements, participants were asked to sit straight without crossing their legs. Blood pressure was measured placing a universal cuff on the left arm, which was placed with the palm face upward on a table surface at the level of the heart. While taking the readings participants were asked to remain silent. Repeat measurements were taken at 3-minute interval.

Participants were classified according to their blood pressure readings in the following categories: *normal* if their SBP and DBP readings were <140 mm Hg and <90 mmHg, respectively, and *high* if their SBP was ≥ 140 mm Hg and/or the DBP was ≥ 90 mm Hg, or if their readings were normal but they were under treatment for raised blood pressure in the past two weeks. In addition, *high risk* levels of SBP ≥ 160 mm Hg and/or DBP ≥ 100 mm Hg were also determined among participants to assess a higher probability or risk of hypertensive disorder.

Survey participants were also asked whether they were under medication for high blood pressure during the previous two weeks, as prescribed by a physician or other health professional. Respondents with treated and/or controlled raised blood pressure among those with raised blood pressure (SBP ≥140 and/or DBP ≥ 90 mmHg) or currently taking medication for raised blood pressure were further categorized to determine treatment success, treatment failure or being undetected and untreated, as follows:

- Under medication and controlled (treatment success) = those taking medication and having SBP <140 mmHg and DBP <90 mmHg;
- Under medication and uncontrolled (treatment failure) = those taking medication and having SBP ≥140 mmHg and/or DBP ≥90 mmHg;
- Undetected and uncontrolled (health system failure) = those not taking medication and having SBP ≥140 mmHg and/or DBP ≥90 mmHg**.**

1.5.3.2 Body mass index (BMI)

The height and weight of participants was taken to estimate their body mass index (BMI) as the ratio of weight/height^2 (at the nearest decimal kilogram and decimal centimetre, respectively).

Measurements were carried out while standing with the heels together, feet apart, arms at sides and chin parallel to the floor on a flat, horizontal and firm surface (like tile, cement or wooden floor). Standardized and calibrated SECA® 813 digital floor scales and 213 portable

For peer review only - http://bmjopen.bmj.com/site/about/guidelines.xhtml

stadiometers were used for weight and height measurements, respectively. To measure height and weight more precisely, participants were asked to follow standard procedures, including removal of their shoes and any bulky or heavy clothing to avoid overestimations.

Once BMI ratios determined, sample population was categorized according to the following WHO recommendations: underweight if BMI < 18.5, normal weight if BMI was between 18.5 - 24.9, overweight if BMI was between 25.0 - 29.9, and obesity if BMI was ≥ 30.

Average population BMI levels and proportion distribution among the sample population groups were determined.

1.5.3.3 Waist and hip circumferences

Waist circumference and hip circumference and their ratio were also assessed as other measures of obesity, in particular of central obesity.

Waist and hip circumference measurements were made while a participant remained standing, with feet together and hands on each side of the body, with a non-stretch Seca 201 measuring tape with millimetre precision. Waist circumference was measured by placing a tape measure around the bare abdomen at the midpoint between the lower margin of the last palpable rib and the top of iliac crest (hip bone). Hip circumference was measured by placing a tape measure around the maximum circumference over the buttocks.

The WHO cut-off points of waist circumference that determine waist obesity and categorized risk of metabolic complications and CVD are different for men and women. The waist–hip ratio (WHR) was computed among all respondents, excluding pregnant women. The WHO reference cut-off for WHRs was also used to define obesity as shown in Table 5.

Table 5: WHO cut-off points for waist circumference and waist-to-hip ratio, and their indication as a risk of metabolic complications

Measure indicator	Cut-off points (cm)		Risk of metabolic complications
	Men	Women	
**Waist circumference**	> 92	> 80	Increased
**Waist circumference**	> 102	> 88	Substantially increased
**Waist-hip ratio**	≥ 0.90	≥ 0.85	Substantially increased

For peer review only

1.5.3.4 Vision acuity

Visual acuity data of each eye were recorded ETDRS style folding tumbling "E" chart for reading at 4 meters (13 feet) was used for distance vision, for literate and illiterate persons.

Visual acuity was recoded after data collection and classified into the following categories:

- Blind: Defined as visual acuity of LOG value 2 (visual acuity is < 3/60 or less) in the better eye after correction.
- Severe visual impairment: Defined as vision less than LOG value of 1 (6/60) in better eye after correction.
- Low vision: Defined as vision less than LOG value of 0.5 and vision ≥ 1 (visual acuity is < 6/18 and > 6/60) in the better eye.
- Adequate vision: Defined as vision of LOG >0.5 (visual acuity is > 6/18) in the better eye.

The proportions of participants in the sample population were determined according to visual acuity categories.

1.5.4 Biochemical markers (STEP 3)

Biomarker levels among survey respondents were determined to assess raised blood glucose and cholesterol, high density cholesterol, and triglycerides, as indicators of risk of hyperglycaemia and diabetes and cardio-metabolic risk. Urinary salt, creatinine and potassium excretion were measured to estimate sodium intake and identify high risk of hypertensive disease, and renal (including diabetic) disease. To limit loss to follow-up, blood samples were collected within two days after completing STEPS 1 and 2.

Blood glucose and lipid profile were measured using portable dry chemistry (CardioChek® Plus Analyzer) instrument as recommended by the WHO. Blood samples were taken after at least 12 hours of fasting by trained nurses in each team. The fingertip was utilized to facilitate withdrawing the blood. The recorded results were registered in the Android tablet according to the personal identification number.

Spot urine samples were collected as part of a STEPS survey and participants were asked to collect their urine in the evening before fasting, and take it with them to the appointment for blood testing the next morning.

The "gold-standard" approach to assessing population salt intake is to obtain urine samples collected over 24 hours (to avoid diurnal variations) on a representative sample of the population. A trained research assistant/examiner was provided verbal instructions on how to collect and preserve the urine sample, where to deliver the completed sample or how the sample

For peer review only - http://bmjopen.bmj.com/site/about/guidelines.xhtml

was picked up from the participant kit including, written instructions, carry bag and 4-5L urine sample bottle/container labelled with the participants' ID number and gender was provided.

Mean population blood and urinary biomarkers measurements were calculated, while the proportions of sample population categorized by their biomarkers WHO cut-off points were defined, according to Table 6.

Table 6: Biochemical blood and urine indicators cut-off points as recommended by WHO

Biochemical indicator	Normal	At risk	Increased
**Glucose**	< 5.6 mmol/L	≥ 5.6 mmol/L to 6.1 mmol/L	≥ 6.1 mmol/L or using glucose-lowering drugs
**Cholesterol**	< 5.0 mmol/L	≥ 5.0 mmol/L to 6.1 mmol/L	≥ 6.2 mmol/L or using cholesterol-lowering drugs
**HDL cholesterol**			
Men	< 1.03 mmol/L	≥ 1.03 mmol/L and	
Women	< 1.29 mmol/L	≥ 1.3 mmol/L	
**Triglycerides**	< 2.0 mmol/L	≥ 2.0 mmol/L	
**Urine sodium excretion**	< 5 g/day	> 5 g/day	

Source: WHO, 2012 (3)

1.6 Data collection

1.6.1 Staff recruitment

Interviewers and field supervisors in each governorate were recruited from among staff working in the Ministry of Health.

Overall, 66 data collectors and 11 field supervisors were recruited to participate in the training of data collection along with 1 regional coordinator, 1 IT technician and 1 laboratory technician in each governorate. Data collectors were mainly nurses and health educators nominated for data collection and for measurement of height, weight, waist and hip circumference, blood pressure, blood glucose, lipid profile, and vision testing. Urine samples were measured by the laboratory technicians in the health centre laboratories.

1.6.2 Data collection procedures

Field operations were carried out in the governorates during a four-month period in 2017, with the survey period chosen appropriately to avoid Ramadan/Eid periods. A media and advocacy action plan was implemented to raise awareness of the population about the survey, including disseminating information through leaflets, posters, press releases, radio and TV broadcasting, community and local Ministry of Health (MoH) staff participation. In addition to this focused publicity campaign, official identity cards to the field staff issued by MoH were of great help to secure sufficient recognition, cooperation and good responses for the interview in most

For peer review only - http://bmjopen.bmj.com/site/about/guidelines.xhtml

cases. The respective authorities were also requested to provide the necessary assistance and co-operation to the field staff.

The interviews were conducted in all the governorates. The supervisors approached the selected households in each cluster, explained the aim and objectives of the survey, and sought their consent to participate in the survey. After recording the eligible members within a selected household, one participant was randomly selected from eligible members by the Android tablet. Each interview took place in a secure setting with adequate privacy at the household level. Each participant was interviewed at his/her household. As biomedical tests require 12 hours of fasting, appointments were given based on agreement between the interviewers and the respondents. Interviewers also explained the protocol for 24-hour urine collection to the respondent, obtained informed consent and provided the record sheet on which participants note the start and finish times of their 24-hour urine collection and any missed urine collections in the container.

1.6.3 Training for supervisors and data collectors

A one-week training program was conducted by the central research team from the Centre of Studies & Research in collaboration with WHO experts. This training program, which was held in Muscat in December 2016, included the survey objectives and field work staff duties, how to fill in the questionnaire in a polite, motivating and persuasive manner, how to understand the content of the questions if needed to clarify to respondents, how to enter the data and navigate the Android tablet, how to ask for written consent and organize data collection and protection of confidentiality of the informant (consent, ID barcode labels, patterns of verbal and nonverbal behaviour), how to perform physical measurements and take biomarkers' samples using equipment applying standardized methodology, and role playing and mock interviews.

The field supervisors were given special instructions afterwards on coordinating process. Trainees who failed to show interest in the survey and those who did not attend the training program on a regular basis were not selected for the fieldwork and were replaced by other staff from the same Governorate after training.

1.6.4 Pilot Study

The pilot testing of the survey implementation process was implemented for 2 days (using Android tablets) selecting about 100 households which included males and females as well as Omani and non-Omani households. The pilot was also used to train the key survey personnel, test all survey materials prior to full implementation (skip errors, translation errors, awkward

For peer review only - http://bmjopen.bmj.com/site/about/guidelines.xhtml

wording, and inadequate response categories), check quality of data collected. Lessons learned, logistic issues, and challenges identified were considered which maximized opportunities for improving the quality in the full survey implementation.

1.7 Data Management

1.7.1 Data collection

Each participant was allocated an identifier code (PID). The PID code consisted of seven digits the first two digits were the device number; the next three digits were the house listing number in the cluster and the last two digits were the person number in the household.

Data from STEP 1 and STEP 2 were submitted electronically online to the server from a household with identification of individual PID, household geographical location, including cluster with individual PID, day and time of completion. This was done either daily or at least once a week. After being analysed, blood and urine samples' results (STEP 3) were uploaded to the server and merged into the unified dataset, following conversion into SPSS and Microsoft Excel format in a single file.

1.7.2 Data validation

The central team (at the Centre of Studies & Research) downloaded data daily from server for data cleaning and management over a period of 6 months. Data management included continuously monitoring data collection, uploading and consolidation processes in the field, validating quality of the data, creating weights, removing inconsistencies, namely "jump" errors/outliers, absence of data, excess data and invalid data. Moreover, to increase reliability of the collected data, verification of data in field was organized among 500 randomly selected households from all governorates. Accuracy of recording categorical and continued variables was checked using range and logic functions. The team also provided advice on software support and reported any problems or interview errors to the data collection field supervisors.

1.7.3 Data analysis

Weightage and Adjustment for sampling variation

Survey data analyses have to take into account whether the results are representative of the sample alone (unweighted analysis) or of the entire target population (weighted analysis). Since the primary objective was to be able to determine the estimates for the whole country, a weighted analysis was considered necessary. Weights adjusting for this complex survey design were required to decrease the risk of biases resulting from diverse factors. The sample weight

For peer review only - http://bmjopen.bmj.com/site/about/guidelines.xhtml

is comprised of the inverse probability of selection. The household weights took into account the selection probability of the clusters within each stratum and the size (the number of households) of the cluster. The sample weight was also adjusted for non-response at the household level. The individual weight assumed that adults in the same cluster were selected by simple random sampling but the calculation scheme did not take into account the household size. This approach could have biased any key indicators, which was strongly associated with the household size. The individual weight was also adjusted for non-response.

Means, medians, proportions, standard errors, and 95% confidence intervals (95% CI) values were calculated to estimate central and dispersion measures and used to assess prevalence differences of NCD risk factors. Statistical procedures for data calculation and analyses were performed through two programs: EpiInfo in collaboration with WHO, and IBM SPSS (Version 20). All the figures and indicators in the tables were calculated using SPSS complex samples analysis. The figures presented as footnote (with an asterism) under each table were calculated after using population proportion weight. To allow for international comparisons of Oman survey results, age- and sex-adjusted overall values were calculated for all indicators using the direct method and the WHO standard population. Values are presented as footnotes on the tables to limit confusion with the national unadjusted data. It should also be noted that the estimates shown for governorates in the tables should be treated with caution as they represent the respondents in the respective governorate, and not the governorate itself.

1.8 Ethical considerations

Two informed consent forms, one for filling in the questionnaire and performing physical measurements (e.g. STEP 1 and STEP 2) and another for taking blood and urine samples for biomarkers (STEP 3), were requested to be signed by each participant. To enhance participation, an information letter was sent to all selected households in advance of data collection, identifying purpose, benefits and the voluntary participation in the survey.

To guarantee the high level of confidentiality and data security, every eligible subject was granted a unique identification number which was used for any reference from the register, with the exception of providing a personal feedback to a particular eligible subject for medical reasons.

Prior to its implementation, the survey was approved by the Research and Ethical Review & Approval Committee (RERAC) of the Ministry of Health.

For peer review only - http://bmjopen.bmj.com/site/about/guidelines.xhtml

STROBE Statement—Checklist of items that should be included in reports of ***cross-sectional studies***

	Item No	Recommendation	Page No
**Title and abstract**	1	(*a*) Indicate the study's design with a commonly used term in the title or the abstract	1,2
		(*b*) Provide in the abstract an informative and balanced summary of what was done and what was found	2
**Introduction**			
Background/rationale	2	Explain the scientific background and rationale for the investigation being reported	4
Objectives	3	State specific objectives, including any prespecified hypotheses	5
**Methods**			
Study design	4	Present key elements of study design early in the paper	5-6
Setting	5	Describe the setting, locations, and relevant dates, including periods of recruitment, exposure, follow-up, and data collection	5-7
Participants	6	(*a*) Give the eligibility criteria, and the sources and methods of selection of participants	5-7
Variables	7	Clearly define all outcomes, exposures, predictors, potential confounders, and effect modifiers. Give diagnostic criteria, if applicable	5-7
Data sources/ measurement	8*	For each variable of interest, give sources of data and details of methods of assessment (measurement). Describe comparability of assessment methods if there is more than one group	5-7
Bias	9	Describe any efforts to address potential sources of bias	5-7
Study size	10	Explain how the study size was arrived at	7
Quantitative variables	11	Explain how quantitative variables were handled in the analyses. If applicable, describe which groupings were chosen and why	7
Statistical methods	12	(*a*) Describe all statistical methods, including those used to control for confounding	7
		(*b*) Describe any methods used to examine subgroups and interactions	N/A
		(*c*) Explain how missing data were addressed	7
		(*d*) If applicable, describe analytical methods taking account of sampling strategy	7
		(*e*) Describe any sensitivity analyses	Suppl Material
**Results**			
Participants	13*	(a) Report numbers of individuals at each stage of study—eg numbers potentially eligible, examined for eligibility, confirmed eligible, included in the study, completing follow-up, and analysed	8-9
		(b) Give reasons for non-participation at each stage	8-9
		(c) Consider use of a flow diagram	Figure 2
Descriptive data	14*	(a) Give characteristics of study participants (eg demographic, clinical, social) and information on exposures and potential confounders	8, Tab 1, 2

For peer review only

For peer review only - http://bmjopen.bmj.com/site/about/guidelines.xhtml

		(b) Indicate number of participants with missing data for each variable of interest	Suppl.material
Outcome data	15*	Report numbers of outcome events or summary measures	Tab 2, 3, 4, 5
Main results	16	(*a*) Give unadjusted estimates and, if applicable, confounder-adjusted estimates and their precision (eg, 95% confidence interval). Make clear which confounders were adjusted for and why they were included	7-9<br>Tab 2-5
		(*b*) Report category boundaries when continuous variables were categorized	Tab 2-3
		(*c*) If relevant, consider translating estimates of relative risk into absolute risk for a meaningful time period	N/A
Other analyses	17	Report other analyses done—eg analyses of subgroups and interactions, and sensitivity analyses	Suppl. material
**Discussion**			
Key results	18	Summarise key results with reference to study objectives	9
Limitations	19	Discuss limitations of the study, taking into account sources of potential bias or imprecision. Discuss both direction and magnitude of any potential bias	10-11
Interpretation	20	Give a cautious overall interpretation of results considering objectives, limitations, multiplicity of analyses, results from similar studies, and other relevant evidence	10-12
Generalisability	21	Discuss the generalisability (external validity) of the study results	10-12
**Other information**			
Funding	22	Give the source of funding and the role of the funders for the present study and, if applicable, for the original study on which the present article is based	3

*Give information separately for exposed and unexposed groups.

Note: An Explanation and Elaboration article discusses each checklist item and gives methodological background and published examples of transparent reporting. The STROBE checklist is best used in conjunction with this article (freely available on the Web sites of PLoS Medicine at http://www.plosmedicine.org/, Annals of Internal Medicine at http://www.annals.org/, and Epidemiology at http://www.epidem.com/). Information on the STROBE Initiative is available at www.strobe-statement.org.

For peer review only - http://bmjopen.bmj.com/site/about/guidelines.xhtml

BMJ Open

A national survey to estimate sodium and potassium intake, and knowledge attitudes and behaviours towards salt consumption of adults in the Sultanate of Oman.

Journal:	*BMJ Open*
Manuscript ID	bmjopen-2020-037012.R1
Article Type:	Original research
Date Submitted by the Author:	03-Jun-2020
Complete List of Authors:	Al-Mawali, Adhra; Ministry of Health Oman, Centre of Studies & Research<br>D'Elia, L; Federico II University Hospital, Department of Clinical Medicine and Surgery<br>Jayapal, Sathish; Ministry of Health Oman, Centre of Studies & Research<br>Morsi, Magdi; Ministry of Health Oman, Centre of Studies & Research<br>Al-Shekaili, Waleed ; Ministry of Health Oman, Centre of Studies & Research<br>Pinto, Avinash; Ministry of Health Oman, Centre of Studies & Research<br>Al-Kharusi, Hilal ; Ministry of Health Oman, Centre of Studies & Research<br>Al-Balushi, Zainab; Ministry of Health Oman, Centre of Studies & Research<br>Idikula, John; Ministry of Health Oman, Centre of Studies & Research<br>Al Harrasi, Ayaman; Ministry of Health Oman, Centre of Studies & Research<br>Cappuccio, Francesco Paolo; University of Warwick Warwick Medical School, Division of Health Sciences
**Primary Subject Heading**:	Epidemiology
Secondary Subject Heading:	Nutrition and metabolism, Public health
Keywords:	EPIDEMIOLOGY, NUTRITION & DIETETICS, PUBLIC HEALTH

SCHOLARONE™
Manuscripts

BMJ

I, the Submitting Author has the right to grant and does grant on behalf of all authors of the Work (as defined in the below author licence), an exclusive licence and/or a non-exclusive licence for contributions from authors who are: i) UK Crown employees; ii) where BMJ has agreed a CC-BY licence shall apply, and/or iii) in accordance with the terms applicable for US Federal Government officers or employees acting as part of their official duties; on a worldwide, perpetual, irrevocable, royalty-free basis to BMJ Publishing Group Ltd ("BMJ") its licensees and where the relevant Journal is co-owned by BMJ to the co-owners of the Journal, to publish the Work in this journal and any other BMJ products and to exploit all rights, as set out in our licence.

The Submitting Author accepts and understands that any supply made under these terms is made by BMJ to the Submitting Author unless you are acting as an employee on behalf of your employer or a postgraduate student of an affiliated institution which is paying any applicable article publishing charge ("APC") for Open Access articles. Where the Submitting Author wishes to make the Work available on an Open Access basis (and intends to pay the relevant APC), the terms of reuse of such Open Access shall be governed by a Creative Commons licence – details of these licences and which Creative Commons licence will apply to this Work are set out in our licence referred to above.

Other than as permitted in any relevant BMJ Author's Self Archiving Policies, I confirm this Work has not been accepted for publication elsewhere, is not being considered for publication elsewhere and does not duplicate material already published. I confirm all authors consent to publication of this Work and authorise the granting of this licence.

For peer review only

Tuesday, 02 Jun 2020 – v11- R2

A national survey to estimate sodium and potassium intake, and knowledge attitudes and behaviours towards salt consumption of adults in the Sultanate of Oman.

Adhra Al-Mawali[1,2], Lanfranco D'Elia[3,4], Sathish Kumar Jayapal[1], Magdi Morsi[1], Waleed Nasser Al-Shekaili[1], Avinash D Pinto[1], Hilal Al-Kharusi[1], Zainab Al-Balushi[1], John Idikula[1], Ayaman Al-Harrasi[1] and Francesco P Cappuccio[3,5,6].

[1]Centre of Studies & Research, Ministry of Health, Muscat, Sultanate of Oman
[2] The Research Council, Sultanate of Oman
[3]WHO Collaborating Centre for Nutrition, University of Warwick, Coventry, UK
[4]Department of Clinical Medicine and Surgery, "Federico II" University of Naples Medical School, Naples, Italy.
[5]Division of Health Sciences, Warwick Medical School, University of Warwick, Coventry, UK
[6]University Hospitals Coventry & Warwickshire NHS Trust, Coventry, UK

Keywords: Sultanate of Oman; salt; sodium; potassium; population.
Short title: Sodium and potassium intake in Oman
Word count: 4,182

Correspondence to:

Prof. Francesco P Cappuccio
WHO Collaborating Centre for Nutrition
University of Warwick, Warwick Medical School, Division of Health Sciences
Gibbet Hill Road, Coventry CV4 7AL, UK
f.p.cappuccio@warwick.ac.uk; Tel.: +442476573129

For peer review only - http://bmjopen.bmj.com/site/about/guidelines.xhtml

Tuesday, 02 Jun 2020 – v11- R2

ABSTRACT

Objectives. To estimate population sodium and potassium intakes, and explore knowledge, attitudes and behaviour (KAB) towards the use of salt in adults in the Sultanate of Oman.

Design. National cross-sectional population-based survey.

Setting. Proportional random samples, representative of Omani adults (18 years or older), were obtained from all Governorates of the Sultanate of Oman.

Participants. Five hundred and sixty-nine (193 men, 376 women; 18 years or older) were included in the analysis (response rate 57%). Mean age was 39.4 years (SD 13.1). Participants attended a screening including demographic, anthropometric and physical measurements.

Primary and secondary outcome measures. We assessed dietary sodium, potassium and creatinine by 24h urinary sodium (UNa), potassium (UK) and creatinine (UCr) excretions. We collected KAB by a questionnaire on an electronic tablet.

Results. Mean UNa was 144.3 (78.8) mmoL/day, equivalent to 9.0 g of salt/day and potassium excretion 52.6 (32.6) mmoL/day, equivalent to 2.36 g/day, after adjusting for non-urinary losses. Men ate significantly more sodium and potassium than women. Only 22% of the sample had a salt intake below the World Health Organization (WHO) recommended target of 5 g/day and less than 10% met WHO targets for potassium excretion (>90 mmoL/day). Whilst 89.1% of those interviewed knew that consuming too much salt could cause serious health problems and only 6.9% felt they were using too much added salt, 1 in 2 participants used always or often salt, salty seasonings or salty sauces in cooking or when preparing food at home.

Conclusions. In the Sultanate of Oman, salt consumption is higher, and potassium consumption lower, than recommended by WHO, both in men and in women. The present data provides, for the first time, evidence to support a national programme of population salt reduction to prevent the increasing burden of CVD in the area.

Tuesday, 02 Jun 2020 – v11- R2

Article summary

Strengths and Limitations of the study

- National survey of Omani men and women using 24h urine collections.
- Adoption of quality control process to minimize the use of incomplete urine collections.
- Overall response rate was 57%, comparable with other similar population surveys.
- Non-responders did not differ in their baseline characteristics from responders.
- We cannot rule out the risk of selection bias.

Funding statement

The Ministry of Health of the Sultanate of Oman and the EMRO Regional Office of the World Health Organization supported the study.

Competing interests

AA-M, SKJ, MM, WNA-S, ADP, HA-K, ZA-B, JI, AA-H are all staff of the Ministry of Health of the Sultanate of Oman. FPC is a technical advisor to the World Health Organization, unpaid member of Action on Salt and WASH. LD was a technical advisor to the World Health Organization and is a member of the Scientific Committee of the Italian Society of Human Nutrition.

Tuesday, 02 Jun 2020 – v11- R2

INTRODUCTION

Non-communicable diseases (NCDs) are the leading, yet preventable, causes of death worldwide[1]. The reduction of its burden is now a global health priority of the United Nations[2], endorsed by the World Health Organization (WHO) Action Plan that has identified a set of cost-effective policy options ('best buys'), of which reduction in population salt consumption is one[3].

In the Sultanate of Oman, NCDs are amongst the leading causes of death, accounting for 72% of all deaths[4]. Cardiovascular disease (CVD) represents an increasingly common cause of population morbidity and mortality, accounting for 36% of all deaths[4]. It represents a major public health challenge undermining socio-economic development[5].

High blood pressure (BP) and unhealthy diets are the leading risk factors for CVD in the world[1]. Raised BP is a determinant of the CVD risk in the Sultanate of Oman, where the prevalence of raised blood pressure in people aged 18 years or older is 33%, higher in men (39%) than in women (27%)[5-6].

High salt (i.e., sodium chloride, 1 g = 17.1 mmoL of sodium) consumption is an important determinant of high BP. A high salt intake is associated with raised BP that leads to increased risk of vascular diseases[7-10]. In addition, high salt intake is related to adverse health effects independent of its effects on BP[11-13]. A moderate reduction in salt consumption reduces BP[7-8] and it can improve the health outcomes and indirectly reduce the overall mortality through beneficial effect on the BP[9-10].

The World Health Organization (WHO) recommends that adults should consume no more than 5 g of salt daily[14]. However, mean daily intakes of salt in most of the countries in the world exceed this recommendation[15-16]. Whilst there is no definitive estimate of population dietary salt intake in the Sultanate of Oman, average consumption could be high, similar to some countries in the sub-region[17-18]. In the Sultanate of Oman it is a common habit to add salt and salty condiments to food at the table and whilst cooking. Also, the habit of eating out is increasing (especially in urban areas) which leads to an increased salt intake, since restaurants tend to use higher amounts of salt to render food tastier. Our study was designed to support the salt reduction strategy of the Eastern Mediterranean region, including the Sultanate of Oman, in which monitoring population salt consumption is one of the three pillars[19]. Current national initiatives include establishment of a multi-sectoral national committee, legislation on salt reduction, development of salt content benchmarks, dietary guidelines[18]. The "Health Vision 2050" for the Sultanate of Oman was also developed as a roadmap by analysing extensively the status of the Omani health system, the morbidity and mortality in the population, the challenges facing the health

For peer review only - http://bmjopen.bmj.com/site/about/guidelines.xhtml

system, the expected future developments and changes in the population including macro-social and macro-economic changes in order to augment the performance of the health system.

In contrast to sodium, epidemiological and intervention studies suggest beneficial effects of dietary potassium on BP and cardiovascular health[20-22]. The Sultanate of Oman lacks data on actual potassium consumption. The WHO currently recommends that adults should consume not less than 90 mmoL of potassium daily[23]. Hence, we need reliable data on sodium and potassium intake in the Sultanate of Oman.

The primary aim of the present study was to establish current baseline average consumption of sodium and potassium by 24h urine collection, in a national random sample of Omani men and women. The study also aimed to explore knowledge, attitudes and behaviour towards dietary salt.

MATERIAL AND METHODS

Participants and Recruitment

We nested the salt survey within the main Oman NCD survey of 6,833 households (Supplementary material, Text S1). We recruited only one member per household. We designed the salt survey to collect 24h urinary samples from a subgroup of at least 90 participants from each governorate (region). The survey included Omani citizens only. We included a total of 999 randomly selected Omani men and women. They were all aged 18 years or older. They comprised residents of all governorates of the Sultanate of Oman (Table 1). The sample was representative of the national sample for its general characteristics (Supplementary material, Table S1).

From the sampling frame and according to the EMRO-WHO Protocol[24], we excluded the following groups: people unable to provide informed consent, those with known history of heart or kidney failure, stroke, liver disease, those who recently began therapy with diuretics (less than two weeks), pregnant women, any other conditions that would make 24h urine collection difficult. To detect approximately 1 g reduction in salt intake over time using 24h urinary sodium excretion (difference ~20 mmoL/24h), with a standard deviation of 75 mmoL/day (alpha = 0.05, power = 0.80), a minimum sample of 120 individuals per stratum is recommended[24]. Thus, we estimated a minimum recommended sample size of 240 per age and sex groups and adjusted for an anticipated non-response rate of 50%[24]. We stratified the population in groups by sex (men and women). Therefore 480 individuals were originally needed to be selected (total n=120*2 groups/0.5 attrition=480).

For peer review only - http://bmjopen.bmj.com/site/about/guidelines.xhtml

The survey took place between December 2017 and May 2018. From the 999 individuals interviewed in the sampling frame, 569 of them (57.0%) provided suitable data for inclusion in the survey analysis. The general characteristics of the included participants did not differ substantially from those of the excluded participants (Supplementary material, Table S2). Originally, 262 (26.0%) did not provide complete urine collections (either declaring missing more than one void or providing collections <23h or above 25h), 87 (8.7%) had missing data, 48 (4.8%) provided urine collections with volume less than 500 mL (conventionally taken as not plausible), and 24 (2.4%) had urinary creatinine excretion outside 2 standard deviations (SDs) of the sex-specific distribution of urinary creatinine in the sample (Figure 1).

Ethical considerations

We carried out the survey in accordance with the Declaration of Helsinki and Good Clinical Practice[25]. We obtained ethical approval for the survey from the Research and Ethics Review and Approval Committee (RERAC) of the Ministry of Health of the Sultanate of Oman and participants provided written informed consent to take part.

Patient and Public Involvement

No patient involved

Data Collection

We performed the examination in a quiet and comfortable room, with the participants who did not smoke, exercise, eat, and consume caffeine before attending, and had a full bladder for 30 minutes before measurements. We carried out the survey in three steps: a) questionnaire survey, b) physical measurements and c) 24h urine collections.

We based the questionnaire on the Core and Expanded version of the WHO STEPS Instrument for Chronic Disease Risk Factor Surveillance (version 3.0)[26] and country-specific requirements. It contained 11 Core, 1 Optional and 4 country-specific modules which included a total of 420 questions, to determine socio-demographic characteristics of participants, key behavioural risk factors (tobacco use, harmful alcohol consumption, diet with frequency of fruit and vegetable consumption, high dietary salt consumption, oil and fat use, physical inactivity), knowledge attitudes and behaviour on dietary salt, given lifestyle advises, and additional health-related information not presented here. Knowledge, attitude and behaviours toward the consumption of salt was assessed by asking participants about the frequency, quantity, and type of salt used in the household, as well as their cooking habits and their attitudes and perceptions towards dietary salt intake. Processed food was defined, per WHO STEPS protocol, as foods altered from their natural state, such as

For peer review only - http://bmjopen.bmj.com/site/about/guidelines.xhtml

packaged salty snacks, canned salty food, cheese, and processed meat along with country-specific pictorial show cards.

We measured anthropometric indices, BP and heart rate in all participants. Height was in cm and body weight in kg using a Standardized and calibrated SECA®813 digital floor scales and 213 portable stadiometers, respectively. Body mass index (BMI) was calculated as weight (kg) divided by height squared (m^2). Waist and hip circumferences were measured by a non-stretch SECA®201 measuring tape to the nearest mm[24]. We took systolic and diastolic BP and heart rate measurements three times in the right arm on a sitting position, using an appropriate cuff and a validated digital device (OMRON M3). We ignored the first measurement, and used the mean of second and third measurements for analysis. We took measurements after the participant had rested for 15 minutes and each with three minutes of rest between the measurements (maximum deviation of cuff pressure measurement ± 3 mmHg and of pulse rate display ± 5%). Hypertension is defined as systolic and/or diastolic BP ≥ 140/90 mmHg or regular antihypertensive treatment[27]. We obtained a single 24h urine collection from the participants. We gave each participant a leaflet with explanations along with the necessary equipment and a record sheet on which participant noted the start and the finish times of their urine collection, any missed urine aliquots and any medication taken during the collection. We instructed the participants carefully on urine collection methodology[24]. In an effort to minimize bias, we also requested participants not to change their diet before or during the day of the urine collection. They discarded the first void upon waking on the day of collection. Participants then filled the 24-hour urine container over the 24-hour period. On the following day, the field team-members visited the household, measured total volume, mixed it thoroughly, and obtained a urine sample, which was kept in a cool-box for transport to the respective laboratory. On arrival at the laboratory, we either carried out sodium, potassium and creatinine determinations immediately or stored samples in the fridge until the determination (as soon as possible). Sodium and potassium concentration in the urine samples were determined using an Ion Selective Electrode with an Abott C8000 & Roche Cobas 6000 and expressed in mmol/L[28]. Creatinine concentration was determined through either the kinetic (Abbott C8000) or enzymatic (Roche Cobas 6000) method and expressed in mg/dL[29]. These determinations were carried out in one reference laboratory in each of the 11 governorates, except for 2 regions (Dhofar and Musandam) which had 2 receiving reference laboratories each. All laboratories underwent joint quality control.

For peer review only - http://bmjopen.bmj.com/site/about/guidelines.xhtml

Statistical Analysis

We performed all statistical analyses using the SPSS software, version 20 (SPSS Inc., Chicago, IL, USA). We used T-test for unpaired samples to assess differences between group means and Pearson chi-square test to test the association between categorical variables. To convert urinary output into dietary intake, we first converted the urinary excretion of sodium (UNa) or potassium (UK) values (mmoL/day) into mg/day (for sodium 1 mmol = 23 mg of sodium, for potassium 1 mmol = 39 mg). We then multiplied the sodium value by 2.542 to convert dietary sodium (Na) intake into salt (NaCl) intake. We finally multiplied sodium values by 1.05 (assuming that approximately 95% of sodium ingested is excreted)[30]. We calculated potassium dietary intake assuming that 85% of the potassium ingested is excreted in the urine[31]. The results were reported as mean (SD), median (IQ range) or as percentages, as appropriate. We considered two-sided p below 0.05 as statistically significant.

RESULTS

The final population sample included 569 participants between 18 and 69 years old (n = 193 or 34% men and n = 376 or 66% women), recruited nationally.

Characteristics of the Participants

Table 2 shows the characteristics of the participants. There was no statistically significant difference in the mean age and in body mass index between men and women, however men had significantly higher BP and slower heart rate than women did. The prevalence of hypertension was on average 27.4%, significantly higher in men than in women (38.5% v 21.7%, p<0.001).

Daily Urinary Excretions of Volume, Sodium, Potassium and Creatinine and Salt and Potassium Intake

Average urinary volume excretion was 1354 mL per day, being higher in men than women (Table 3). Average urinary creatinine excretion was 1.33 g per day, being again higher in men than women (Table 3). Mean urinary sodium was 144.3 (SD 78.8) mmoL/24h, equivalent to a mean consumption of 9.0 (4.9) g of salt per day (Table 3). Men excreted more sodium than women did (mean difference 15.0 mmoL/24h, p < 0.05), equivalent to ~1.0 g of higher salt consumption than women did. Only 22% of the participants met the levels of salt intake of 5 g or less recommended by the WHO, with no difference between sexes. Mean urinary potassium was 52.6 (32.6), equivalent to a mean consumption of 2.36 (1.46) g of potassium per day (Table 3).

For peer review only - http://bmjopen.bmj.com/site/about/guidelines.xhtml

Men excreted significantly more potassium than women ; 9.1% of participants met the levels of potassium intake of 90 mmoL/day or more recommended by the WHO.

The sex difference in total daily salt and potassium intakes is almost entirely due to the fact that men eat more food than women, as they are taller and heavier, despite having comparable body mass index. This is a consistent finding across populations in different countries and from different continents.

Knowledge, Attitude, and Behaviours towards Salt Intake and other eating patterns.
Knowledge, attitude, and behaviours toward the consumption of salt are presented in Table 4. A total of 28.1% of respondents mentioned that they added salt or salty sauces always or often to food. The percentage of women who added salt or salty sauces always or often to their meal was significantly higher than that of men (30.8% vs 22.8%; p=0.005). A total of 47.0% of respondents reported that they always or often added salt, salty seasonings or sauces when cooking or preparing food at home, women more than men (48.1% vs 44.8%; p=0.04). More than 1 in 5 (22.3%) mentioned that they consumed processed foods high in salt always or often. Very few (6.9%), however, felt they consumed too much salt or salty sauces, although 89.1% knew that consuming too much salt could cause serious health problems. We also asked participants about dietary attitudes about the consumption of fruit and vegetables, oil or fats (Table 5). Interestingly, 68.0% consumed fruit at least five days a week, and 54.5% at least three servings on these days. Men appeared to report more fruit consumption than women did (40.4% vs 27.7%; p=0.002). Vegetables were also consumed frequently (75.9% at least 5 days a week), with 40.1% having at least 3 servings on one of those days (women more frequently than men). The majority (90.8%) used vegetable oil for meal preparation in the household and more than half (54.2%) consumed food prepared outside home at least once a week. Men were more likely than women to do so (67.9% vs 47.2%; p<0.001).

DISCUSSION

This the first nationally representative population-based survey carried out in the Sultanate of Oman assessing dietary sodium and potassium consumption in adult Omani men and women, using the gold standard measure of 24h urinary sodium and potassium excretions as a biomarker of intake. The results show that salt consumption is higher and potassium consumption is lower than recommended by the World Health Organization[14,23], both in men and women. Average population salt consumption was 9.0 g per day, almost double the World Health Organization

recommended maximum population target of 5 g per day[14]. Less than 1 in 4 participants met these targets. Salt consumption varied across Governorates, being the lowest in South Sharqiah (5.3 g per day) and the highest in Al-Dhahirah (14.8 g per day). Average population excretion of potassium was 53 mmol per day (equivalent to about 2.36 g per day), lower than the World Health Organization recommended maximum population target of >90 mmol per day, equivalent to approx. 3.90 g per day (assuming urinary potassium being 85% of the intake)[23]. Potassium consumption also varied across Governorates, being the lowest in Al-Wasta (1.41 g per day) and the highest in North Sharqiah (4.25 g per day).

Our questionnaire revealed that half of the population seen often used sauces and condiments (invariably containing high concentrations of salt) but only 10% believed this was too much. A quarter of the surveyed population added salt to food regularly, one in five ate processed food often and more than half of the population ate out at least once a week, with men more likely than women. These results, in addition to those obtained in previous surveys on unhealthy dietary habits, support the national Health Vision set by the Sultanate of Oman to reduce the burden of CVD[32]. This document sets the health visions for the country in 40 years. The comprehensive analyses of many factors affecting the population health and the healthcare system indicate that non-communicable diseases, in the context of increased life-expectancy and population ageing, pose a significant threat to the health of the Omani people and it identify the need to be able to respond to this challenge. Population salt reduction is one of the priorities.

Comparison with countries of the Gulf Cooperation Council (GCC) and of the Arab Peninsula.
In the Gulf Cooperation Council (GCC) countries, populations lead a sedentary lifestyle, both hypertension and obesity are common[17] and they are major contributors to NCDs[33]. The estimated total mortality in GCC countries attributable to NCDs vary from 65% to 78%, with the highest estimates in Bahrain and Saudi Arabia and the lowest in Oman and Qatar, respectively[33]. Salt intake is deemed high in most countries of the Eastern Mediterranean Region (EMR), although there are only a few studies that directly measured population levels, with inconsistent results due to methodological inadequacies[17.18]. The Global Burden of Disease (GBD) estimates of average salt consumption using a Bayesian model suggest that salt consumption in GCC countries may vary from 8.0 g per day in Saudi Arabia to 13.5 g per day in Bahrain[34]. Estimates of salt intake in neighbouring countries would also range between 7.8 g per day in Lebanon and 10.3 g per day in Jordan[34]. The present study is one of the few nationally representative surveys in GCC countries using the gold standard method of

For peer review only - http://bmjopen.bmj.com/site/about/guidelines.xhtml

assessment of dietary salt intake. Its results suggest an intake close to that estimated by the GBD. In addition to the GBD, however, our study also provides, for the first time, direct measures of average population potassium consumption, also targeted by WHO recommendations for cardiovascular prevention[20,23].

Many countries of the EMRO Region of WHO are developing and/or implementing national initiatives to decrease population salt intake[18]. National initiatives include the establishment of national multisectoral committees, the engagement of the government through regulatory measures and legislation (Bahrain, Iran, Jordan, Oman, Qatar), the specification of the food categories prioritized for action such as bread (Kuwait, Qatar) and canned foods (Iran), the development of national benchmarks and targets (Bahrain, Iran, Oman have), dietary guidelines (Afghanistan, Lebanon, Oman, Saudi Arabia), media awareness campaigns (Lebanon, United Arab Emirates), salt labelling, collaborative actions involving the food industry and/or restaurants and food caterers (Kuwait, Qatar, United Arab Emirates), and the monitoring and evaluation of sodium intakes and salt content of foods (Iran, Lebanon, Oman, Qatar).

Comparisons with studies in other countries.

National salt consumption surveys have been carried out in almost all regions of the world, especially in response to the recommendations from the World Health Organization that identified population salt reduction as one of the most cost-effective and feasible approaches to prevent non-communicable diseases[2-3]. Globally, there is a high variation in the readiness of countries to adopt and implement the different aspects of the overall strategy, with low-and-middle income countries still lagging behind[35-36]. Nevertheless, where surveys have been carried out to establish the size of the problem, average levels of salt intake have been very high in countries of Eastern Europe (10.8 g/day in Moldova and 11.6 g/day in Montenegro with potassium about 30% lower than recommended)[37-38], Central Asia (17.2 and 18.8 g/day in two sites of Kazakhstan)[39], China (two-fold North-South gradient from 15.6 to 8.4 g/day and potassium abut 60% lower than recommended)[40] and Australasia (about 9.0 g/day weighted means in Australia and 11.7 g/day in the Fiji Islands)[41-42], indicating urgent need for population interventions. In this respect the average intake in Oman seems reassuring, as the achievement of the set targets appear more feasible that in other countries where intake currently still exceeds the 10 g/day and where potassium consumption is half of what is recommended[23].

For peer review only - http://bmjopen.bmj.com/site/about/guidelines.xhtml

Strengths and Limitations

Our study has several strengths. First, it is population-based survey across the whole country. Second, it included all adults. Thirds, it included both men and women. These study characteristics would allow with greater confidence the extrapolation of results to the whole country population, rather than those conducted in selected groups including patients[43], young female University students[44] or children[45]. Fourth, it used the current preferred methodology for estimating salt consumption. Fifth, we applied a rigorous quality control protocol to ensure completeness of urine collections, and to minimize both under and over-estimations. Current recommendations suggest the use of single complete 24-hour urine samples, collected from a representative population sample to assess the population's current 24-hour dietary sodium ingestion[46]. The role of single spot or short duration timed urine collections in assessing population average sodium intake requires more research. Single or multiple spot or short duration timed urine collections are, on the other hand, not recommended for assessing an individual's sodium intake especially in relationship to health outcomes[46]. Twenty-four hour diet recall and diet records inaccurately measure dietary sodium intake in individuals compared with the gold standard 24 hours urinary excretion[47]. Furthermore, there is poor agreement between estimates of sodium intake from food-frequency questionnaires and 24-hour urine samples[48]. Sixth, it has measured directly the amount of potassium consumption, additional nutrient targeted for cardiovascular prevention[20,23]. Seventh, we standardised fieldwork and used standardised laboratory methodologies across the country. Eighth, all laboratories underwent joint quality control[25].

There are limitations too. First, we included only 57% of the urine samples originally collected from willing individuals. This was due to the stringent quality control that has led to the exclusion of incomplete or erroneous collections[24]. This could have introduced a self-selection bias. The comparison of the baseline characteristics of the studies sample versus the excluded group suggests that the two groups were comparable for general characteristics, with the exception of the latter being two years younger and having a 1.8 mmHg lower diastolic blood pressure. Second, we assessed urinary sodium and potassium excretions only once. Whilst we cannot characterize an individual's intake in such a way[46], there is less likelihood of a bias of group estimates. Third, although we requested participants not to change their diet prior to urine collection, it would be difficult to rule out entirely any bias during collection. Fourth, although we administered a questionnaire to derive knowledge, attitudes and behaviours towards the use of salt, we were unable to establish the

For peer review only - http://bmjopen.bmj.com/site/about/guidelines.xhtml

relative contribution of discretionary sources of salt and commonest foods contributing to salt as well as potassium consumption.

Potential impact

The population in the Sultanate of Oman is of just over 5 million (Ministry of Health Annual Health Report, 2018 estimates), of which about 2.3 million are Omani nationals[4] (surveyed in the present study). Approximately 51% are 25 years or older. To meet a 30% reduction in population salt consumption set by WHO by 2025, the Sultanate of Oman should aim at a 2.7 g per day salt reduction nationally. This reduction would avert 8.1% CVD deaths per year, and more non-fatal events and disabilities[2].

Policy Implications

The Sultanate of Oman has embraced amongst its health priorities the prevention and control of noncommunicable diseases and improvement in nutrition[4] in line with the strategic directions of WHO endorsed by the Eastern Mediterranean Region (EMRO) in 2012 and 2013[18]. Since then several countries have conducted dietary assessment studies in an attempt to assess the population's salt intake[18]. Studies in the area have also attempted to identify the major dietary contributors to sodium intake. Studies are still limited and there are large variations in dietary habits in the Region due to cultural, ethnic, religious and social heterogeneity. The commonest source of salt consumption across the Region is bread[18,49], in all its different forms, with other sources being more relevant in different countries. In Lebanon[50] and in Bahrain[51], dairy products are common sources, whilst in Morocco[52] major contributors to salt consumption include cereal-based products, spices and condiments and milk products. These indications, together with the awareness and behaviours measured, suggest that to reduce population salt consumption in the Sultanate of Oman the following initiatives should be taken: (a) improving salt-related knowledge through health promotion campaigns, (b) measuring major sources of salt consumption, (c) establishing collaborations with local authorities to reduce the amount of salt used in traditional bread making and locally produced condiments, (d) adopting a labelling strategy for imported foods with high salt content.. In addition, the Ministry of Health should develop strategies and methodologies to measure the indicators of population salt consumption[53].

For peer review only - http://bmjopen.bmj.com/site/about/guidelines.xhtml

Conclusions

This study demonstrates that salt consumption in the Sultanate of Oman is high and should be reduced through a public health action aiming at the entire population. Likewise, potassium consumption is particularly low. The KABs survey indicates areas of limited awareness. Education of the dangers of high salt consumption and where salt is hidden, of the benefits of increasing potassium through fruit, vegetables, nuts and legumes, alongside accurate labelling and marketing of food, surveillance to measure and monitor salt use, and reformulating bread are all important elements of an effective national salt reduction programme[18,19,54].

Supplementary materials: The following are available online at

Text S1: Material and Methods (full text). **Table S1**. Comparison of the general characteristics of the participants in the salt and potassium survey with those participating in the 2017 national WHO STEPS Survey. **Table S2.** Characteristics of the excluded participants and comparison with those included in the study.

Acknowledgments: We carried out the present analysis under the terms of reference of the WHO Collaborating Centre for Nutrition at the University of Warwick. The authors would like to express gratitude to the EMRO WHO Regional Office (Dr Ayoub Al-Jawaldeh) and the WHO Country Office of Oman (Dr Ruth Mabry) for facilitating the study, the Department of Food Science and Human Nutrition of Sultan Qaboos University (Dr Lyutha Al-Subhi) and other members of the Ministry of Health (Dr Amel Ibrahim, Dr Ruqaya Balushi and Dr Salima for their helpful discussions in the preparation of the protocol. Special thanks go to the team of the Ministry of Health of the Sultanate of Oman involved in the preparation of the survey and data collection. Finally, we wish to express our gratitude to His Excellency the Undersecretary for Health Affairs of the Sultanate of Oman, Dr Mohammed bin Saif Al Hosni, and His Advisor, Dr Mahmood Shaban, for their advice, endorsements and support at a National level.

Author Contributions: FPC developed the study design and protocol, contributed to the analysis and drafted the manuscript, AA-M trained local teams, co-ordinated quality control and data collection. MM and LD carried out quality control and statistical analysis. AA-M, SKJ, WNA-S, ADP, HA-K, ZA-B, JI, AA-H coordinated the study, carried out the fieldwork and liaised with the local laboratory. MM helped with the drawing of the stratified random sample from the sampling frame. All authors contributed to the interpretation of the findings and they contributed significantly to the final version of the manuscript. FPC is the guarantor.

For peer review only - http://bmjopen.bmj.com/site/about/guidelines.xhtml

Tuesday, 02 Jun 2020 – v11- R2

Data Sharing Statement: No individual participant data will be available. Study protocol available in Supplementary Material. Any other data sharing proposal must be submitted in writing to the Ministry of Health of the Sultanate of Oman.

References

1. GBD 2015 Mortality and Causes of Death Collaborators. Global, regional, and national life expectancy, all-cause mortality, and cause-specific mortality for 249 causes of death, 1980–2015: A systematic analysis for the Global Burden of Disease Study 2015. *Lancet* 2016; 388: 1459–1544.
2. World Health Organization. Global Action Plan for the Prevention and Control of NCDs 2013–2020; World Health Organization: Geneva, Switzerland, 2013; pp. 1–103.
3. World Health Organization. Tackling NCDs: "Best buys" and other recommended interventions for the prevention and control of noncommunicable diseases; 2017. http://www.who.int/ncds/management/best-buys/en/
4. World Health Organization. Oman Health Profile 2018. World Health Organization. Regional Office for the Eastern Mediterranean, Cairo, Egypt, 2016; pp. 1-37. WHO-EM/HST/227/E
5. Al-Mawali A. Non-communicable diseases: shining a light on cardiovascular disease, Oman's biggest killer. *Oman Med J* 2015; 30(4): 227-228.
6. Al Riyami A, Elaty MAA, Morsi M, et al. Oman World Health Survey: Part 1: Methodology, sociodemographic profile and epidemiology of non-communicable diseases in Oman. *Oman Med J* 2012; 27(5): 425-443.
7. Aburto NJ, Ziolkovska A, Hooper L, Elliott P, Cappuccio FP, Meerpohl J. Effect of lower sodium intake on health: Systematic review and meta-analysis. *Br. Med. J.* 2013; 346: f1326.
8. He FJ, Li J, MacGregor GA. Effect of longer-term modest salt reduction on blood pressure: Cochrane systematic review and meta-analysis of randomised trials. *Br. Med. J.* 2013; 346: f1325.
9. Strazzullo P, D'Elia L, Kandala N.-B, Cappuccio FP. Salt intake, stroke, and cardiovascular disease: Meta-analysis of prospective studies. *Br. Med. J.* 2009; 339: b4567.
10. Cook NR, Cutler JA, Obarzanek E, et al. Long term effects of dietary sodium reduction on cardiovascular disease outcomes: Observational follow-up of the Trials of Hypertension Prevention (TOHP). *Br. Med. J.* 2007; 334: 885–888.

For peer review only - http://bmjopen.bmj.com/site/about/guidelines.xhtml

11. Cappuccio FP. Cardiovascular and other effects of salt consumption. *Kidney Int.* 2013; 3: 312–315.
12. D'Elia L, Rossi G, Schiano di Cola M, et al. Meta-analysis of the effect of dietary sodium restriction with or without concomitant renin–angiotensin–aldosterone system-inhibiting treatment on albuminuria. *Clin. J. Am. Soc. Nephrol.* 2015; 10: 1542–1552.
13. D'Elia L, Galletti F, La Fata E, Sabino P, Strazzullo P. Effect of dietary sodium restriction on arterial stiffness: Systematic review and meta-analysis of the randomized controlled trials. *J. Hypertens.* 2018; 36: 734–743.
14. World Health Organization. Guideline. Sodium Intake for Adults and Children. World Health Organization (WHO): Geneva, Switzerland, 2012.
15. Brown IJ, Tzoulaki I, Candeias V, Elliott P. Salt intakes around the world: Implications for public health. *Int. J. Epidemiol.* 2009; 38: 791–813.
16. Cappuccio FP, Capewell S. Facts, issues, and controversies in salt reduction for the prevention of cardiovascular disease. *Funct. Food Rev.* 2015; 7: 41–61
17. Alhamad N, Almalt E, Alamir N, Subhakaran M. An overview of salt intake reduction efforts in the Gulf Cooperation Council countries. *Cardiovasc. Diagn. Ther.* 2015; 5(3): 172-177.
18. Al Jawaldeh A, Rafii B, Nasreddine L. Salt intake reduction strategies in the East Mediterranean Region. *East Mediterr. Health J.* 2018; 24(12): 1172-1180.
19. Cappuccio FP, Capewell S, Lincoln P, McPherson K. Policy options to reduce population salt intake. *Br. Med. J.* 2011; 343: 402–405.
20. Aburto NJ, Hanson S, Gutierrez H, Hooper L, Elliott P, Cappuccio FP. Effect of increased potassium intake on cardiovascular risk factors and disease: Systematic review and meta-analyses. *Br. Med. J.* 2013; 346: f1378.
21. D'Elia L, Barba G, Cappuccio, FP, Strazzullo P. Potassium Intake, Stroke, and Cardiovascular Disease. A meta-analysis of Prospective Studies*. J. Am. Coll. Cardiol.* 2011; 57: 1210–1219.
22. D'Elia L, Iannotta C, Sabino P, Ippolito R. Potassium rich-diet and risk of stroke: Updated meta-analysis. *Nutr. Metab. Cardiovasc. Dis.* 2014; 24: 585–587.
23. World Health Organization. Guideline: Potassium Intake for Adults and Children. World Health Organization (WHO): Geneva, Switzerland, 2012; pp. 1–52.
24. World Health Organization. How to Obtain Measures of Population-Level Sodium Intake in 24-Hour Urine Samples. World Health Organization. Regional Office of the Eastern Mediterranean, Cairo, Egypt, 2018; pp. 1–51. WHO-EM/NUT/279/E.

For peer review only - http://bmjopen.bmj.com/site/about/guidelines.xhtml

25. World Medical Association. Declaration of Helsinki. Recommendations guiding doctors in clinical research. *Bull. World Health Org*. 2008; 86: 650–651.
26. World Health Organization. The WHO STEPwise approach to chronic disease risk factor surveillance (STEPS). World Health Organization, Geneva, Switzerland, 2014. https://www.who.int/ncds/surveillance/steps/STEPS_Instrument_v2.1.pdf
27. Mancia G, Fagard R, Narkiewicz K, et al. 2013 ESH/ESC Guidelines for the management of arterial hypertension: The Task Force for the management of arterial hypertension of the European Society of Hypertension (ESH) and of the European Society of Cardiology (ESC). *J. Hypertens.* 2013; 31: 1281–1357.
28. Oesch U, Ammann D, Simon W. Ion-selective membrane electrodes for clinical use. *Clin. Chem.* 1986; 32: 1448–1459.
29. Junge W, Wilke B, Halabi A, Klein G. Determination of reference intervals for serum creatinine, creatinine excretion and creatinine clearance with an enzymatic and a modified Jaffé method. *Clin. Chim. Acta* 2004; 344: 137–148.
30. Lucko AM, Doktorchik C, Woodward M, et al. Percentage of ingested sodium excreted in 24-hour urine collections: A systematic review and meta-analysis. *J Clin. Hypertens.* 2018; 20: 1220–1229.
31. Stamler J, Elliott P, Chan Q, for the INTERMAP Research group. INTERMAP Appendix tables. *J. Hum. Hypertens.* 2003; 17: 665–758.
32. Ministry of Health. *Health Vision 2050. The Main Document*. Undersecretariat for Planning Affairs, Ministry of Health, Sultanate of Oman 2014; pp. 1-212. http://www.nationalplanningcycles.org/sites/default/files/planning_cycle_repository/oman/health_vision_2050_oman.pdf (accessed Jan 14, 2020).
33. World Health Organization. Noncommunicable Diseases Country Health Profiles, 2019. http://www.emro.who.int/entity/statistics/country-health-profiles.html
34. Powles J, Fahimi S, Micha R, et al. Global, regional and national sodium intakes in 1990 and 2010: a systematic analysis of 24h urinary sodium excretion and dietary survey worldwide. *BMJ Open* 2013; 3: e003733.
35. Rodriguez-Fernandez R, Siopa M, Simpson SJ, et al. Current salt reduction policies across gradients of inequality-adjusted human development in the WHO European region: minding the gaps. *Public Health Nutr* 2014; 17(8): 1894-904
36. Trieu K, Neal B, Hawkes C, et al. Salt reduction initiatives around the world. A systematic review of progress towards the global target. *PLoS ONE* 2015; 10(7): e0130247.
37. D'Elia L, Brajovic M, Klisic A, et al. Sodium and potassium intake, knowledge attitudes and behaviour towards salt consumption, amongst adults in Podgorica, Montenegro. *Nutrients* 2019; 11: 160.

For peer review only - http://bmjopen.bmj.com/site/about/guidelines.xhtml

38. D'Elia L, Obreja G, Ciobanu A, et al. Sodium, potassium and iodine intake in a national adult population sample of the Republic of Moldova. *Nutrients* 2019; 11: 2896.
39. Trieu K, Ospanova F, Sharmanov T, et al. A15481 Mean urinary salt excretion in two Kazakhstan regions – one of the highest in the world. *J Hypertens* 2018; 36: e334.
40. Tan M, He FJ, Wang C, MacGregor GA. Twenty-four-hour urinary sodium and potassium excretion in China: a systematic review and meta-analysis. *J Am Heart Assoc* 2019; 8: e012923.
41. Land M-A, Webster J, Christoforou A, et al. Salt intake assessed by 24 h urinary sodium excretion in a random and opportunistic sample in Australia. *BMJ Open* 2014; 4(1): e003720.
42. Pillay A, Trieu K, Santos JA, et al. Assessment of a salt reduction intervention on adult population salt intake in Fiji. *Nutrients* 2017; *9*(12): 1350.
43. Alkhunaizi AM, Al Jishi HA, Al Sadah ZA. Salt intake in Eastern Saudi Arabia. *East Mediterr. Health J.* 2013; 19(11): 915-918.
44. Tayel DI, Amine AK, Elzawi AK. Dietary intake of nutrients related to bone health among Alexandria University female students, Egypt. *Food and Public Health* 2013; 3(6): 329-335.
45. Kelishadi R, Gheisari A, Zare N, Farajian S, Shariatinejad K. Salt intake and the association with blood pressure in young Iranian children: first report from the Middle East and North Africa. *Int. J. Prev. Med.* 2013; 4(4): 475-483.
46. Campbell NRC, He FJ, Tan M, et al. The International Consortium for Quality Research on Dietary Sodium/Salt (TRUE) position statement on the use of 24-hour, spot and short duration (<24-hours) timed urine collections to assess dietary sodium intake. *J Clin Hypertens* 2019; 21: 700-709.
47. McLean RM, Farmer VL, Nettleton A, et al. Twenty-Four– Hour Diet recall and Diet records compared with 24-hour urinary excretion to predict an individual's sodium consumption: A Systematic Review. *J Clin Hypertens* 2018; 20: 1360–1376.
48. McLean RM, Farmer VL, Nettleton A, et al. Assessment of dietary sodium intake using a food frequency questionnaire and 24-hour urinary sodium excretion: a systematic literature review. J *Clin Hypertens* 2017; 19: 1214–1230.
49. Almedawar MM, Nasreddine L, Olabi A, et al. Sodium intake reduction efforts in Lebanon. *Cardiovasc Diagn Ther* 2015; 5(3): 178-185.
50. Nasreddine L, Akl C, Al-Shaar L, Almedawar MM, Isma'eel H. Consumer knowledge, attitudes and salt-reated behaviour in the Middle-East: the case of Lebanon. *Nutrients* 2014; 6: 5079-5102.
51. Ministry of Health (Bahrain). National nutrition survey for adult Bahrainis aged 19 years and above. 2002
52. Derouiche A, Jafri A, El Kardi Y. Assessment of salt, fat content, and intake in largely consumed foods in Morocco. Report of a pilot study. 2014; p. 1-92

For peer review only - http://bmjopen.bmj.com/site/about/guidelines.xhtml

53. Bonita R, Magnusson R, Bovet P, et al. Country actions to meet UN commitments on non-communicable diseases: a stepwise approach. *Lancet* 2013; 381: 575-584.
54. Farrand C, He FJ, MacGregor GA. Reducing population salt intake in the Eastern Mediterranean Region – time for urgent action. *Eastern Med. Health J.* 2014; 20(12): 761-764.

Figure 1. Stepwise procedure for the selection of valid participants according to protocol adherence, quality control and completeness of 24h urine collections.

For peer review only - http://bmjopen.bmj.com/site/about/guidelines.xhtml

Tuesday, 02 Jun 2020 – v11- R2

Table 1. Geographical sampling from the Sultanate of Oman.

Governorate	Valid 24h urine collections	%
Muscat	67	11.8
Dhofar	79	13.9
Al-Dakhlia	45	7.9
North Sharqiah	36	6.3
South Sharqiah	45	7.9
North Batina	81	14.2
South Batina	53	9.3
Al-Dhahirah	46	8.1
Al Buraymi	84	14.8
Musandam	9	1.6
Al-Wasta	24	4.2
**Total**	**569**	**100.0**

For peer review only - http://bmjopen.bmj.com/site/about/guidelines.xhtml

Tuesday, 02 Jun 2020 – v11- R2

Table 2. Characteristics of the participants

Variable	All (n=569)	Men (n=193)	Women (n=376)
Age (years)	39.4 (13.1)	38.7 (14.3)	39.8 (12.5)
Height (cm) ʃ	159.4 (11.2)	167.9 (9.7)	154.9 (9.2)*
Weight (kg) ʃ	74.9 (21.5)	81.4 (22.5)	71.4 (20.1)*
BMI (kg/m^2) ʃ	29.3 (7.2)	28.9 (7.6)	29.5 (7.0)
Waist circumference (cm) ʃ	93.8 (15.7)	95.0 (15.7)	93.2 (15.7)
Hip circumference (cm) ʃ	104.5 (15.0)	102.6 (13.7)	105.5 (15.6)†
Systolic blood pressure (mm Hg) ‡	125.9 (18.2)	134.0 (17.0)	121.7 (17.3)*
Diastolic blood pressure (mm Hg) ‡	80.9 (10.7)	83.4 (11.5)	79.7 (10.1)*
Pulse rate (b/min) ‡	79.8 (10.5)	78.5 (11.8)	80.4 (9.8)†
Hypertension N (%) ‡	155 (27.4)	74 (38.5)	81 (21.7)*

Results are mean (SD) or N(%),
‡ 3 missing values (1m, 2w) (0.5%); ʃ 18 missing values (1m, 17w) (4%);
* p<0.01, † p<0.05 when compared to men
Hypertension: SBP/DBP ≥140/90 mmHg or on current therapy for high blood pressure

For peer review only - http://bmjopen.bmj.com/site/about/guidelines.xhtml

Tuesday, 02 Jun 2020 – v11- R2

Table 3. Daily urinary excretions of volume, sodium, potassium and creatinine, estimates of salt and potassium intake, and proportion of participants meeting WHO recommended targets for salt and potassium consumption.

**Variable**	**All (n=569)**	**Men (n=193)**	**Women (n=376)**
Volume (mL/24h)	1354 (725)<br>1129 (855-1618)	1392 (712)<br>1150 (900-1721)	1335 (731)*<br>1122 (827-1593)
Sodium (mmol/24h)	144.3 (78.8)<br>129.6 (85.7-187.4)	154.2 (87.4)<br>135.4 (87.1-204.8)	139.2 (73.6)*<br>126.8 (83.9-179.4)
Potassium (mmol/24h)	52.6 (32.6)<br>46.4 (31.4-64.9)	56.4 (32.4)<br>50.9 (33.8-73.2)	50.6 (32.5)*<br>44.7 (30.2-61.6)
Creatinine (g/24h)	1.33 (0.71)<br>1.18 (0.86-1.63)	1.72 (0.87)<br>1.61 (1.16-2.12)	1.13 (0.52)†<br>1.02 (0.81-1.36)
Salt intake (g/day)	9.0 (4.9)<br>8.1 (5.3-11.7)	9.6 (5.5)<br>8.5 (5.4-12.8)	8.7 (4.6)*<br>7.9 (5.2-11.2)
Potassium intake (g/day)	2.36(1.46)<br>2.08 (1.41-2.91)	2.53 (1.45)<br>2.28 (1.52-3.28)	2.27 (1.46)*<br>2.00 (1.35-2.76)
Salt <5g/day N (%)	124 (21.8)	40 (20.7)	84 (22.3)
Potassium >90 mmol/day N (%)	52 (9.1)	24 (12.4)	28 (7.4)#

Results are mean (SD) and median (25th-75th percentile) or N (%); *p<0.05; #p=0.008, †p<0.001 when compared to men

Tuesday, 02 Jun 2020 – v11- R2

Table 4. Knowledge, attitudes and behaviours towards salt consumption.

**Question**	**All (n=569)**	**Men (n=193)**	**Women (n=376)**
How often do you add salt or salty sauces to your food?			
Often/Always	28.1 %	22.8 %	30.8 % *
Sometimes	21.3 %	17.1 %	23.4 %
Rarely/Never	50.6 %	60.1 %	45.8 %
How often is salt, salty seasoning or salty sauces added in cooking or preparing food at home? ‡			
Often/Always	47.0 %	44.8 %	48.1 % #
Sometimes	16.5 %	12.5 %	18.6 %
Rarely/Never	36.5 %	42.7 %	33.3 %
How often do you eat processed food? ‡			
Often/Always	22.3 %	22.8 %	22.1 %
Sometimes	38.3 %	35.8 %	39.5 %
Rarely/Never	39.4 %	41.4 %	38.4 %
How much salt or salty sauces do you think you consume? ‡			
Too much/Far too much	6.9 %	7.8 %	6.4 %
Just the right amount	66.8 %	61.3 %	69.7 %
Too little/Far too little	26.3 %	30.9 %	23.9 %
Do you think that too much salt or salty sauces could cause a serious health problem? ‡			
Yes	89.1 %	90.1 %	88.6 %

Results are percentages *p=0.005; #p=0.04 when compared to men. ‡ reduced numbers due to missing values

Table 5. Frequency of other dietary patterns

**Question**	**All (n=569)**	**Men (n=193)**	**Women (n=376)**
In a typical week, on how many days do you eat fruit? ‡			
< 5	32.0 %	40.4 %	27.7 %**
≥ 5	68.0 %	59.6 %	72.3 %
How many servings of fruit do you eat on one of those days? ‡			
<3	45.5 %	41.8 %	47.2 %
≥3	54.5 %	58.2 %	52.8 %
In a typical week, on how many days do you eat vegetables? ‡			
< 5	24.1 %	24.9 %	23.7 %
≥ 5	75.9 %	75.1 %	76.3 %
How many servings of vegetables do you eat on one of those days? ‡			
<3	59.9 %	65.9 %	56.9 %#
≥3	40.1 %	34.1 %	43.1 %
What type of oil or fat is most often used for meal preparation in your household? ‡			
Vegetable oil	90.8 %	91.1 %	90.7 %
Other (lard, suet, butter, ghee)	9.0 %	8.9 %	9.0 %
None used	0.2 %	0	0.3 %
On average, how many meals per week do you eat that were not prepared at home? ‡			
0	45.8 %	32.1 %	52.8 %*
≥1	54.2 %	67.9 %	47.2 %

Results are percentages *p<0.0001; **p=0.002; #p=0.04 vs men. ‡ reduced numbers due to missing values

For peer review only - http://bmjopen.bmj.com/site/about/guidelines.xhtml

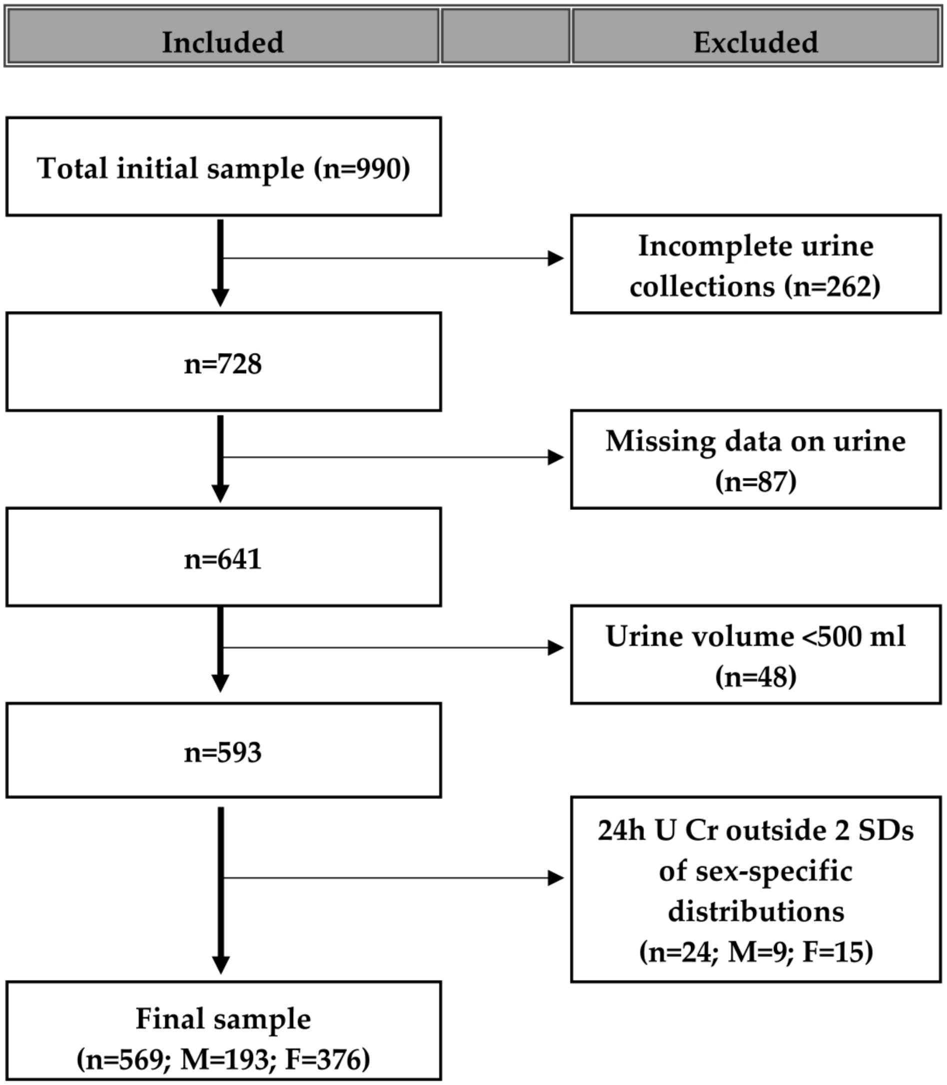

Figure 1. Stepwise procedure for the selection of valid participants according to protocol adherence, quality control and completeness of 24h urine collections.

SUPPLEMENTARY MATERIAL

TEXT S1. MATERIAL AND METHODS

1.1 Study Design

A national cross-sectional population-based survey was conducted using a representative sample of the Sultanate of Oman based on the WHO Stepwise approach to Surveillance (STEPS) of NCD risk factors (1), comprising the following aspects:

- **Step 1:** This consisted of face-to-face interviews using advanced standardized nation-specific version of the STEPS questionnaire and locally-adapted show cards to facilitate the understanding and operationalization of some questions. Socio-demographic characteristics, key behavioural risk factors, lifestyles, eye and ear health, history of chronic diseases and health care coverage were also elicited to better define exposure and health care seeking and control.
- **Step 2:** This involved physical measurements (e.g. weight, height, waist, hip circumference), and determination of blood pressure, heart rate, and vision function to investigate biological risk factors such as hypertension, overweight, obesity, and vision issues.
- **Step 3:** This aimed at determinations of biochemical markers levels (e.g. fasting capillary blood for glucose and lipid profile, and non-fasting urinary samples for sodium and creatinine) to identify hypercholesterolemia, hyperglycaemia, and sodium intake.

1.2 Sample Design

2.1 Target Population: The population of interest was all non-institutionalized persons, which included all men and women 18 years of age or older (Omani & non-Omani) who reside in the country.

2.2 Inclusion population: The target population includes all persons who consider the country to be their usual place of residence. This definition comprises those individuals residing in the country even though they may not be considered a citizen of the country.

2.3 Exclusion population: Those household members who were younger than 18 years of age; persons who have cognitive impairment that hampered understanding the questions to provide clear feedback; visitors (tourists) to the country; and, institutionalized people or those who indicated their usual place of residence was a military base, labour camps or group quarters, were excluded from the survey.

1.3 Sample Frame

For the 2010 National census, the whole of the Sultanate of Oman was divided into 15,077 census blocks. A census block was defined as a collection of units (residential units), which

For peer review only - http://bmjopen.bmj.com/site/about/guidelines.xhtml

includes 60 units or less, and it could involve one or more enumeration areas. The census block is the smallest unit of field work for census data collectors. A total of 399,274 households[1] were identified in these blocks (260,120 Omani and 139,154 non-Omani). Households for Omanis and non-Omanis could not be differentiated unless physically visited as they lived closely together and there were no specific places (blocks) for non-Omani. The number of blocks and households in each governorate was provided in the table below according to the National Census 2010 and the map below shows the distribution of blocks over the Sultanate of Oman by governorates (National Centre for Statistics & Information (NCSI), Sultanate of Oman). The same census blocks were updated and used in this NCD Risk Factor Survey as shown in Table 1.

Table 1: Number of blocks and households by governorate based on 2010 National Census comprising the sampling frame for Oman NCD risk factors survey, 2017

Governorate	Number of blocks	Number of Omani households
**Muscat**	3514	62299
**Dhofar**	1768	17926
**Ad Dakhiliyah**	1712	34611
**North Sharqiyah**	1173	20044
**South Sharqiyah**	1097	22455
**North Batinah**	2248	47193
**South Batinah**	1335	29355
**Al-Dhahirah**	884	14386
**Al-Buraimi**	531	5939
**Musandam**	513	3216
**Al-Wusta**	302	2696
**Total**	**15077**	**260120**

1.3.1 Mapping and listing update

Two persons were appointed and trained to perform the mapping and listing operation for each governorate by using 2010 census maps from the National Centre for Statistics and Information. Personnel visited selected blocks to update maps that depicted all the households within the selected block and update the list of households within those blocks, including

1 A “Household (HH)” was defined as “either a one-person household, defined as an arrangement in which one person makes provision for his or her own food or other essentials for living without combining with any other person to form part of multi-person household or a multi-person household, defined as a group of two or more persons both related and unrelated living together who make common provision for food or other essentials for living”.

For peer review only - http://bmjopen.bmj.com/site/about/guidelines.xhtml

additional information (e.g. house number - Name of household head – nationality – language – description if needed).

1.4 Sampling Design

A multi-stage stratified cluster sampling was designed to select 9053 eligible subjects. Stratification was made on two factors: governorate and nationality (Omani and Non-Omani). The sample was drawn from the 2010 census block area (clusters). An ***equal size sample (cluster)*** was systematically randomly selected from each governorate. Selected clusters (blocks) had to be updated before the households were selected for survey (see previous section).

1.4.1 Rationale for preferring equal sample selection rather than proportional to population size

1. To get the desired precision and estimates of overall results and figures at the governorate level.
2. Proportional allocation would have resulted in a very small sample in a small governorate e.g. Al-Wusta with low precision in the estimate.
3. Equal sampling allowed high precision in individual stratum.
4. The experience from previous surveys in Oman. The lowest response rate was in Muscat governorate and one third of population live in Muscat so if proportional selection was used this would have directly affected the overall response rate.

In the first stage, all governorates in the Sultanate of Oman (11 governorates) were selected.

In the second stage, we stratify blocks (PSU) based on geographical location, 550 clusters, from all over the Sultanate of Oman (50 from each governorate) using systematic random sampling (Secondary Sampling Units (SSU)).

In the third stage: [Tertiary Sampling Unit (TSU)], the following was considered:

i) The households in each cluster were listed within each governorate i.e. households from the first cluster, then households from the second cluster till households from the 50th cluster.

ii) The households in all clusters were aggregated into two lists by nationality (Omani and Non-Omani households) in each governorate

iii) 823 households were systematically and randomly selected from list of all households according to above in each governorate, from these two lists (Omani and Non-Omani household lists) according to the ratio of Omani and Non-Omani households in each governorate.

In the fourth stage: Ultimate sampling unit USU), One eligible individual from each household aged 18 years old or older was selected randomly by a program on the Android tablet.

For peer review only - http://bmjopen.bmj.com/site/about/guidelines.xhtml

1.4.2 Sample Size

A WHO STEPS standard formula was used in the calculation of the sample size based on the guidelines/recommendations of the STEPS survey.

$n= Z^2 * P (1-P)/d^2$

Where;

n= the required sample size

Z= the probability value associated with the confidence level

P= the prevalence rate of NCDs risk factors in the country

d= the desired margin of error (precision).

In turn:

Z= 1.96 (95% confidence interval as recommended)

P= 0.5 (the conservative value of prevalence rate)

e= 0.05 (as recommended in the guidelines)

Using these values, the initial calculation was: n= 384 households

Also taken into account for sample size calculations were: a value of design effect, as recommended in STEPS surveys, to be 1.5; and, an anticipated response rate of 70% was estimated. By adjusting the sample by these factors, the sample size per cluster (governorate) results in:

$$n = \frac{384\ x\ 1.5}{0.7} = 823 \text{ households}$$

To get the desired precision and overall figures adequate for age-sex groups and for overall estimate on governorates level, the sample size was:

n= 823 x 11 = 9053 households, with one individual selected per household

The 9053 households were distributed equally by governorate (823), proportional to nationality, according to the ratio of Omani and non-Omani households in each governorate as in Table 2.

Table 2: Oman STEPS survey sample size distributed, by governorate and nationality, 2017

Governorate	Selected no. of blocks in each governorate	Omani	Non-Omani	Total Households

For peer review only - http://bmjopen.bmj.com/site/about/guidelines.xhtml

**Muscat**	50	318	505	**823**
**Dhofar**	50	495	328	**823**
**Ad Dakhiliyah**	50	763	60	**823**
**North Sharqiyah**	50	520	303	**823**
**South Sharqiyah**	50	667	156	**823**
**North Batinah**	50	677	146	**823**
**South Batinah**	50	699	124	**823**
**Al-Dhahirah**	50	586	237	**823**
**Al-Buraimi**	50	722	101	**823**
**Musandam**	50	713	110	**823**
**Al-Wusta**	50	663	160	**823**
**Total**	**550**	**6823**	**2230**	**9053**

1.5 Data collection instruments and procedures used in the survey

1.5.1 Selection of participants

Upon selection of a household, all potential individuals for interview were identified and their age and gender recorded in a household list, subsequently used to determine selection probabilities and response rate. To randomly select an eligible individual from a household, the Kish method was used using an electronic tablet and software. Once individuals were selected, they were informed about the survey aims and asked to provide their consent to participate in the interview and subsequent measurement procedures.

1.5.2 Questionnaire (STEP 1)

An advanced standardized[2] country-specific version of the questionnaire, based on the Core and Expanded version of the WHO STEPS Instrument for Chronic Disease Risk Factor Surveillance[3] and country-specific requirements, contained 11 Core, 1 Optional and 4 country-specific modules, to determine socio-demographic characteristics of participants, key behavioural risk factors (tobacco use, harmful alcohol consumption, diet with low fruits and vegetable intake, high salt intake, physical inactivity), given lifestyle advises, eye and ear health profile, history of chronic diseases, and health care coverage for diabetes, hypertension and dyslipidaemia, as well as cervical and breast screening.

In order to enhance the comparability with other countries in Arab region, the questionnaires from the Kuwait (2014), the Qatar (2012), the Bahrain (2007) and the Saudi Arabia (2005) STEPS surveys were taken into consideration. In addition, the questionnaire was translated from the original English version into Arabic as well as back translated, adapted to the local environment and needs, and piloted on 10 eligible respondents in terms of wording and

2 validated
3 Reference: The WHO STEPS Instrument for Chronic Disease Risk Factor Surveillance (1)

For peer review only - http://bmjopen.bmj.com/site/about/guidelines.xhtml

understanding. The data collection was conducted in two languages, namely: Arabic and English.

Table 3: Questionnaire content by STEPS survey and county-specific modules and questions in the Oman survey, by type, 2017

	STEP and county-specific modules content	Questions		
**I**	**Core modules (11)**	**Core**	**Expanded**	**Country-specific**
1	Demographic information	4	5	3
2	Tobacco use	11	7	
3	Alcohol consumption	12		
4	Diet, including dietary salt	8	3	2
5	Physical activity	15	1	
6	History or raised blood pressure	5		1
7	History of diabetes	6		1
8	History of raised total cholesterol	5		1
9	History of cardiovascular disease	3		
10	Lifestyle advise	1		
11	Cancers (cervical cancer screening)	1		3
	**Subtotal 1**	71	16	11
**II**	**Optional Module (1)**			
1	Tobacco policy	7		8
	**Subtotal 2**	7		8
**III**	**Country-specific Modules (4)**			
1	Family history of chronic disease			8
2	Asthma			11
3	Eye health			3
4	Ear health			4
	**Subtotal 3**			26
**Grand Total: 139**		**78**	**16**	**45**

1.5.2.1 Demographic information

All eligible household members aged 18 and above were listed with one eligible member selected randomly to answer the demographic information questionnaire. Demographic information was assessed in terms of age, sex, marital status, educational status, employment status, and family income.

1.5.2.2 Tobacco use

Tobacco use was assessed in terms of current smoking (past 30 days), whether daily or non-daily, and former smoking or never smoking status, age of initiation and duration of smoking, type and quantity of tobacco use daily or weekly, smokeless tobacco use type and frequency, and exposure to second-hand smoke at home or workplace. Smoking cessation attempts, having

For peer review only - http://bmjopen.bmj.com/site/about/guidelines.xhtml

received health professionals' advice, age and time since stopping to smoke, recognizing tobacco advertisement, promotion or sponsorship, all while applying 33 questions[4] were also asked. To facilitate recognition of types of tobacco use, data collectors used show cards, depicting types of commonly used tobacco products. Likewise, the above metrics were determined for users of smokeless tobacco products.

1.5.2.3 Alcohol consumption

Alcohol consumption was assessed using the concept of a standard drink. A standard drink is any drink containing about 10 g of pure alcohol. Accordingly, determinations have been made from different types of alcoholic beverages consumed, as follows: e.g. 30 ml of spirits, 120 ml of wine or 285 ml of beer. Again, data collectors used show cards depicting types of containers commonly used to consume alcoholic beverages as standard drinks, to determine consumption over 30 and 7 days prior to interview. Also, in an attempt to quantify and estimate total alcohol consumption, interviewers considered not only the most frequent but all types of alcohol consumed (e.g. wine, beer and spirits) and the amount of drinks on such occasions. Also included in the questionnaire were aspects about stopping alcohol consumption for health reasons or impacts.

Respondents who reported consuming alcohol within the past 30 days were classified as current drinkers, while those who identified absence of alcoholic beverages within previous 12 months as abstainers or ex-drinkers. Three risk categories were used to classify respondents who consumed alcohol according to the average amount of alcohol consumed per occasion. Furthermore, heavy ("binge") drinking patterns were determined according to largest number of drinks per drinking occasion and the percentage of those having consumed six or more standard drinks on one occasion during the past 7 days.

1.5.2.4 Diet

To assess the dietary patterns of the surveyed population, the respondents were asked about frequency of fruit and vegetable consumption, mean number of portions of these foods consumed daily and weekly, type of oils and fat used for meal preparation, number of meals eaten outside the household per week and the amount of salt added, and/or salty sauces used or processed food or consumed daily, using 13 questions.

Sufficient consumption of fruit and vegetables was assessed in terms of the number of servings and also compared to WHO recommended number of ≥ 5 servings/day and ≥ 5 day/week, with

[4] Based on STEPS and GATS questionnaires (2)

For peer review only - http://bmjopen.bmj.com/site/about/guidelines.xhtml

a serving being equal to 80 g. Show cards were used to facilitate the collection data on fruit and vegetable consumption on a typical day. Oil and fat intake show cards were also shown to assess about the type of oil or fat most frequently used for preparing food or cooking.

In turn, salt consumption was assessed by asking about frequency of addition of salt or a salty sauce to food during preparation, or before or while eating; and/or frequency of consumption of processed food high in salt. Participants were also asked about their perception of the quantity of salt they consumed and its link with health problems, as well as about the importance of reducing salt intake, and the measures undertaken to control it. WHO recommends a reduction to <2 g/day sodium (5 g/day salt) in adults (3).[5]

Population mean number of daily portions and of days per week consuming fruits and/or vegetables were calculated. Also, the percentage distribution of respondents according to their servings consumed per day and those meeting the WHO recommendation of fruits and vegetables intake/day were determined.

Regarding salt intake, the proportions of people reporting how often they added salt to foods before eating or when preparing foods, as well as those who think they eat too much salt were determined. Percentage of participants were further determined according to their belief on the importance of salt in diet and whether they thought their consuming to much salt can cause serious problems.

1.5.2.5 Physical activity

Physical activity was assessed based on frequency, duration and intensity of physical activity at three segments: work (paid/ unpaid in and outside home), during transportation and on leisure time, for at least 10 minutes or more continuously per day, using a set of 16 questions[6]. Show cards were used to depict different types and places of physical activity.

According to WHO global recommendations on physical activity for (good) health, throughout a normal week adult should do at least the following amount of exercise (including activity for work, as well as during transport and leisure time): 150 minutes of moderate-intensity physical activity; or 75 minutes of vigorous-intensity physical activity; or an equivalent combination of moderate- and vigorous-intensity physical activity. Mean and median minutes of physical activity per day according to place were computed; as a complement, time spent on sedentary activities on average per day was also calculated. The proportion of respondents not meeting

[5] http://www.who.int/nutrition/publications/guidelines/sodium_intake_printversion.pdf
[6] Based on WHO Global Physical Activity Questionnaire (version 2) (4)

For peer review only - http://bmjopen.bmj.com/site/about/guidelines.xhtml

the WHO recommendations was also calculated. Likewise, the proportion of participants according to levels of physical activity as recommended by WHO were determined.

1.5.3 Physical measurements (STEP 2)

1.5.3.1 Blood pressure

Resting blood pressure levels, both systolic (SBP) and diastolic (DBP), were measured using Omron M3 digital blood pressure device as recommended by WHO. The measurements were repeated three times and the three readings were recorded. In order to obtain the measurements under relaxed conditions, persons were asked to void their bladder if needed, resting for 10-15 minutes after the interview, and not having drunk coffee before or during the measurement. In preparation for measurements, participants were asked to sit straight without crossing their legs. Blood pressure was measured placing a universal cuff on the left arm, which was placed with the palm face upward on a table surface at the level of the heart. While taking the readings participants were asked to remain silent. Repeat measurements were taken at 3-minute interval.

Participants were classified according to their blood pressure readings in the following categories: *normal* if their SBP and DBP readings were <140 mm Hg and <90 mmHg, respectively, and *high* if their SBP was ≥ 140 mm Hg and/or the DBP was ≥ 90 mm Hg, or if their readings were normal but they were under treatment for raised blood pressure in the past two weeks. In addition, *high risk* levels of SBP ≥ 160 mm Hg and/or DBP ≥ 100 mm Hg were also determined among participants to assess a higher probability or risk of hypertensive disorder.

Survey participants were also asked whether they were under medication for high blood pressure during the previous two weeks, as prescribed by a physician or other health professional. Respondents with treated and/or controlled raised blood pressure among those with raised blood pressure (SBP ≥140 and/or DBP ≥ 90 mmHg) or currently taking medication for raised blood pressure were further categorized to determine treatment success, treatment failure or being undetected and untreated, as follows:

- Under medication and controlled (treatment success) = those taking medication and having SBP <140 mmHg and DBP <90 mmHg;
- Under medication and uncontrolled (treatment failure) = those taking medication and having SBP ≥140 mmHg and/or DBP ≥90 mmHg;
- Undetected and uncontrolled (health system failure) = those not taking medication and having SBP ≥140 mmHg and/or DBP ≥90 mmHg**.**

For peer review only - http://bmjopen.bmj.com/site/about/guidelines.xhtml

1.5.3.2 Body mass index (BMI)

The height and weight of participants was taken to estimate their body mass index (BMI) as the ratio of weight/height2 (at the nearest decimal kilogram and decimal centimetre, respectively).

Measurements were carried out while standing with the heels together, feet apart, arms at sides and chin parallel to the floor on a flat, horizontal and firm surface (like tile, cement or wooden floor). Standardized and calibrated SECA® 813 digital floor scales and 213 portable stadiometers were used for weight and height measurements, respectively. To measure height and weight more precisely, participants were asked to follow standard procedures, including removal of their shoes and any bulky or heavy clothing to avoid overestimations.

Once BMI ratios determined, sample population was categorized according to the following WHO recommendations: underweight if BMI < 18.5, normal weight if BMI was between 18.5 - 24.9, overweight if BMI was between 25.0 - 29.9, and obesity if BMI was ≥ 30.

Average population BMI levels and proportion distribution among the sample population groups were determined.

1.5.3.3 Waist and hip circumferences

Waist circumference and hip circumference and their ratio were also assessed as other measures of obesity, in particular of central obesity.

Waist and hip circumference measurements were made while a participant remained standing, with feet together and hands on each side of the body, with a non-stretch Seca 201 measuring tape with millimetre precision. Waist circumference was measured by placing a tape measure around the bare abdomen at the midpoint between the lower margin of the last palpable rib and the top of iliac crest (hip bone). Hip circumference was measured by placing a tape measure around the maximum circumference over the buttocks.

The WHO cut-off points of waist circumference that determine waist obesity and categorized risk of metabolic complications and CVD are different for men and women. The waist–hip ratio (WHR) was computed among all respondents, excluding pregnant women.

1.6 Data collection

1.6.1 Staff recruitment

Interviewers and field supervisors in each governorate were recruited from among staff working in the Ministry of Health.

For peer review only - http://bmjopen.bmj.com/site/about/guidelines.xhtml

Overall, 66 data collectors and 11 field supervisors were recruited to participate in the training of data collection along with 1 regional coordinator, 1 IT technician and 1 laboratory technician in each governorate. Data collectors were mainly nurses and health educators nominated for data collection and for measurement of height, weight, waist and hip circumference, blood pressure, blood glucose, lipid profile, and vision testing. Urine samples were measured by the laboratory technicians in the health centre laboratories.

1.6.2 Data collection procedures

Field operations were carried out in the governorates during a four-month period in 2017, with the survey period chosen appropriately to avoid Ramadan/Eid periods. A media and advocacy action plan was implemented to raise awareness of the population about the survey, including disseminating information through leaflets, posters, press releases, radio and TV broadcasting, community and local Ministry of Health (MoH) staff participation. In addition to this focused publicity campaign, official identity cards to the field staff issued by MoH were of great help to secure sufficient recognition, cooperation and good responses for the interview in most cases. The respective authorities were also requested to provide the necessary assistance and co-operation to the field staff.

The interviews were conducted in all the governorates. The supervisors approached the selected households in each cluster, explained the aim and objectives of the survey, and sought their consent to participate in the survey. After recording the eligible members within a selected household, one participant was randomly selected from eligible members by the Android tablet. Each interview took place in a secure setting with adequate privacy at the household level. Each participant was interviewed at his/her household. As biomedical tests require 12 hours of fasting, appointments were given based on agreement between the interviewers and the respondents. Interviewers also explained the protocol for 24-hour urine collection to the respondent, obtained informed consent and provided the record sheet on which participants note the start and finish times of their 24-hour urine collection and any missed urine collections in the container.

1.6.3 Training for supervisors and data collectors

A one-week training program was conducted by the central research team from the Centre of Studies & Research in collaboration with WHO experts. This training program, which was held in Muscat in December 2016, included the survey objectives and field work staff duties, how to fill in the questionnaire in a polite, motivating and persuasive manner, how to understand the content of the questions if needed to clarify to respondents, how to enter the data and navigate

For peer review only - http://bmjopen.bmj.com/site/about/guidelines.xhtml

the Android tablet, how to ask for written consent and organize data collection and protection of confidentiality of the informant (consent, ID barcode labels, patterns of verbal and nonverbal behaviour), how to perform physical measurements and take biomarkers' samples using equipment applying standardized methodology, and role playing and mock interviews.

The field supervisors were given special instructions afterwards on coordinating process. Trainees who failed to show interest in the survey and those who did not attend the training program on a regular basis were not selected for the fieldwork and were replaced by other staff from the same Governorate after training.

1.6.4 Pilot Study

The pilot testing of the survey implementation process was implemented for 2 days (using Android tablets) selecting about 100 households which included males and females as well as Omani and non-Omani households. The pilot was also used to train the key survey personnel, test all survey materials prior to full implementation (skip errors, translation errors, awkward wording, and inadequate response categories), check quality of data collected. Lessons learned, logistic issues, and challenges identified were considered which maximized opportunities for improving the quality in the full survey implementation.

1.7 Data Management

1.7.1 Data collection

Each participant was allocated an identifier code (PID). The PID code consisted of seven digits the first two digits were the device number; the next three digits were the house listing number in the cluster and the last two digits were the person number in the household.

Data from STEP 1 and STEP 2 were submitted electronically online to the server from a household with identification of individual PID, household geographical location, including cluster with individual PID, day and time of completion. This was done either daily or at least once a week. After being analysed, blood and urine samples' results (STEP 3) were uploaded to the server and merged into the unified dataset, following conversion into SPSS and Microsoft Excel format in a single file.

1.7.2 Data validation

The central team (at the Centre of Studies & Research) downloaded data daily from server for data cleaning and management over a period of 6 months. Data management included continuously monitoring data collection, uploading and consolidation processes in the field,

For peer review only - http://bmjopen.bmj.com/site/about/guidelines.xhtml

validating quality of the data, creating weights, removing inconsistencies, namely “jump” errors/outliers, absence of data, excess data and invalid data. Moreover, to increase reliability of the collected data, verification of data in field was organized among 500 randomly selected households from all governorates. Accuracy of recording categorical and continued variables was checked using range and logic functions. The team also provided advice on software support and reported any problems or interview errors to the data collection field supervisors.

1.7.3 Data analysis

Weightage and Adjustment for sampling variation

Survey data analyses have to take into account whether the results are representative of the sample alone (unweighted analysis) or of the entire target population (weighted analysis). Since the primary objective was to be able to determine the estimates for the whole country, a weighted analysis was considered necessary. Weights adjusting for this complex survey design were required to decrease the risk of biases resulting from diverse factors. The sample weight is comprised of the inverse probability of selection. The household weights took into account the selection probability of the clusters within each stratum and the size (the number of households) of the cluster. The sample weight was also adjusted for non-response at the household level. The individual weight assumed that adults in the same cluster were selected by simple random sampling but the calculation scheme did not take into account the household size. This approach could have biased any key indicators, which was strongly associated with the household size. The individual weight was also adjusted for non-response.

Means, medians, proportions, standard errors, and 95% confidence intervals (95% CI) values were calculated to estimate central and dispersion measures and used to assess prevalence differences of NCD risk factors. Statistical procedures for data calculation and analyses were performed through two programs: EpiInfo in collaboration with WHO, and IBM SPSS (Version 20). All the figures and indicators in the tables were calculated using SPSS complex samples analysis. The figures presented as footnote (with an asterism) under each table were calculated after using population proportion weight. To allow for international comparisons of Oman survey results, age- and sex-adjusted overall values were calculated for all indicators using the direct method and the WHO standard population. Values are presented as footnotes on the tables to limit confusion with the national unadjusted data. It should also be noted that the estimates shown for governorates in the tables should be treated with caution as they represent the respondents in the respective governorate, and not the governorate itself.

For peer review only - http://bmjopen.bmj.com/site/about/guidelines.xhtml

1.8 Ethical considerations

Two informed consent forms, one for filling in the questionnaire and performing physical measurements (e.g. STEP 1 and STEP 2) and another for taking blood and urine samples for biomarkers (STEP 3), were requested to be signed by each participant. To enhance participation, an information letter was sent to all selected households in advance of data collection, identifying purpose, benefits and the voluntary participation in the survey.

To guarantee the high level of confidentiality and data security, every eligible subject was granted a unique identification number which was used for any reference from the register, with the exception of providing a personal feedback to a particular eligible subject for medical reasons.

Prior to its implementation, the survey was approved by the Research and Ethical Review & Approval Committee (RERAC) of the Ministry of Health.

For peer review only

Table S1. Comparison of the general characteristics of the study participants with those of the national sample of the 2017 WHO STEPS Survey carried out in Oman.

	**Salt and Potassium Survey mean (SD)**			**2017 WHO STEPS National Survey † mean (95% CI)**		
	**All (n=569)**	**Men (n=193)**	**Women (n=376)**	**All (n=6,582)**	**Men (n=3,365)**	**Women (n=3,217)**
Age (yrs)	39.4 (13.1)	38.7 (14.3)	39.8 (12.5)	38.2 (37.4-39.0)	38.0 (37.1-38.8)	38.6 (37.3-39.8)
Height (cm)	159.4 (11.2)	167.9 (9.7)	154.9 (9.2)	-	167.4 (166.6-168.2)	156.1 (155.5-156.6)
Weight (kg)	74.9 (21.5)	81.4 (22.5)	71.4 (20.1)	-	74.9 (73.6-76.2)	68.1 (66.6-69.7)
BMI (kg/m^2)	29.3 (7.2)	28.9 (7.6)	29.5 (7.0)	27.3 (26.9-27.6)	26.7 (26.3-27.2)	27.9 (27.3-28.5)
Waist circ. (cm)	93.8 (15.7)	95.0 (15.7)	93.2 (15.7)	-	90.0 (89.0-91.0)	87.6 (86.1-89.1)
Hip circ. (cm)	104.5 (15.0)	102.6 (13.7)	105.5 (15.6)	-	101.2 (98.8-103.5)	102.5 (99.2-105.7)
Systolic BP (mmHg)	125.9 (18.2)	134.0 (17.0)	121.7 (17.3)	124.9 (124.0-125.7)	130.3 (129.2-131.4)	119.1 (117.7-120.4)
Diastolic BP (mmHg)	80.9 (10.7)	83.4 (11.5)	79.7 (10.1)	79.5 (78.9-80.1)	81.5 (80.6-82.5)	77.4 (76.7-78.1)
Pulse rate (b/min)	79.8 (10.5)	78.5 (11.8)	80.4 (9.8)	79.4 (78.7-80.2)	77.7 (76.4-78.9)	81.3 (80.6-82.0)
Hypertension (%)	27.4	38.5	21.7	27.5	33.1	21.5

†https://www.who.int/ncds/surveillance/steps/Oman_STEPS_2017_Data_Book.pdf?ua=1

For peer review only

Table S2. Characteristics of excluded participants and comparison with those included in the final analysis.

Variable	Included (n=569)	Excluded (n=159)	P value*
Age (years)	39.4 (13.1)	37.5 (13.9)	0.037
Height (cm)	159.4 (11.2)	158.7 (8.6)	>0.05
Weight (kg)	74.9 (21.5)	73.4 (18.3)	>0.05
BMI (kg/m^2)	29.3 (7.2)	29.1 (7.0)	>0.05
Waist circumference (cm)	93.8 (15.7)	92.3 (17.9)	>0.05
Hip circumference (cm)	104.5 (15.0)	102.6 (16.4)	>0.05
Systolic blood pressure (mm Hg)	125.9 (18.2)	124.2 (18.8)	>0.05
Diastolic blood pressure (mm Hg)	80.9 (10.7)	79.1 (11.6)	0.025
Pulse rate (b/min)	79.8 (10.5)	80.9 (10.5)	>0.05
Hypertension (%)	27.4	23.9	

Results are mean (SD), *by Mann-Whitney U-test
Hypertension: SBP/DBP >=140/90 mmHg

For peer review only

For peer review only - http://bmjopen.bmj.com/site/about/guidelines.xhtml

STROBE Statement—Checklist of items that should be included in reports of ***cross-sectional studies***

	Item No	Recommendation	Page No
**Title and abstract**	1	(*a*) Indicate the study's design with a commonly used term in the title or the abstract	1,2
		(*b*) Provide in the abstract an informative and balanced summary of what was done and what was found	2
**Introduction**			
Background/rationale	2	Explain the scientific background and rationale for the investigation being reported	4
Objectives	3	State specific objectives, including any prespecified hypotheses	5
**Methods**			
Study design	4	Present key elements of study design early in the paper	5-6
Setting	5	Describe the setting, locations, and relevant dates, including periods of recruitment, exposure, follow-up, and data collection	5-7
Participants	6	(*a*) Give the eligibility criteria, and the sources and methods of selection of participants	5-7
Variables	7	Clearly define all outcomes, exposures, predictors, potential confounders, and effect modifiers. Give diagnostic criteria, if applicable	5-7
Data sources/ measurement	8*	For each variable of interest, give sources of data and details of methods of assessment (measurement). Describe comparability of assessment methods if there is more than one group	5-7
Bias	9	Describe any efforts to address potential sources of bias	5-7
Study size	10	Explain how the study size was arrived at	7
Quantitative variables	11	Explain how quantitative variables were handled in the analyses. If applicable, describe which groupings were chosen and why	7
Statistical methods	12	(*a*) Describe all statistical methods, including those used to control for confounding	7
		(*b*) Describe any methods used to examine subgroups and interactions	N/A
		(*c*) Explain how missing data were addressed	7
		(*d*) If applicable, describe analytical methods taking account of sampling strategy	7
		(*e*) Describe any sensitivity analyses	Suppl Material
**Results**			
Participants	13*	(a) Report numbers of individuals at each stage of study—eg numbers potentially eligible, examined for eligibility, confirmed eligible, included in the study, completing follow-up, and analysed	8-9
		(b) Give reasons for non-participation at each stage	8-9
		(c) Consider use of a flow diagram	Figure 2
Descriptive data	14*	(a) Give characteristics of study participants (eg demographic, clinical, social) and information on exposures and potential confounders	8, Tab 1, 2

For peer review only

		(b) Indicate number of participants with missing data for each variable of interest	Suppl.material
Outcome data	15*	Report numbers of outcome events or summary measures	Tab 2, 3, 4, 5
Main results	16	(*a*) Give unadjusted estimates and, if applicable, confounder-adjusted estimates and their precision (eg, 95% confidence interval). Make clear which confounders were adjusted for and why they were included	7-9<br>Tab 2-5
		(*b*) Report category boundaries when continuous variables were categorized	Tab 2-3
		(*c*) If relevant, consider translating estimates of relative risk into absolute risk for a meaningful time period	N/A
Other analyses	17	Report other analyses done—eg analyses of subgroups and interactions, and sensitivity analyses	Suppl. material
**Discussion**			
Key results	18	Summarise key results with reference to study objectives	9
Limitations	19	Discuss limitations of the study, taking into account sources of potential bias or imprecision. Discuss both direction and magnitude of any potential bias	10-11
Interpretation	20	Give a cautious overall interpretation of results considering objectives, limitations, multiplicity of analyses, results from similar studies, and other relevant evidence	10-12
Generalisability	21	Discuss the generalisability (external validity) of the study results	10-12
**Other information**			
Funding	22	Give the source of funding and the role of the funders for the present study and, if applicable, for the original study on which the present article is based	3

*Give information separately for exposed and unexposed groups.

Note: An Explanation and Elaboration article discusses each checklist item and gives methodological background and published examples of transparent reporting. The STROBE checklist is best used in conjunction with this article (freely available on the Web sites of PLoS Medicine at http://www.plosmedicine.org/, Annals of Internal Medicine at http://www.annals.org/, and Epidemiology at http://www.epidem.com/). Information on the STROBE Initiative is available at www.strobe-statement.org.

For peer review only - http://bmjopen.bmj.com/site/about/guidelines.xhtml

BMJ Open

A national survey to estimate sodium and potassium intake, and knowledge attitudes and behaviours towards salt consumption of adults in the Sultanate of Oman.

Journal:	*BMJ Open*
Manuscript ID	bmjopen-2020-037012.R2
Article Type:	Original research
Date Submitted by the Author:	28-Jul-2020
Complete List of Authors:	Al-Mawali, Adhra; Ministry of Health Oman, Centre of Studies & Research<br>D'Elia, L; Federico II University Hospital, Department of Clinical Medicine and Surgery<br>Jayapal, Sathish; Ministry of Health Oman, Centre of Studies & Research<br>Morsi, Magdi; Ministry of Health Oman, Centre of Studies & Research<br>Al-Shekaili, Waleed ; Ministry of Health Oman, Centre of Studies & Research<br>Pinto, Avinash; Ministry of Health Oman, Centre of Studies & Research<br>Al-Kharusi, Hilal ; Ministry of Health Oman, Centre of Studies & Research<br>Al-Balushi, Zainab; Ministry of Health Oman, Centre of Studies & Research<br>Idikula, John; Ministry of Health Oman, Centre of Studies & Research<br>Al Harrasi, Ayaman; Ministry of Health Oman, Centre of Studies & Research<br>Cappuccio, Francesco Paolo; University of Warwick, Warwick Medical School
**Primary Subject Heading**:	Epidemiology
Secondary Subject Heading:	Nutrition and metabolism, Public health
Keywords:	EPIDEMIOLOGY, NUTRITION & DIETETICS, PUBLIC HEALTH

SCHOLARONE™
Manuscripts

I, the Submitting Author has the right to grant and does grant on behalf of all authors of the Work (as defined in the below author licence), an exclusive licence and/or a non-exclusive licence for contributions from authors who are: i) UK Crown employees; ii) where BMJ has agreed a CC-BY licence shall apply, and/or iii) in accordance with the terms applicable for US Federal Government officers or employees acting as part of their official duties; on a worldwide, perpetual, irrevocable, royalty-free basis to BMJ Publishing Group Ltd ("BMJ") its licensees and where the relevant Journal is co-owned by BMJ to the co-owners of the Journal, to publish the Work in this journal and any other BMJ products and to exploit all rights, as set out in our licence.

The Submitting Author accepts and understands that any supply made under these terms is made by BMJ to the Submitting Author unless you are acting as an employee on behalf of your employer or a postgraduate student of an affiliated institution which is paying any applicable article publishing charge ("APC") for Open Access articles. Where the Submitting Author wishes to make the Work available on an Open Access basis (and intends to pay the relevant APC), the terms of reuse of such Open Access shall be governed by a Creative Commons licence – details of these licences and which Creative Commons licence will apply to this Work are set out in our licence referred to above.

Other than as permitted in any relevant BMJ Author's Self Archiving Policies, I confirm this Work has not been accepted for publication elsewhere, is not being considered for publication elsewhere and does not duplicate material already published. I confirm all authors consent to publication of this Work and authorise the granting of this licence.

For peer review only

28 Jul 2020 – v13- R4

A national survey to estimate sodium and potassium intake, and knowledge attitudes and behaviours towards salt consumption of adults in the Sultanate of Oman.

Adhra Al-Mawali[1,2], Lanfranco D'Elia[3,4], Sathish Kumar Jayapal[1], Magdi Morsi[1], Waleed Nasser Al-Shekaili[1], Avinash D Pinto[1], Hilal Al-Kharusi[1], Zainab Al-Balushi[1], John Idikula[1], Ayaman Al-Harrasi[1] and Francesco P Cappuccio[3,5,6].

[1]Centre of Studies & Research, Ministry of Health, Muscat, Sultanate of Oman
[2] The Research Council, Sultanate of Oman
[3]WHO Collaborating Centre for Nutrition, University of Warwick, Coventry, UK
[4]Department of Clinical Medicine and Surgery, "Federico II" University of Naples Medical School, Naples, Italy.
[5]Division of Health Sciences, Warwick Medical School, University of Warwick, Coventry, UK
[6]University Hospitals Coventry & Warwickshire NHS Trust, Coventry, UK

Keywords: Sultanate of Oman; salt; sodium; potassium; population.
Short title: Sodium and potassium intake in Oman
Word count: 4,416

Correspondence to:

Prof. Francesco P Cappuccio
WHO Collaborating Centre for Nutrition
University of Warwick, Warwick Medical School, Division of Health Sciences
Gibbet Hill Road, Coventry CV4 7AL, UK
f.p.cappuccio@warwick.ac.uk; Tel.: +442476573129

For peer review only - http://bmjopen.bmj.com/site/about/guidelines.xhtml

ABSTRACT

Objectives. To estimate population sodium and potassium intakes, and explore knowledge, attitudes and behaviour (KAB) towards the use of salt in adults in the Sultanate of Oman.

Design. National cross-sectional population-based survey.

Setting. Proportional random samples, representative of Omani adults (18 years or older), were obtained from all Governorates of the Sultanate of Oman.

Participants. Five hundred and sixty-nine (193 men, 376 women; 18 years or older) were included in the analysis (response rate 57%). Mean age was 39.4 years (SD 13.1). Participants attended a screening including demographic, anthropometric and physical measurements.

Primary and secondary outcome measures. We assessed dietary sodium, potassium and creatinine by 24h urinary sodium (UNa), potassium (UK) and creatinine (UCr) excretions. We collected KAB by a questionnaire on an electronic tablet.

Results. Mean UNa was 144.3 (78.8) mmoL/day, equivalent to 9.0 g of salt/day and potassium excretion 52.6 (32.6) mmoL/day, equivalent to 2.36 g/day, after adjusting for non-urinary losses. Men ate significantly more sodium and potassium than women. Only 22% of the sample had a salt intake below the World Health Organization (WHO) recommended target of 5 g/day and less than 10% met WHO targets for potassium excretion (>90 mmoL/day). Whilst 89.1% of those interviewed knew that consuming too much salt could cause serious health problems and only 6.9% felt they were using too much added salt, 1 in 2 participants used always or often salt, salty seasonings or salty sauces in cooking or when preparing food at home.

Conclusions. In the Sultanate of Oman, salt consumption is higher, and potassium consumption lower, than recommended by WHO, both in men and in women. The present data provides, for the first time, evidence to support a national programme of population salt reduction to prevent the increasing burden of CVD in the area.

For peer review only - http://bmjopen.bmj.com/site/about/guidelines.xhtml

Article summary

Strengths and Limitations of the study

- National survey of Omani men and women using 24h urine collections.
- Adoption of quality control process to minimize the use of incomplete urine collections.
- Overall response rate was 57%, comparable with other similar population surveys.
- Non-responders did not differ in their baseline characteristics from responders.
- We cannot rule out the risk of selection bias.

For peer review only - http://bmjopen.bmj.com/site/about/guidelines.xhtml

28 Jul 2020 – v13- R4

INTRODUCTION

Non-communicable diseases (NCDs) are the leading, yet preventable, causes of death worldwide[1]. The reduction of its burden is now a global health priority of the United Nations[2], endorsed by the World Health Organization (WHO) Action Plan that has identified a set of cost-effective policy options ('best buys'), of which reduction in population salt consumption is one[3].

In the Sultanate of Oman, NCDs are amongst the leading causes of death, accounting for 72% of all deaths[4]. Cardiovascular disease (CVD) represents an increasingly common cause of population morbidity and mortality, accounting for 36% of all deaths[4]. It represents a major public health challenge undermining socio-economic development[5].

High blood pressure (BP) and unhealthy diets are the leading risk factors for CVD in the world[1]. Raised BP is a determinant of the CVD risk in the Sultanate of Oman, where the prevalence of raised blood pressure in people aged 18 years or older is 33%, higher in men (39%) than in women (27%)[5-6].

High salt (i.e., sodium chloride, 1 g = 17.1 mmoL of sodium) consumption is an important determinant of high BP. A high salt intake is associated with raised BP that leads to increased risk of vascular diseases[7-10]. In addition, high salt intake is related to adverse health effects independent of its effects on BP[11-13]. A moderate reduction in salt consumption reduces BP[7-8] and it can improve the health outcomes and indirectly reduce the overall mortality through beneficial effect on the BP[9-10].

The World Health Organization (WHO) recommends that adults should consume no more than 5 g of salt daily[14]. However, mean daily intakes of salt in most of the countries in the world exceed this recommendation[15-16]. Whilst there is no definitive estimate of population dietary salt intake in the Sultanate of Oman, average consumption could be high, similar to some countries in the sub-region[17-18]. In the Sultanate of Oman it is a common habit to add salt and salty condiments to food at the table and whilst cooking. Also, the habit of eating out is increasing (especially in urban areas) which leads to an increased salt intake, since restaurants tend to use higher amounts of salt to render food tastier. Our study was designed to support the salt reduction strategy of the Eastern Mediterranean region, including the Sultanate of Oman, in which monitoring population salt consumption is one of the three pillars[19]. Current national initiatives include establishment of a multi-sectoral national committee, legislation on salt reduction, development of salt content benchmarks, dietary guidelines[18]. The "Health Vision 2050" for the Sultanate of Oman was also developed as a roadmap by analysing extensively the status of the Omani health system, the morbidity and mortality in the population, the challenges facing the health

For peer review only - http://bmjopen.bmj.com/site/about/guidelines.xhtml

system, the expected future developments and changes in the population including macro-social and macro-economic changes in order to augment the performance of the health system.

In contrast to sodium, epidemiological and intervention studies suggest beneficial effects of dietary potassium on BP and cardiovascular health[20-22]. The Sultanate of Oman lacks data on actual potassium consumption. The WHO currently recommends that adults should consume not less than 90 mmoL of potassium daily[23]. Hence, we need reliable data on sodium and potassium intake in the Sultanate of Oman.

The primary aim of the present study was to establish current baseline average consumption of sodium and potassium by 24h urine collection, in a national random sample of Omani men and women. The study also aimed to explore knowledge, attitudes and behaviour towards dietary salt.

MATERIAL AND METHODS

Participants and Recruitment

We nested the salt survey within the main Oman NCD survey of 6,833 households (Supplementary material, Text S1). We recruited only one member per household. We designed the salt survey to collect 24h urinary samples from a subgroup of at least 90 participants from each governorate (region). The survey included Omani citizens only. We included a total of 999 randomly selected Omani men and women. They were all aged 18 years or older. They comprised residents of all governorates of the Sultanate of Oman (Table 1). The sample was representative of the national sample for its general characteristics (Supplementary material, Table S1).

From the sampling frame and according to the EMRO-WHO Protocol[24], we excluded the following groups: people unable to provide informed consent, those with known history of heart or kidney failure, stroke, liver disease, those who recently began therapy with diuretics (less than two weeks), pregnant women, any other conditions that would make 24h urine collection difficult. To detect approximately 1 g reduction in salt intake over time using 24h urinary sodium excretion (difference ~20 mmoL/24h), with a standard deviation of 75 mmoL/day (alpha = 0.05, power = 0.80), a minimum sample of 120 individuals per stratum is recommended[24]. Thus, we estimated a minimum recommended sample size of 240 per age and sex groups and adjusted for an anticipated non-response rate of 50%[24]. We stratified the population in groups by sex (men and women). Therefore 480 individuals were originally needed to be selected (total n=120*2 groups/0.5 attrition=480).

The survey took place between December 2017 and May 2018. From the 999 individuals interviewed in the sampling frame, 569 of them (57.0%) provided suitable data for inclusion in the survey analysis. The general characteristics of the included participants did not differ substantially from those of the excluded participants (Supplementary material, Table S2). Originally, 262 (26.0%) did not provide complete urine collections (either declaring missing more than one void or providing collections <23h or above 25h), 87 (8.7%) had missing data, 48 (4.8%) provided urine collections with volume less than 500 mL (conventionally taken as not plausible), and 24 (2.4%) had urinary creatinine excretion outside 2 standard deviations (SDs) of the sex-specific distribution of urinary creatinine in the sample (Figure 1).

Ethical considerations

We carried out the survey in accordance with the Declaration of Helsinki and Good Clinical Practice[25]. We obtained ethical approval for the survey from the Research and Ethics Review and Approval Committee (RERAC) of the Ministry of Health of the Sultanate of Oman and participants provided written informed consent to take part.

Patient and Public Involvement

No patient involved

Data Collection

We performed the examination in a quiet and comfortable room, with the participants who did not smoke, exercise, eat, and consume caffeine before attending, and had been instructed to present with a full bladder 30 minutes before measurements to reduce the risk of underestimating the urine collection. We carried out the survey in three steps: a) questionnaire survey, b) physical measurements and c) 24h urine collections.

We based the questionnaire on the Core and Expanded version of the WHO STEPS Instrument for Chronic Disease Risk Factor Surveillance (version 3.0)[26] and country-specific requirements. It contained 11 Core, 1 Optional and 4 country-specific modules which included a total of 420 questions, to determine socio-demographic characteristics of participants, key behavioural risk factors (tobacco use, harmful alcohol consumption, diet with frequency of fruit and vegetable consumption, high dietary salt consumption, oil and fat use, physical inactivity), knowledge attitudes and behaviour on dietary salt, given lifestyle advises, and additional health-related information not presented here. Knowledge, attitude and behaviours toward the consumption of salt was assessed by asking participants about the frequency, quantity, and type of salt used in the household, as well as their cooking habits and

For peer review only - http://bmjopen.bmj.com/site/about/guidelines.xhtml

their attitudes and perceptions towards dietary salt intake. Processed food was defined, per WHO STEPS protocol, as foods altered from their natural state, such as packaged salty snacks, canned salty food, cheese, and processed meat along with country-specific pictorial show cards.

We measured anthropometric indices, BP and heart rate in all participants. Height was in cm and body weight in kg using a Standardized and calibrated SECA®813 digital floor scales and 213 portable stadiometers, respectively. Body mass index (BMI) was calculated as weight (kg) divided by height squared (m^2). Waist and hip circumferences were measured by a non-stretch SECA®201 measuring tape to the nearest mm[24]. We took systolic and diastolic BP and heart rate measurements three times in the right arm on a sitting position, using an appropriate cuff and a validated digital device (OMRON M3). We ignored the first measurement, and used the mean of second and third measurements for analysis. We took measurements after the participant had rested for 15 minutes and each with three minutes of rest between the measurements (maximum deviation of cuff pressure measurement ± 3 mmHg and of pulse rate display ± 5%). Hypertension is defined as systolic and/or diastolic BP ≥ 140/90 mmHg or regular antihypertensive treatment[27]. We obtained a single 24h urine collection from the participants. We gave each participant a leaflet with explanations along with the necessary equipment and a record sheet on which participant noted the start and the finish times of their urine collection, any missed urine aliquots and any medication taken during the collection. We instructed the participants carefully on urine collection methodology[24]. In an effort to minimize bias, we also requested participants not to change their diet before or during the day of the urine collection. They discarded the first void upon waking on the day of collection. Participants then filled the 24-hour urine container over the 24-hour period. On the following day, the field team-members visited the household, measured total volume, mixed it thoroughly, and obtained a urine sample, which was kept in a cool-box for transport to the respective laboratory. On arrival at the laboratory, we either carried out sodium, potassium and creatinine determinations immediately or stored samples in the fridge until the determination (as soon as possible). Sodium and potassium concentration in the urine samples were determined using an Ion Selective Electrode with an Abott C8000 & Roche Cobas 6000 and expressed in mmol/L[28]. Creatinine concentration was determined through either the kinetic (Abbott C8000) or enzymatic (Roche Cobas 6000) method and expressed in mg/dL[29]. These determinations were carried out in one reference laboratory in each of the 11 governorates, except for 2 regions (Dhofar and Musandam) which had 2 receiving reference laboratories each. All laboratories underwent joint quality control.

For peer review only - http://bmjopen.bmj.com/site/about/guidelines.xhtml

Statistical Analysis

We performed all statistical analyses using the SPSS software, version 20 (SPSS Inc., Chicago, IL, USA). We used T-test for unpaired samples to assess differences between group means and Pearson chi-square test to test the association between categorical variables. To convert urinary output into dietary intake, we first converted the urinary excretion of sodium (UNa) or potassium (UK) values (mmoL/day) into mg/day (for sodium 1 mmol = 23 mg of sodium, for potassium 1 mmol = 39 mg). We then multiplied the sodium value by 2.542 to convert dietary sodium (Na) intake into salt (NaCl) intake. We finally multiplied sodium values by 1.05 (assuming that approximately 95% of sodium ingested is excreted)[30]. We calculated potassium dietary intake assuming that 85% of the potassium ingested is excreted in the urine[31]. The results were reported as mean (SD), median (IQ range) or as percentages, as appropriate. We considered two-sided p below 0.05 as statistically significant.

RESULTS

The final population sample included 569 participants between 18 and 69 years old (n = 193 or 34% men and n = 376 or 66% women), recruited nationally.

Characteristics of the Participants

Table 2 shows the characteristics of the participants. There was no statistically significant difference in the mean age and in body mass index between men and women, however men had significantly higher BP and slower heart rate than women did. The prevalence of hypertension was on average 27.4%, significantly higher in men than in women (38.5% v 21.7%, p<0.001).

Daily Urinary Excretions of Volume, Sodium, Potassium and Creatinine and Salt and Potassium Intake

Average urinary volume excretion was 1354 mL per day, being higher in men than women (Table 3). Average urinary creatinine excretion was 1.33 g per day, being again higher in men than women (Table 3). Mean urinary sodium was 144.3 (SD 78.8) mmoL/24h, equivalent to a mean consumption of 9.0 (4.9) g of salt per day (Table 3). Men excreted more sodium than women did (mean difference 15.0 mmoL/24h, p < 0.05), equivalent to ~1.0 g of higher salt consumption than women did. Only 22% of the participants met the levels of salt intake of 5 g or less recommended by the WHO,

For peer review only - http://bmjopen.bmj.com/site/about/guidelines.xhtml

with no difference between sexes. Mean urinary potassium was 52.6 (32.6), equivalent to a mean consumption of 2.36 (1.46) g of potassium per day (Table 3).

Men excreted significantly more potassium than women; 9.1% of participants met the levels of potassium intake of 90 mmoL/day or more recommended by the WHO.

The sex difference in total daily salt and potassium intakes is almost entirely due to the fact that men eat more food than women, as they are taller and heavier, despite having comparable body mass index. This is a consistent finding across populations in different countries and from different continents.

Knowledge, Attitude, and Behaviours towards Salt Intake and other eating patterns.

Knowledge, attitude, and behaviours toward the consumption of salt are presented in Table 4. A total of 28.1% of respondents mentioned that they added salt or salty sauces always or often to food. The percentage of women who added salt or salty sauces always or often to their meal was significantly higher than that of men (30.8% vs 22.8%; p=0.005). A total of 47.0% of respondents reported that they always or often added salt, salty seasonings or sauces when cooking or preparing food at home, women more than men (48.1% vs 44.8%; p=0.04). More than 1 in 5 (22.3%) mentioned that they consumed processed foods high in salt always or often. Very few (6.9%), however, felt they consumed too much salt or salty sauces, although 89.1% knew that consuming too much salt could cause serious health problems. We also asked participants about dietary attitudes about the consumption of fruit and vegetables, oil or fats (Table 5). Interestingly, 68.0% consumed fruit at least five days a week, and 54.5% at least three servings on these days. Men appeared to report more fruit consumption than women did (40.4% vs 27.7%; p=0.002). Vegetables were also consumed frequently (75.9% at least 5 days a week), with 40.1% having at least 3 servings on one of those days (women more frequently than men). The majority (90.8%) used vegetable oil for meal preparation in the household and more than half (54.2%) consumed food prepared outside home at least once a week. Men were more likely than women to do so (67.9% vs 47.2%; p<0.001).

DISCUSSION

This the first nationally representative population-based survey carried out in the Sultanate of Oman assessing dietary sodium and potassium consumption in adult Omani men and women, using the gold standard measure of 24h urinary sodium and potassium excretions as a biomarker of intake. The results show that salt consumption is higher and potassium consumption is lower than recommended by the World

For peer review only - http://bmjopen.bmj.com/site/about/guidelines.xhtml

Health Organization[14,23], both in men and women. Average population salt consumption was 9.0 g per day, almost double the World Health Organization recommended maximum population target of 5 g per day[14]. Less than 1 in 4 participants met these targets. Salt consumption varied across Governorates, being the lowest in South Sharqiah (5.3 g per day) and the highest in Al-Dhahirah (14.8 g per day). Average population excretion of potassium was 53 mmol per day (equivalent to about 2.36 g per day), lower than the World Health Organization recommended maximum population target of >90 mmol per day, equivalent to approx. 3.90 g per day (assuming urinary potassium being 85% of the intake)[23]. Potassium consumption also varied across Governorates, being the lowest in Al-Wasta (1.41 g per day) and the highest in North Sharqiah (4.25 g per day). The urinary sodium-to-potassium ratio averaged 3.3, with no difference between men and women. Findings from the INTERSALT study showed that a difference in sodium-to-potassium ratio from 3.1 to 1.0 was associated with a 3.36 mmHg difference in population systolic BP[32-33]. The TOHP study reported a direct association between the urinary sodium-to-potassium ratio and CVD[10, 34-35]. Moreover, a unit difference in the ratio would be associated with a 13% reduction in total mortality[35]. Measuring the ratio is obviously important, although no evidence-based global guidelines have determined population targets, as yet.

Our questionnaire revealed that half of the population seen often used sauces and condiments (invariably containing high concentrations of salt) but only 10% believed this was too much. A quarter of the surveyed population added salt to food regularly, one in five ate processed food often and more than half of the population ate out at least once a week, with men more likely than women. These results, in addition to those obtained in previous surveys on unhealthy dietary habits, support the national Health Vision set by the Sultanate of Oman to reduce the burden of CVD[36]. This document sets the health visions for the country in 40 years. The comprehensive analyses of many factors affecting the population health and the healthcare system indicate that non-communicable diseases, in the context of increased life-expectancy and population ageing, pose a significant threat to the health of the Omani people and it identify the need to be able to respond to this challenge. Population salt reduction is one of the priorities.

Comparison with countries of the Gulf Cooperation Council (GCC) and of the Arab Peninsula.
In the Gulf Cooperation Council (GCC) countries, populations lead a sedentary lifestyle, both hypertension and obesity are common[17] and they are major contributors to NCDs[37]. The estimated total mortality in GCC countries attributable to NCDs vary

For peer review only - http://bmjopen.bmj.com/site/about/guidelines.xhtml

from 65% to 78%, with the highest estimates in Bahrain and Saudi Arabia and the lowest in Oman and Qatar, respectively[37]. Salt intake is deemed high in most countries of the Eastern Mediterranean Region (EMR), although there are only a few studies that directly measured population levels, with inconsistent results due to methodological inadequacies[17.18]. The Global Burden of Disease (GBD) estimates of average salt consumption using a Bayesian model suggest that salt consumption in GCC countries may vary from 8.0 g per day in Saudi Arabia to 13.5 g per day in Bahrain[38]. Estimates of salt intake in neighbouring countries would also range between 7.8 g per day in Lebanon and 10.3 g per day in Jordan[38]. The present study is one of the few nationally representative surveys in GCC countries using the gold standard method of assessment of dietary salt intake. Its results suggest an intake close to that estimated by the GBD. In addition to the GBD, however, our study also provides, for the first time, direct measures of average population potassium consumption, also targeted by WHO recommendations for cardiovascular prevention[20,23].

Many countries of the EMRO Region of WHO are developing and/or implementing national initiatives to decrease population salt intake[18]. National initiatives include the establishment of national multisectoral committees, the engagement of the government through regulatory measures and legislation (Bahrain, Iran, Jordan, Oman, Qatar), the specification of the food categories prioritized for action such as bread (Kuwait, Qatar) and canned foods (Iran), the development of national benchmarks and targets (Bahrain, Iran, Oman have), dietary guidelines (Afghanistan, Lebanon, Oman, Saudi Arabia), media awareness campaigns (Lebanon, United Arab Emirates), salt labelling, collaborative actions involving the food industry and/or restaurants and food caterers (Kuwait, Qatar, United Arab Emirates), and the monitoring and evaluation of sodium intakes and salt content of foods (Iran, Lebanon, Oman, Qatar).

Comparisons with studies in other countries.

National salt and potassium consumption surveys have been carried out in almost all regions of the world, especially in response to the recommendations from the World Health Organization that identified population salt reduction as one of the most cost-effective and feasible approaches to prevent non-communicable diseases[2-3]. Globally, there is a high variation in the readiness of countries to adopt and implement the different aspects of the overall strategy, with low-and-middle income countries still lagging behind[39-40]. Nevertheless, where surveys have been carried out to establish the size of the problem, average levels of salt intake have been very high in countries of Eastern Europe (10.8 g/day in Moldova and 11.6 g/day in Montenegro with potassium

For peer review only - http://bmjopen.bmj.com/site/about/guidelines.xhtml

about 30% lower than recommended and sodium-to-potassium ratios of 3.0 and 2.4, respectively)[41-42], Central Asia (17.2 and 18.8 g /day in two sites of Kazakhstan)[43], China (two-fold North-South gradient from 15.6 to 8.4 g/day and potassium abut 60% lower than recommended)[44] and Australasia (about 9.0 g/day weighted means in Australia and 11.7 g/day in the Fiji Islands)[45-46], indicating urgent need for population interventions. The same studies have invariably indicated lower than recommended potassium excretion and high sodium-to-potassium ratio. In this respect the average intake of sodium in Oman seems reassuring, as the achievement of the set targets appear more feasible that in other countries where intake currently still exceeds the 10 g/day. However, potassium consumption is nearly half of what is recommended[23], resulting in a high sodium-to-potassium ratio.

Strengths and Limitations

Our study has several strengths. First, it is population-based survey across the whole country. Second, it included all adults. Thirds, it included both men and women. These study characteristics would allow with greater confidence the extrapolation of results to the whole country population, rather than those conducted in selected groups including patients[47], young female University students[48] or children[49]. Fourth, it used the current preferred methodology for estimating salt consumption. Fifth, we applied a rigorous quality control protocol to ensure completeness of urine collections, and to minimize both under and over-estimations. Current recommendations suggest the use of single complete 24-hour urine samples, collected from a representative population sample to assess the population's current 24-hour dietary sodium ingestion[50]. The role of single spot or short duration timed urine collections in assessing population average sodium intake requires more research. Single or multiple spot or short duration timed urine collections are, on the other hand, not recommended for assessing an individual's sodium intake especially in relationship to health outcomes[50]. Twenty-four hour diet recall and diet records inaccurately measure dietary sodium intake in individuals compared with the gold standard 24 hours urinary excretion[51]. Furthermore, there is poor agreement between estimates of sodium intake from food-frequency questionnaires and 24-hour urine samples[52]. Sixth, it has measured directly the amount of potassium consumption, additional nutrient targeted for cardiovascular prevention[20,23]. Seventh, we standardised fieldwork and used standardised laboratory methodologies across the country. Eighth, all laboratories underwent joint quality control[25].

There are limitations too. First, we included only 57% of the urine samples originally collected from willing individuals. This was due to the stringent quality

28 Jul 2020 – v13- R4

control that has led to the exclusion of incomplete or erroneous collections[24]. This could have introduced a self-selection bias. The comparison of the baseline characteristics of the studies sample versus the excluded group suggests that the two groups were comparable for general characteristics, with the exception of the latter being two years younger and having a 1.8 mmHg lower diastolic blood pressure. Second, we assessed urinary sodium and potassium excretions only once. Whilst we cannot characterize an individual's intake in such a way[50], there is less likelihood of a bias of group estimates. Third, although we requested participants not to change their diet prior to urine collection, it would be difficult to rule out entirely any bias during collection. Fourth, although we administered a questionnaire to derive knowledge, attitudes and behaviours towards the use of salt, we were unable to establish the relative contribution of discretionary sources of salt and commonest foods contributing to salt as well as potassium consumption.

Potential impact

The population in the Sultanate of Oman is of just over 5 million (Ministry of Health Annual Health Report, 2018 estimates), of which about 2.3 million are Omani nationals[4] (surveyed in the present study). Approximately 51% are 25 years or older. To meet a 30% reduction in population salt consumption set by WHO by 2025, the Sultanate of Oman should aim at a 2.7 g per day salt reduction nationally. This reduction would avert 8.1% CVD deaths per year, and more non-fatal events and disabilities[2]. Additional benefits would be achieved if we increased at the same time population potassium intake towards WHO set targets, leading to a significant reduction in the sodium-to-potassium ratio in the diet. This could be achieved not only by increasing consumption of plant-based foods, but by enriching the diet with potassium-rich salt substitution in food manufacturing and processing or by using potassium-reach salts instead of sodium chloride, where sodium intake is predominantly originating from discretionary sources. Potassium-rich salts lower BP effectively[53] and the potential risk associated with potassium supplementation used at a population level[54] would be offset by a net reduction in CVD deaths[55].

Policy Implications

The Sultanate of Oman has embraced amongst its health priorities the prevention and control of noncommunicable diseases and improvement in nutrition[4] in line with the strategic directions of WHO endorsed by the Eastern Mediterranean Region (EMRO) in 2012 and 2013[18]. Since then several countries have conducted dietary assessment studies in an attempt to assess the population's salt and potassium intake[18]. Studies in

For peer review only - http://bmjopen.bmj.com/site/about/guidelines.xhtml

the area have also attempted to identify the major dietary contributors to sodium intake. Studies are still limited and there are large variations in dietary habits in the Region due to cultural, ethnic, religious and social heterogeneity. The commonest source of salt consumption across the Region is bread[18,56], in all its different forms, with other sources being more relevant in different countries. In Lebanon[57] and in Bahrain[58], dairy products are common sources, whilst in Morocco[59] major contributors to salt consumption include cereal-based products, spices and condiments and milk products. These indications, together with the awareness and behaviours measured, suggest that to reduce population salt consumption in the Sultanate of Oman the following initiatives should be taken: (a) improving salt-related knowledge through health promotion campaigns, (b) measuring major sources of salt consumption, (c) establishing collaborations with local authorities to reduce the amount of salt used in traditional bread making and locally produced condiments, (d) adopting a labelling strategy for imported foods with high salt content.. In addition, the Ministry of Health should develop strategies and methodologies to measure the indicators of population salt consumption[60].

Conclusions

This study demonstrates that salt consumption in the Sultanate of Oman is high and should be reduced through a public health action aiming at the entire population. Likewise, potassium consumption is particularly low. The KABs survey indicates areas of limited awareness. Education of the dangers of high salt consumption and where salt is hidden, of the benefits of increasing potassium through fruit, vegetables, nuts and legumes, alongside accurate labelling and marketing of food, surveillance to measure and monitor salt use, and reformulating bread are all important elements of an effective national salt reduction programme[18,19,61].

Supplementary materials: The following are available online at
Text S1: Material and Methods (full text). **Table S1**. Comparison of the general characteristics of the participants in the salt and potassium survey with those participating in the 2017 national WHO STEPS Survey. **Table S2.** Characteristics of the excluded participants and comparison with those included in the study.

Acknowledgments: We carried out the present analysis under the terms of reference of the WHO Collaborating Centre for Nutrition at the University of Warwick. The authors would like to express gratitude to the EMRO WHO Regional Office (Dr Ayoub Al-Jawaldeh) and the WHO Country Office of Oman (Dr Ruth Mabry) for facilitating

For peer review only - http://bmjopen.bmj.com/site/about/guidelines.xhtml

the study, the Department of Food Science and Human Nutrition of Sultan Qaboos University (Dr Lyutha Al-Subhi) and other members of the Ministry of Health (Dr Amel Ibrahim, Dr Ruqaya Balushi and Dr Salima for their helpful discussions in the preparation of the protocol. Special thanks go to the team of the Ministry of Health of the Sultanate of Oman involved in the preparation of the survey and data collection. Finally, we wish to express our gratitude to His Excellency the Undersecretary for Health Affairs of the Sultanate of Oman, Dr Mohammed bin Saif Al Hosni, and His Advisor, Dr Mahmood Shaban, for their advice, endorsements and support at a National level.

Author Contributions: FPC developed the study design and protocol, contributed to the analysis and drafted the manuscript, AA-M trained local teams, co-ordinated quality control and data collection. MM and LD carried out quality control and statistical analysis. AA-M, SKJ, WNA-S, ADP, HA-K, ZA-B, JI, AA-H coordinated the study, carried out the fieldwork and liaised with the local laboratory. MM helped with the drawing of the stratified random sample from the sampling frame. All authors contributed to the interpretation of the findings and they contributed significantly to the final version of the manuscript. FPC is the guarantor.

Competing interests: AA-M, SKJ, MM, WNA-S, ADP, HA-K, ZA-B, JI, AA-H are all staff of the Ministry of Health of the Sultanate of Oman. FPC is a technical advisor to the World Health Organization, unpaid member of Action on Salt and WASH. LD was a technical advisor to the World Health Organization and is a member of the Scientific Committee of the Italian Society of Human Nutrition.

Funding : The Ministry of Health of the Sultanate of Oman and the EMRO Regional Office of the World Health Organization supported the study.

Data Sharing Statement: No individual participant data will be available. Study protocol available in Supplementary Material. Any other data sharing proposal must be submitted in writing to the Ministry of Health of the Sultanate of Oman.

For peer review only - http://bmjopen.bmj.com/site/about/guidelines.xhtml

References

1. GBD 2015 Mortality and Causes of Death Collaborators. Global, regional, and national life expectancy, all-cause mortality, and cause-specific mortality for 249 causes of death, 1980–2015: A systematic analysis for the Global Burden of Disease Study 2015. *Lancet* 2016; 388: 1459–1544.
2. World Health Organization. Global Action Plan for the Prevention and Control of NCDs 2013–2020; World Health Organization: Geneva, Switzerland, 2013; pp. 1–103.
3. World Health Organization. Tackling NCDs: "Best buys" and other recommended interventions for the prevention and control of noncommunicable diseases; 2017. http://www.who.int/ncds/management/best-buys/en/
4. World Health Organization. Oman Health Profile 2018. World Health Organization. Regional Office for the Eastern Mediterranean, Cairo, Egypt, 2016; pp. 1-37. WHO-EM/HST/227/E
5. Al-Mawali A. Non-communicable diseases: shining a light on cardiovascular disease, Oman's biggest killer. *Oman Med J* 2015; 30(4): 227-228.
6. Al Riyami A, Elaty MAA, Morsi M, et al. Oman World Health Survey: Part 1: Methodology, sociodemographic profile and epidemiology of non-communicable diseases in Oman. *Oman Med J* 2012; 27(5): 425-443.
7. Aburto NJ, Ziolkovska A, Hooper L, Elliott P, Cappuccio FP, Meerpohl J. Effect of lower sodium intake on health: Systematic review and meta-analysis. *Br. Med. J.* 2013; 346: f1326.
8. He FJ, Li J, MacGregor GA. Effect of longer-term modest salt reduction on blood pressure: Cochrane systematic review and meta-analysis of randomised trials. *Br. Med. J.* 2013; 346: f1325.
9. Strazzullo P, D'Elia L, Kandala N.-B, Cappuccio FP. Salt intake, stroke, and cardiovascular disease: Meta-analysis of prospective studies. *Br. Med. J.* 2009; 339: b4567.
10. Cook NR, Cutler JA, Obarzanek E, et al. Long term effects of dietary sodium reduction on cardiovascular disease outcomes: Observational follow-up of the Trials of Hypertension Prevention (TOHP). *Br. Med. J.* 2007; 334: 885–888.
11. Cappuccio FP. Cardiovascular and other effects of salt consumption. *Kidney Int.* 2013; 3: 312–315.
12. D'Elia L, Rossi G, Schiano di Cola M, et al. Meta-analysis of the effect of dietary sodium restriction with or without concomitant renin–angiotensin–aldosterone

For peer review only - http://bmjopen.bmj.com/site/about/guidelines.xhtml

system-inhibiting treatment on albuminuria. *Clin. J. Am. Soc. Nephrol.* 2015; 10: 1542–1552.

13. D'Elia L, Galletti F, La Fata E, Sabino P, Strazzullo P. Effect of dietary sodium restriction on arterial stiffness: Systematic review and meta-analysis of the randomized controlled trials. *J. Hypertens.* 2018; 36: 734–743.
14. World Health Organization. Guideline. Sodium Intake for Adults and Children. World Health Organization (WHO): Geneva, Switzerland, 2012.
15. Brown IJ, Tzoulaki I, Candeias V, Elliott P. Salt intakes around the world: Implications for public health. *Int. J. Epidemiol.* 2009; 38: 791–813.
16. Cappuccio FP, Capewell S. Facts, issues, and controversies in salt reduction for the prevention of cardiovascular disease. *Funct. Food Rev.* 2015; 7: 41–61
17. Alhamad N, Almalt E, Alamir N, Subhakaran M. An overview of salt intake reduction efforts in the Gulf Cooperation Council countries. *Cardiovasc. Diagn. Ther.* 2015; 5(3): 172-177.
18. Al Jawaldeh A, Rafii B, Nasreddine L. Salt intake reduction strategies in the East Mediterranean Region. *East Mediterr. Health J.* 2018; 24(12): 1172-1180.
19. Cappuccio FP, Capewell S, Lincoln P, McPherson K. Policy options to reduce population salt intake. *Br. Med. J.* 2011; 343: 402–405.
20. Aburto NJ, Hanson S, Gutierrez H, Hooper L, Elliott P, Cappuccio FP. Effect of increased potassium intake on cardiovascular risk factors and disease: Systematic review and meta-analyses. *Br. Med. J.* 2013; 346: f1378.
21. D'Elia L, Barba G, Cappuccio, FP, Strazzullo P. Potassium Intake, Stroke, and Cardiovascular Disease. A meta-analysis of Prospective Studies. *J. Am. Coll. Cardiol.* 2011; 57: 1210–1219.
22. D'Elia L, Iannotta C, Sabino P, Ippolito R. Potassium rich-diet and risk of stroke: Updated meta-analysis. *Nutr. Metab. Cardiovasc. Dis.* 2014; 24: 585–587.
23. World Health Organization. Guideline: Potassium Intake for Adults and Children. World Health Organization (WHO): Geneva, Switzerland, 2012; pp. 1–52.
24. World Health Organization. How to Obtain Measures of Population-Level Sodium Intake in 24-Hour Urine Samples. World Health Organization. Regional Office of the Eastern Mediterranean, Cairo, Egypt, 2018; pp. 1–51. WHO-EM/NUT/279/E.
25. World Medical Association. Declaration of Helsinki. Recommendations guiding doctors in clinical research. *Bull. World Health Org.* 2008; 86: 650–651.
26. World Health Organization. The WHO STEPwise approach to chronic disease risk factor surveillance (STEPS). World Health Organization, Geneva,

For peer review only - http://bmjopen.bmj.com/site/about/guidelines.xhtml

28 Jul 2020 – v13- R4

Switzerland, 2014. https://www.who.int/ncds/surveillance/steps/STEPS_Instrument_v2.1.pdf

27. Mancia G, Fagard R, Narkiewicz K, et al. 2013 ESH/ESC Guidelines for the management of arterial hypertension: The Task Force for the management of arterial hypertension of the European Society of Hypertension (ESH) and of the European Society of Cardiology (ESC). *J. Hypertens.* 2013; 31: 1281–1357.
28. Oesch U, Ammann D, Simon W. Ion-selective membrane electrodes for clinical use. *Clin. Chem.* 1986; 32: 1448–1459.
29. Junge W, Wilke B, Halabi A, Klein G. Determination of reference intervals for serum creatinine, creatinine excretion and creatinine clearance with an enzymatic and a modified Jaffé method. *Clin. Chim. Acta* 2004; 344: 137–148.
30. Lucko AM, Doktorchik C, Woodward M, et al. Percentage of ingested sodium excreted in 24-hour urine collections: A systematic review and meta-analysis. *J Clin. Hypertens.* 2018; 20: 1220–1229.
31. Stamler J, Elliott P, Chan Q, for the INTERMAP Research group. INTERMAP Appendix tables. *J. Hum. Hypertens.* 2003; 17: 665–758.
32. Rose G, Stamler J, Stamler R, et al. INTERSALT: An international study of electrolyte excretion and blood pressure. Results for 24 h urinary sodium and potassium excretion. *Br Med J* 1988; 297: 319–332
33. Stamler J, Rose G, Stamler R, et al. INTERSALT study findings. Public health and medical care implications. *Hypertension* 1989; 14: 570–577
34. Cook NR, Obarzanek E, Cutler JA, et al. Joint effects of sodium and potassium intake on subsequent cardiovascular disease: The Trials of hypertension prevention follow-up study. *Arch. Intern. Med.* 2009; 169: 32–34
35. Cook NR, Appel LJ, Whelton PK. Sodium intake and all-cause mortality over 20 years in the trials of hypertension prevention. *J. Am. Coll. Cardiol.* 2016; 68: 1609–1617.
36. Ministry of Health. *Health Vision 2050. The Main Document*. Undersecretariat for Planning Affairs, Ministry of Health, Sultanate of Oman 2014; pp. 1-212. http://www.nationalplanningcycles.org/sites/default/files/planning_cycle_repository/oman/health_vision_2050_oman.pdf (accessed Jan 14, 2020).
37. World Health Organization. Noncommunicable Diseases Country Health Profiles, 2019. http://www.emro.who.int/entity/statistics/country-health-profiles.html
38. Powles J, Fahimi S, Micha R, et al. Global, regional and national sodium intakes in 1990 and 2010: a systematic analysis of 24h urinary sodium excretion and dietary survey worldwide. *BMJ Open* 2013; 3: e003733.
39. Rodriguez-Fernandez R, Siopa M, Simpson SJ, et al. Current salt reduction policies across gradients of inequality-adjusted human development in the WHO European region: minding the gaps. *Public Health Nutr* 2014; 17(8): 1894-904

For peer review only - http://bmjopen.bmj.com/site/about/guidelines.xhtml

40. Trieu K, Neal B, Hawkes C, et al. Salt reduction initiatives around the world. A systematic review of progress towards the global target. *PLoS ONE* 2015; 10(7): e0130247.
41. D'Elia L, Brajovic M, Klisic A, et al. Sodium and potassium intake, knowledge attitudes and behaviour towards salt consumption, amongst adults in Podgorica, Montenegro. *Nutrients* 2019; 11: 160.
42. D'Elia L, Obreja G, Ciobanu A, et al. Sodium, potassium and iodine intake in a national adult population sample of the Republic of Moldova. *Nutrients* 2019; 11: 2896.
43. Trieu K, Ospanova F, Sharmanov T, et al. A15481 Mean urinary salt excretion in two Kazakhstan regions – one of the highest in the world. *J Hypertens* 2018; 36: e334.
44. Tan M, He FJ, Wang C, MacGregor GA. Twenty-four-hour urinary sodium and potassium excretion in China: a systematic review and meta-analysis. *J Am Heart Assoc* 2019; 8: e012923.
45. Land M-A, Webster J, Christoforou A, et al. Salt intake assessed by 24 h urinary sodium excretion in a random and opportunistic sample in Australia. *BMJ Open* 2014; 4(1): e003720.
46. Pillay A, Trieu K, Santos JA, et al. Assessment of a salt reduction intervention on adult population salt intake in Fiji. *Nutrients* 2017; *9*(12): 1350.
47. Alkhunaizi AM, Al Jishi HA, Al Sadah ZA. Salt intake in Eastern Saudi Arabia. *East Mediterr. Health J.* 2013; 19(11): 915-918.
48. Tayel DI, Amine AK, Elzawi AK. Dietary intake of nutrients related to bone health among Alexandria University female students, Egypt. *Food and Public Health* 2013; 3(6): 329-335.
49. Kelishadi R, Gheisari A, Zare N, Farajian S, Shariatinejad K. Salt intake and the association with blood pressure in young Iranian children: first report from the Middle East and North Africa. *Int. J. Prev. Med.* 2013; 4(4): 475-483.
50. Campbell NRC, He FJ, Tan M, et al. The International Consortium for Quality Research on Dietary Sodium/Salt (TRUE) position statement on the use of 24-hour, spot and short duration (<24-hours) timed urine collections to assess dietary sodium intake. *J Clin Hypertens* 2019; 21: 700-709.
51. McLean RM, Farmer VL, Nettleton A, et al. Twenty-Four– Hour Diet recall and Diet records compared with 24-hour urinary excretion to predict an individual's sodium consumption: A Systematic Review. *J Clin Hypertens* 2018; 20: 1360–1376.
52. McLean RM, Farmer VL, Nettleton A, et al. Assessment of dietary sodium intake using a food frequency questionnaire and 24-hour urinary sodium excretion: a systematic literature review. J *Clin Hypertens* 2017; 19: 1214–1230.
53. Peng Y-G, Li W, Wen X-X, et al. Effects of salt substitutes on blood pressure: a meta-analysis of randomized controlled trials. *Am J Clin Nutr* 2014; 100: 1448-1454

For peer review only - http://bmjopen.bmj.com/site/about/guidelines.xhtml

54. Marklund M, Singh G, Greer R, et al. Estimated population wide benefits and risks in China of lowering sodium through potassium enriched salt substitution: modelling study. *Br Med J* 2020; 369: m824.
55. Cappuccio FP, Buchanan LA, Ji C, et al. Systematic review and meta-analysis of randomised controlled clinical trials on the effects of potassium supplementation on serum potassium and creatinine. *BMJ Open* 2016; 6: e011716
56. Almedawar MM, Nasreddine L, Olabi A, et al. Sodium intake reduction efforts in Lebanon. *Cardiovasc Diagn Ther* 2015; 5(3): 178-185.
57. Nasreddine L, Akl C, Al-Shaar L, Almedawar MM, Isma'eel H. Consumer knowledge, attitudes and salt-reated behaviour in the Middle-East: the case of Lebanon. *Nutrients* 2014; 6: 5079-5102.
58. Ministry of Health (Bahrain). National nutrition survey for adult Bahrainis aged 19 years and above. 2002
59. Derouiche A, Jafri A, El Kardi Y. Assessment of salt, fat content, and intake in largely consumed foods in Morocco. Report of a pilot study. 2014; p. 1-92
60. Bonita R, Magnusson R, Bovet P, et al. Country actions to meet UN commitments on non-communicable diseases: a stepwise approach. *Lancet* 2013; 381: 575-584.
61. Farrand C, He FJ, MacGregor GA. Reducing population salt intake in the Eastern Mediterranean Region – time for urgent action. *Eastern Med. Health J.* 2014; 20(12): 761-764.

Figure 1. Stepwise procedure for the selection of valid participants according to protocol adherence, quality control and completeness of 24h urine collections.

For peer review only - http://bmjopen.bmj.com/site/about/guidelines.xhtml

28 Jul 2020 – v13- R4

Table 1. Geographical sampling from the Sultanate of Oman.

Governorate	Valid 24h urine collections	%
Muscat	67	11.8
Dhofar	79	13.9
Al-Dakhlia	45	7.9
North Sharqiah	36	6.3
South Sharqiah	45	7.9
North Batina	81	14.2
South Batina	53	9.3
Al-Dhahirah	46	8.1
Al Buraymi	84	14.8
Musandam	9	1.6
Al-Wasta	24	4.2
**Total**	**569**	**100.0**

28 Jul 2020 – v13- R4

Table 2. Characteristics of the participants

**Variable**	**All (n=569)**	**Men (n=193)**	**Women (n=376)**
Age (years)	39.4 (13.1)	38.7 (14.3)	39.8 (12.5)
Height (cm) ʃ	159.4 (11.2)	167.9 (9.7)	154.9 (9.2)*
Weight (kg) ʃ	74.9 (21.5)	81.4 (22.5)	71.4 (20.1)*
BMI (kg/m^2) ʃ	29.3 (7.2)	28.9 (7.6)	29.5 (7.0)
Waist circumference (cm) ʃ	93.8 (15.7)	95.0 (15.7)	93.2 (15.7)
Hip circumference (cm) ʃ	104.5 (15.0)	102.6 (13.7)	105.5 (15.6)†
Systolic blood pressure (mm Hg) ‡	125.9 (18.2)	134.0 (17.0)	121.7 (17.3)*
Diastolic blood pressure (mm Hg) ‡	80.9 (10.7)	83.4 (11.5)	79.7 (10.1)*
Pulse rate (b/min) ‡	79.8 (10.5)	78.5 (11.8)	80.4 (9.8)†
Hypertension N (%) ‡	175 (30.8)	77 (39.9)	98 (26.1)*
On anti-hypertensives N (%) ‡	50 (28.6)	15 (19.5)	35 (35.7)†

Results are mean (SD) or N(%),
‡ 3 missing values (1m, 2w) (0.5%); ʃ 18 missing values (1m, 17w) (4%);
* $p<0.01$, † $p<0.05$ when compared to men
Hypertension: SBP/DBP ≥140/90 mmHg or on current therapy for high blood pressure

Table 3. Daily urinary excretions of volume, sodium, potassium and creatinine, estimates of salt and potassium intake, and proportion of participants meeting WHO recommended targets for salt and potassium consumption.

**Variable**	**All (n=569)**	**Men (n=193)**	**Women (n=376)**
Volume (mL/24h)	1354 (725)<br>1129 (855-1618)	1392 (712)<br>1150 (900-1721)	1335 (731)*<br>1122 (827-1593)
Sodium (mmol/24h)	144.3 (78.8)<br>129.6 (85.7-187.4)	154.2 (87.4)<br>135.4 (87.1-204.8)	139.2 (73.6)*<br>126.8 (83.9-179.4)
Potassium (mmol/24h)	52.6 (32.6)<br>46.4 (31.4-64.9)	56.4 (32.4)<br>50.9 (33.8-73.2)	50.6 (32.5)*<br>44.7 (30.2-61.6)
Sodium-to-Potassium Ratio	3.3 (3.4)<br>2.8 (2.0-3.8)	3.5 (4.2)<br>2.7 (1.9-3.9)	3.2 (2.8)<br>2.8 (2.1-3.8)
Creatinine (g/24h)	1.33 (0.71)<br>1.18 (0.86-1.63)	1.72 (0.87)<br>1.61 (1.16-2.12)	1.13 (0.52)†<br>1.02 (0.81-1.36)
Salt intake (g/day)	9.0 (4.9)<br>8.1 (5.3-11.7)	9.6 (5.5)<br>8.5 (5.4-12.8)	8.7 (4.6)*<br>7.9 (5.2-11.2)
Potassium intake (g/day)	2.36(1.46)<br>2.08 (1.41-2.91)	2.53 (1.45)<br>2.28 (1.52-3.28)	2.27 (1.46)*<br>2.00 (1.35-2.76)
Salt <5g/day N (%)	124 (21.8)	40 (20.7)	84 (22.3)
Potassium >90 mmol/day N (%)	52 (9.1)	24 (12.4)	28 (7.4)#

Results are mean (SD) and median (25th-75th percentile) or N (%); *$p<0.05$; #$p=0.008$, †$p<0.001$ when compared to men

For peer review only - http://bmjopen.bmj.com/site/about/guidelines.xhtml

Table 4. Knowledge, attitudes and behaviours towards salt consumption.

**Question**	**All (n=569)**	**Men (n=193)**	**Women (n=376)**
How often do you add salt or salty sauces to your food?			
Often/Always	28.1 %	22.8 %	30.8 % *
Sometimes	21.3 %	17.1 %	23.4 %
Rarely/Never	50.6 %	60.1 %	45.8 %
How often is salt, salty seasoning or salty sauces added in cooking or preparing food at home? ‡			
Often/Always	47.0 %	44.8 %	48.1 % #
Sometimes	16.5 %	12.5 %	18.6 %
Rarely/Never	36.5 %	42.7 %	33.3 %
How often do you eat processed food? ‡			
Often/Always	22.3 %	22.8 %	22.1 %
Sometimes	38.3 %	35.8 %	39.5 %
Rarely/Never	39.4 %	41.4 %	38.4 %
How much salt or salty sauces do you think you consume? ‡			
Too much/Far too much	6.9 %	7.8 %	6.4 %
Just the right amount	66.8 %	61.3 %	69.7 %
Too little/Far too little	26.3 %	30.9 %	23.9 %
Do you think that too much salt or salty sauces could cause a serious health problem? ‡			
Yes	89.1 %	90.1 %	88.6 %

Results are percentages *p=0.005; #p=0.04 when compared to men. ‡ reduced numbers due to missing values

28 Jul 2020 – v13- R4

Table 5. Frequency of other dietary patterns

Question	All (n=569)	Men (n=193)	Women (n=376)
In a typical week, on how many days do you eat fruit? ‡			
< 5	32.0 %	40.4 %	27.7 %**
≥ 5	68.0 %	59.6 %	72.3 %
How many servings of fruit do you eat on one of those days? ‡			
<3	45.5 %	41.8 %	47.2 %
≥3	54.5 %	58.2 %	52.8 %
In a typical week, on how many days do you eat vegetables? ‡			
< 5	24.1 %	24.9 %	23.7 %
≥ 5	75.9 %	75.1 %	76.3 %
How many servings of vegetables do you eat on one of those days? ‡			
<3	59.9 %	65.9 %	56.9 %[#]
≥3	40.1 %	34.1 %	43.1 %
What type of oil or fat is most often used for meal preparation in your household? ‡			
Vegetable oil	90.8 %	91.1 %	90.7 %
Other (lard, suet, butter, ghee)	9.0 %	8.9 %	9.0 %
None used	0.2 %	0	0.3 %
On average, how many meals per week do you eat that were not prepared at home? ‡			
0	45.8 %	32.1 %	52.8 %*
≥1	54.2 %	67.9 %	47.2 %
Results are percentages *p<0.0001; **p=0.002; [#]p=0.04 vs men. ‡ reduced numbers due to missing values			

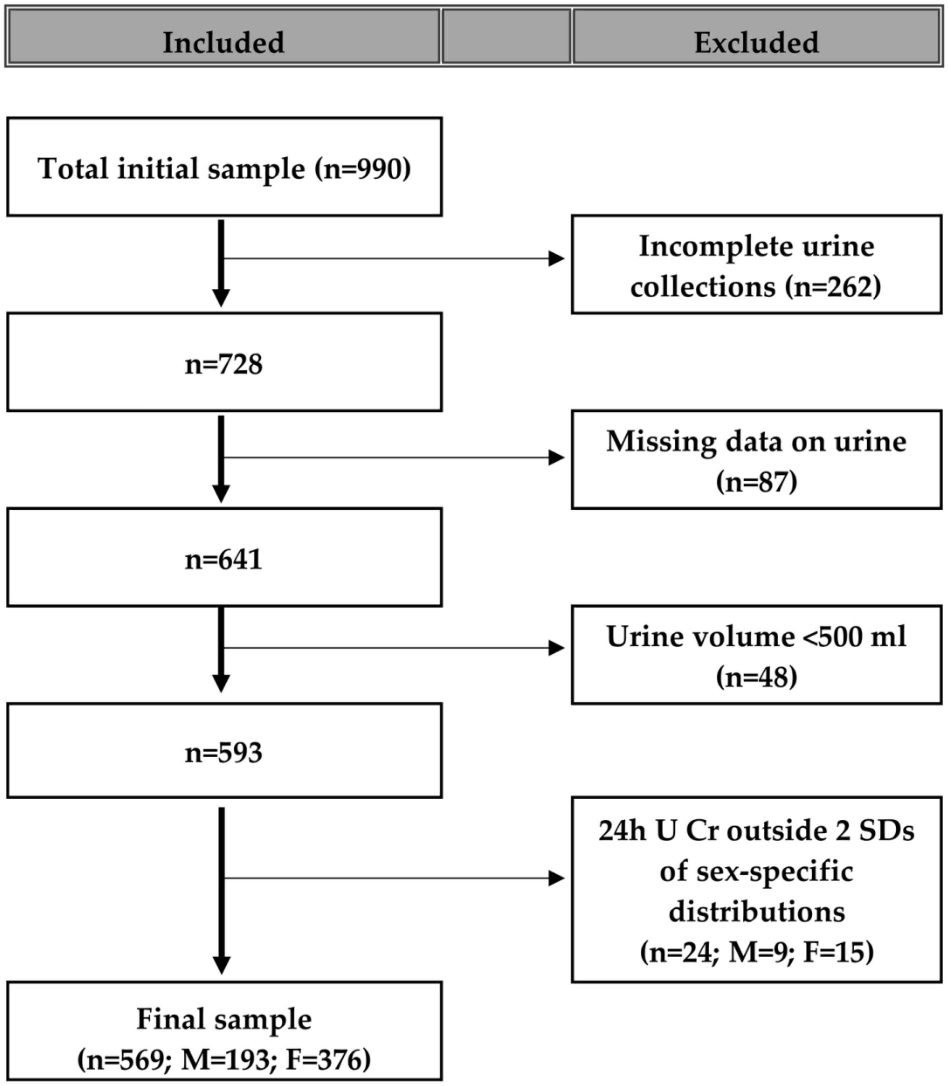

Figure 1. Stepwise procedure for the selection of valid participants according to protocol adherence, quality control and completeness of 24h urine collections.

129x148mm (300 x 300 DPI)

SUPPLEMENTARY MATERIAL

TEXT S1. MATERIAL AND METHODS

1.1 Study Design

A national cross-sectional population-based survey was conducted using a representative sample of the Sultanate of Oman based on the WHO Stepwise approach to Surveillance (STEPS) of NCD risk factors (1), comprising the following aspects:

- **Step 1:** This consisted of face-to-face interviews using advanced standardized nation-specific version of the STEPS questionnaire and locally-adapted show cards to facilitate the understanding and operationalization of some questions. Socio-demographic characteristics, key behavioural risk factors, lifestyles, eye and ear health, history of chronic diseases and health care coverage were also elicited to better define exposure and health care seeking and control.
- **Step 2:** This involved physical measurements (e.g. weight, height, waist, hip circumference), and determination of blood pressure, heart rate, and vision function to investigate biological risk factors such as hypertension, overweight, obesity, and vision issues.
- **Step 3:** This aimed at determinations of biochemical markers levels (e.g. fasting capillary blood for glucose and lipid profile, and non-fasting urinary samples for sodium and creatinine) to identify hypercholesterolemia, hyperglycaemia, and sodium intake.

1.2 Sample Design

2.1 **Target Population:** The population of interest was all non-institutionalized persons, which included all men and women 18 years of age or older (Omani & non-Omani) who reside in the country.

2.2 **Inclusion population:** The target population includes all persons who consider the country to be their usual place of residence. This definition comprises those individuals residing in the country even though they may not be considered a citizen of the country.

2.3 **Exclusion population:** Those household members who were younger than 18 years of age; persons who have cognitive impairment that hampered understanding the questions to provide clear feedback; visitors (tourists) to the country; and, institutionalized people or those who indicated their usual place of residence was a military base, labour camps or group quarters, were excluded from the survey.

1.3 Sample Frame

For the 2010 National census, the whole of the Sultanate of Oman was divided into 15,077 census blocks. A census block was defined as a collection of units (residential units), which

For peer review only - http://bmjopen.bmj.com/site/about/guidelines.xhtml

includes 60 units or less, and it could involve one or more enumeration areas. The census block is the smallest unit of field work for census data collectors. A total of 399,274 households[1] were identified in these blocks (260,120 Omani and 139,154 non-Omani). Households for Omanis and non-Omanis could not be differentiated unless physically visited as they lived closely together and there were no specific places (blocks) for non-Omani. The number of blocks and households in each governorate was provided in the table below according to the National Census 2010 and the map below shows the distribution of blocks over the Sultanate of Oman by governorates (National Centre for Statistics & Information (NCSI), Sultanate of Oman). The same census blocks were updated and used in this NCD Risk Factor Survey as shown in Table 1.

Table 1: Number of blocks and households by governorate based on 2010 National Census comprising the sampling frame for Oman NCD risk factors survey, 2017

Governorate	Number of blocks	Number of Omani households
**Muscat**	3514	62299
**Dhofar**	1768	17926
**Ad Dakhiliyah**	1712	34611
**North Sharqiyah**	1173	20044
**South Sharqiyah**	1097	22455
**North Batinah**	2248	47193
**South Batinah**	1335	29355
**Al-Dhahirah**	884	14386
**Al-Buraimi**	531	5939
**Musandam**	513	3216
**Al-Wusta**	302	2696
**Total**	**15077**	**260120**

1.3.1 Mapping and listing update

Two persons were appointed and trained to perform the mapping and listing operation for each governorate by using 2010 census maps from the National Centre for Statistics and Information. Personnel visited selected blocks to update maps that depicted all the households within the selected block and update the list of households within those blocks, including

[1] A "Household (HH)" was defined as "either a one-person household, defined as an arrangement in which one person makes provision for his or her own food or other essentials for living without combining with any other person to form part of multi-person household or a multi-person household, defined as a group of two or more persons both related and unrelated living together who make common provision for food or other essentials for living".

For peer review only - http://bmjopen.bmj.com/site/about/guidelines.xhtml

additional information (e.g. house number - Name of household head – nationality – language – description if needed).

1.4 Sampling Design

A multi-stage stratified cluster sampling was designed to select 9053 eligible subjects. Stratification was made on two factors: governorate and nationality (Omani and Non-Omani). The sample was drawn from the 2010 census block area (clusters). An ***equal size sample (cluster)*** was systematically randomly selected from each governorate. Selected clusters (blocks) had to be updated before the households were selected for survey (see previous section).

For peer review only

1.4.1 Rationale for preferring equal sample selection rather than proportional to population size

1. To get the desired precision and estimates of overall results and figures at the governorate level.
2. Proportional allocation would have resulted in a very small sample in a small governorate e.g. Al-Wusta with low precision in the estimate.
3. Equal sampling allowed high precision in individual stratum.
4. The experience from previous surveys in Oman. The lowest response rate was in Muscat governorate and one third of population live in Muscat so if proportional selection was used this would have directly affected the overall response rate.

In the first stage, all governorates in the Sultanate of Oman (11 governorates) were selected.

In the second stage, we stratify blocks (PSU) based on geographical location, 550 clusters, from all over the Sultanate of Oman (50 from each governorate) using systematic random sampling (Secondary Sampling Units (SSU)).

In the third stage: [Tertiary Sampling Unit (TSU)], the following was considered:

i) The households in each cluster were listed within each governorate i.e. households from the first cluster, then households from the second cluster till households from the 50th cluster.

ii) The households in all clusters were aggregated into two lists by nationality (Omani and Non-Omani households) in each governorate

iii) 823 households were systematically and randomly selected from list of all households according to above in each governorate, from these two lists (Omani and Non-Omani household lists) according to the ratio of Omani and Non-Omani households in each governorate.

In the fourth stage: Ultimate sampling unit USU), One eligible individual from each household aged 18 years old or older was selected randomly by a program on the Android tablet.

1.4.2 Sample Size

A WHO STEPS standard formula was used in the calculation of the sample size based on the guidelines/recommendations of the STEPS survey.

$n= Z^2 * P (1-P)/d^2$

Where;

n= the required sample size

Z= the probability value associated with the confidence level

P= the prevalence rate of NCDs risk factors in the country

d= the desired margin of error (precision).

In turn:

Z= 1.96 (95% confidence interval as recommended)

P= 0.5 (the conservative value of prevalence rate)

e= 0.05 (as recommended in the guidelines)

Using these values, the initial calculation was: n= 384 households

Also taken into account for sample size calculations were: a value of design effect, as recommended in STEPS surveys, to be 1.5; and, an anticipated response rate of 70% was estimated. By adjusting the sample by these factors, the sample size per cluster (governorate) results in:

$$n = \frac{384 \ x \ 1.5}{0.7} = 823 \text{ households}$$

To get the desired precision and overall figures adequate for age-sex groups and for overall estimate on governorates level, the sample size was:

n= 823 x 11 = 9053 households, with one individual selected per household

The 9053 households were distributed equally by governorate (823), proportional to nationality, according to the ratio of Omani and non-Omani households in each governorate as in Table 2.

Table 2: Oman STEPS survey sample size distributed, by governorate and nationality, 2017

Governorate	Selected no. of blocks in each governorate	Omani	Non-Omani	Total Households

**Muscat**	50	318	505	**823**
**Dhofar**	50	495	328	**823**
**Ad Dakhiliyah**	50	763	60	**823**
**North Sharqiyah**	50	520	303	**823**
**South Sharqiyah**	50	667	156	**823**
**North Batinah**	50	677	146	**823**
**South Batinah**	50	699	124	**823**
**Al-Dhahirah**	50	586	237	**823**
**Al-Buraimi**	50	722	101	**823**
**Musandam**	50	713	110	**823**
**Al-Wusta**	50	663	160	**823**
**Total**	**550**	**6823**	**2230**	**9053**

1.5 Data collection instruments and procedures used in the survey

1.5.1 Selection of participants

Upon selection of a household, all potential individuals for interview were identified and their age and gender recorded in a household list, subsequently used to determine selection probabilities and response rate. To randomly select an eligible individual from a household, the Kish method was used using an electronic tablet and software. Once individuals were selected, they were informed about the survey aims and asked to provide their consent to participate in the interview and subsequent measurement procedures.

1.5.2 Questionnaire (STEP 1)

An advanced standardized[2] country-specific version of the questionnaire, based on the Core and Expanded version of the WHO STEPS Instrument for Chronic Disease Risk Factor Surveillance[3] and country-specific requirements, contained 11 Core, 1 Optional and 4 country-specific modules, to determine socio-demographic characteristics of participants, key behavioural risk factors (tobacco use, harmful alcohol consumption, diet with low fruits and vegetable intake, high salt intake, physical inactivity), given lifestyle advises, eye and ear health profile, history of chronic diseases, and health care coverage for diabetes, hypertension and dyslipidaemia, as well as cervical and breast screening.

In order to enhance the comparability with other countries in Arab region, the questionnaires from the Kuwait (2014), the Qatar (2012), the Bahrain (2007) and the Saudi Arabia (2005) STEPS surveys were taken into consideration. In addition, the questionnaire was translated from the original English version into Arabic as well as back translated, adapted to the local environment and needs, and piloted on 10 eligible respondents in terms of wording and

2 validated
3 Reference: The WHO STEPS Instrument for Chronic Disease Risk Factor Surveillance (1)

For peer review only - http://bmjopen.bmj.com/site/about/guidelines.xhtml

understanding. The data collection was conducted in two languages, namely: Arabic and English.

Table 3: Questionnaire content by STEPS survey and county-specific modules and questions in the Oman survey, by type, 2017

	STEP and county-specific modules content	Questions		
**I**	**Core modules (11)**	**Core**	**Expanded**	**Country-specific**
1	Demographic information	4	5	3
2	Tobacco use	11	7	
3	Alcohol consumption	12		
4	Diet, including dietary salt	8	3	2
5	Physical activity	15	1	
6	History or raised blood pressure	5		1
7	History of diabetes	6		1
8	History of raised total cholesterol	5		1
9	History of cardiovascular disease	3		
10	Lifestyle advise	1		
11	Cancers (cervical cancer screening)	1		3
	**Subtotal 1**	71	16	11
**II**	**Optional Module (1)**			
1	Tobacco policy	7		8
	**Subtotal 2**	7		8
**III**	**Country-specific Modules (4)**			
1	Family history of chronic disease			8
2	Asthma			11
3	Eye health			3
4	Ear health			4
	**Subtotal 3**			26
**Grand Total: 139**		**78**	**16**	**45**

1.5.2.1 Demographic information

All eligible household members aged 18 and above were listed with one eligible member selected randomly to answer the demographic information questionnaire. Demographic information was assessed in terms of age, sex, marital status, educational status, employment status, and family income.

1.5.2.2 Tobacco use

Tobacco use was assessed in terms of current smoking (past 30 days), whether daily or non-daily, and former smoking or never smoking status, age of initiation and duration of smoking, type and quantity of tobacco use daily or weekly, smokeless tobacco use type and frequency, and exposure to second-hand smoke at home or workplace. Smoking cessation attempts, having

For peer review only - http://bmjopen.bmj.com/site/about/guidelines.xhtml

received health professionals' advice, age and time since stopping to smoke, recognizing tobacco advertisement, promotion or sponsorship, all while applying 33 questions[4] were also asked. To facilitate recognition of types of tobacco use, data collectors used show cards, depicting types of commonly used tobacco products. Likewise, the above metrics were determined for users of smokeless tobacco products.

1.5.2.3 Alcohol consumption

Alcohol consumption was assessed using the concept of a standard drink. A standard drink is any drink containing about 10 g of pure alcohol. Accordingly, determinations have been made from different types of alcoholic beverages consumed, as follows: e.g. 30 ml of spirits, 120 ml of wine or 285 ml of beer. Again, data collectors used show cards depicting types of containers commonly used to consume alcoholic beverages as standard drinks, to determine consumption over 30 and 7 days prior to interview. Also, in an attempt to quantify and estimate total alcohol consumption, interviewers considered not only the most frequent but all types of alcohol consumed (e.g. wine, beer and spirits) and the amount of drinks on such occasions. Also included in the questionnaire were aspects about stopping alcohol consumption for health reasons or impacts.

Respondents who reported consuming alcohol within the past 30 days were classified as current drinkers, while those who identified absence of alcoholic beverages within previous 12 months as abstainers or ex-drinkers. Three risk categories were used to classify respondents who consumed alcohol according to the average amount of alcohol consumed per occasion. Furthermore, heavy ("binge") drinking patterns were determined according to largest number of drinks per drinking occasion and the percentage of those having consumed six or more standard drinks on one occasion during the past 7 days.

1.5.2.4 Diet

To assess the dietary patterns of the surveyed population, the respondents were asked about frequency of fruit and vegetable consumption, mean number of portions of these foods consumed daily and weekly, type of oils and fat used for meal preparation, number of meals eaten outside the household per week and the amount of salt added, and/or salty sauces used or processed food or consumed daily, using 13 questions.

Sufficient consumption of fruit and vegetables was assessed in terms of the number of servings and also compared to WHO recommended number of $\geq$ 5 servings/day and $\geq$ 5 day/week, with

[4] Based on STEPS and GATS questionnaires (2)

For peer review only - http://bmjopen.bmj.com/site/about/guidelines.xhtml

For peer review only

a serving being equal to 80 g. Show cards were used to facilitate the collection data on fruit and vegetable consumption on a typical day. Oil and fat intake show cards were also shown to assess about the type of oil or fat most frequently used for preparing food or cooking.

In turn, salt consumption was assessed by asking about frequency of addition of salt or a salty sauce to food during preparation, or before or while eating; and/or frequency of consumption of processed food high in salt. Participants were also asked about their perception of the quantity of salt they consumed and its link with health problems, as well as about the importance of reducing salt intake, and the measures undertaken to control it. WHO recommends a reduction to <2 g/day sodium (5 g/day salt) in adults (3).[5]

Population mean number of daily portions and of days per week consuming fruits and/or vegetables were calculated. Also, the percentage distribution of respondents according to their servings consumed per day and those meeting the WHO recommendation of fruits and vegetables intake/day were determined.

Regarding salt intake, the proportions of people reporting how often they added salt to foods before eating or when preparing foods, as well as those who think they eat too much salt were determined. Percentage of participants were further determined according to their belief on the importance of salt in diet and whether they thought their consuming to much salt can cause serious problems.

1.5.2.5 Physical activity

Physical activity was assessed based on frequency, duration and intensity of physical activity at three segments: work (paid/ unpaid in and outside home), during transportation and on leisure time, for at least 10 minutes or more continuously per day, using a set of 16 questions[6]. Show cards were used to depict different types and places of physical activity.

According to WHO global recommendations on physical activity for (good) health, throughout a normal week adult should do at least the following amount of exercise (including activity for work, as well as during transport and leisure time): 150 minutes of moderate-intensity physical activity; or 75 minutes of vigorous-intensity physical activity; or an equivalent combination of moderate- and vigorous-intensity physical activity. Mean and median minutes of physical activity per day according to place were computed; as a complement, time spent on sedentary activities on average per day was also calculated. The proportion of respondents not meeting

[5] http://www.who.int/nutrition/publications/guidelines/sodium_intake_printversion.pdf
[6] Based on WHO Global Physical Activity Questionnaire (version 2) (4)

For peer review only - http://bmjopen.bmj.com/site/about/guidelines.xhtml

the WHO recommendations was also calculated. Likewise, the proportion of participants according to levels of physical activity as recommended by WHO were determined.

1.5.3 Physical measurements (STEP 2)

1.5.3.1 Blood pressure

Resting blood pressure levels, both systolic (SBP) and diastolic (DBP), were measured using Omron M3 digital blood pressure device as recommended by WHO. The measurements were repeated three times and the three readings were recorded. In order to obtain the measurements under relaxed conditions, persons were asked to void their bladder if needed, resting for 10-15 minutes after the interview, and not having drunk coffee before or during the measurement. In preparation for measurements, participants were asked to sit straight without crossing their legs. Blood pressure was measured placing a universal cuff on the left arm, which was placed with the palm face upward on a table surface at the level of the heart. While taking the readings participants were asked to remain silent. Repeat measurements were taken at 3-minute interval.

Participants were classified according to their blood pressure readings in the following categories: *normal* if their SBP and DBP readings were <140 mm Hg and <90 mmHg, respectively, and *high* if their SBP was ≥ 140 mm Hg and/or the DBP was ≥ 90 mm Hg, or if their readings were normal but they were under treatment for raised blood pressure in the past two weeks. In addition, *high risk* levels of SBP ≥ 160 mm Hg and/or DBP ≥ 100 mm Hg were also determined among participants to assess a higher probability or risk of hypertensive disorder.

Survey participants were also asked whether they were under medication for high blood pressure during the previous two weeks, as prescribed by a physician or other health professional. Respondents with treated and/or controlled raised blood pressure among those with raised blood pressure (SBP ≥140 and/or DBP ≥ 90 mmHg) or currently taking medication for raised blood pressure were further categorized to determine treatment success, treatment failure or being undetected and untreated, as follows:

- Under medication and controlled (treatment success) = those taking medication and having SBP <140 mmHg and DBP <90 mmHg;
- Under medication and uncontrolled (treatment failure) = those taking medication and having SBP ≥140 mmHg and/or DBP ≥90 mmHg;
- Undetected and uncontrolled (health system failure) = those not taking medication and having SBP ≥140 mmHg and/or DBP ≥90 mmHg**.**

For peer review only - http://bmjopen.bmj.com/site/about/guidelines.xhtml

1.5.3.2 Body mass index (BMI)

The height and weight of participants was taken to estimate their body mass index (BMI) as the ratio of weight/height^2 (at the nearest decimal kilogram and decimal centimetre, respectively).

Measurements were carried out while standing with the heels together, feet apart, arms at sides and chin parallel to the floor on a flat, horizontal and firm surface (like tile, cement or wooden floor). Standardized and calibrated SECA® 813 digital floor scales and 213 portable stadiometers were used for weight and height measurements, respectively. To measure height and weight more precisely, participants were asked to follow standard procedures, including removal of their shoes and any bulky or heavy clothing to avoid overestimations.

Once BMI ratios determined, sample population was categorized according to the following WHO recommendations: underweight if BMI < 18.5, normal weight if BMI was between 18.5 - 24.9, overweight if BMI was between 25.0 - 29.9, and obesity if BMI was ≥ 30.

Average population BMI levels and proportion distribution among the sample population groups were determined.

1.5.3.3 Waist and hip circumferences

Waist circumference and hip circumference and their ratio were also assessed as other measures of obesity, in particular of central obesity.

Waist and hip circumference measurements were made while a participant remained standing, with feet together and hands on each side of the body, with a non-stretch Seca 201 measuring tape with millimetre precision. Waist circumference was measured by placing a tape measure around the bare abdomen at the midpoint between the lower margin of the last palpable rib and the top of iliac crest (hip bone). Hip circumference was measured by placing a tape measure around the maximum circumference over the buttocks.

The WHO cut-off points of waist circumference that determine waist obesity and categorized risk of metabolic complications and CVD are different for men and women. The waist–hip ratio (WHR) was computed among all respondents, excluding pregnant women.

1.6 Data collection

1.6.1 Staff recruitment

Interviewers and field supervisors in each governorate were recruited from among staff working in the Ministry of Health.

For peer review only - http://bmjopen.bmj.com/site/about/guidelines.xhtml

Overall, 66 data collectors and 11 field supervisors were recruited to participate in the training of data collection along with 1 regional coordinator, 1 IT technician and 1 laboratory technician in each governorate. Data collectors were mainly nurses and health educators nominated for data collection and for measurement of height, weight, waist and hip circumference, blood pressure, blood glucose, lipid profile, and vision testing. Urine samples were measured by the laboratory technicians in the health centre laboratories.

1.6.2 Data collection procedures

Field operations were carried out in the governorates during a four-month period in 2017, with the survey period chosen appropriately to avoid Ramadan/Eid periods. A media and advocacy action plan was implemented to raise awareness of the population about the survey, including disseminating information through leaflets, posters, press releases, radio and TV broadcasting, community and local Ministry of Health (MoH) staff participation. In addition to this focused publicity campaign, official identity cards to the field staff issued by MoH were of great help to secure sufficient recognition, cooperation and good responses for the interview in most cases. The respective authorities were also requested to provide the necessary assistance and co-operation to the field staff.

The interviews were conducted in all the governorates. The supervisors approached the selected households in each cluster, explained the aim and objectives of the survey, and sought their consent to participate in the survey. After recording the eligible members within a selected household, one participant was randomly selected from eligible members by the Android tablet. Each interview took place in a secure setting with adequate privacy at the household level. Each participant was interviewed at his/her household. As biomedical tests require 12 hours of fasting, appointments were given based on agreement between the interviewers and the respondents. Interviewers also explained the protocol for 24-hour urine collection to the respondent, obtained informed consent and provided the record sheet on which participants note the start and finish times of their 24-hour urine collection and any missed urine collections in the container.

1.6.3 Training for supervisors and data collectors

A one-week training program was conducted by the central research team from the Centre of Studies & Research in collaboration with WHO experts. This training program, which was held in Muscat in December 2016, included the survey objectives and field work staff duties, how to fill in the questionnaire in a polite, motivating and persuasive manner, how to understand the content of the questions if needed to clarify to respondents, how to enter the data and navigate

For peer review only - http://bmjopen.bmj.com/site/about/guidelines.xhtml

the Android tablet, how to ask for written consent and organize data collection and protection of confidentiality of the informant (consent, ID barcode labels, patterns of verbal and nonverbal behaviour), how to perform physical measurements and take biomarkers' samples using equipment applying standardized methodology, and role playing and mock interviews.

The field supervisors were given special instructions afterwards on coordinating process. Trainees who failed to show interest in the survey and those who did not attend the training program on a regular basis were not selected for the fieldwork and were replaced by other staff from the same Governorate after training.

1.6.4 Pilot Study

The pilot testing of the survey implementation process was implemented for 2 days (using Android tablets) selecting about 100 households which included males and females as well as Omani and non-Omani households. The pilot was also used to train the key survey personnel, test all survey materials prior to full implementation (skip errors, translation errors, awkward wording, and inadequate response categories), check quality of data collected. Lessons learned, logistic issues, and challenges identified were considered which maximized opportunities for improving the quality in the full survey implementation.

1.7 Data Management

1.7.1 Data collection

Each participant was allocated an identifier code (PID). The PID code consisted of seven digits the first two digits were the device number; the next three digits were the house listing number in the cluster and the last two digits were the person number in the household.

Data from STEP 1 and STEP 2 were submitted electronically online to the server from a household with identification of individual PID, household geographical location, including cluster with individual PID, day and time of completion. This was done either daily or at least once a week. After being analysed, blood and urine samples' results (STEP 3) were uploaded to the server and merged into the unified dataset, following conversion into SPSS and Microsoft Excel format in a single file.

1.7.2 Data validation

The central team (at the Centre of Studies & Research) downloaded data daily from server for data cleaning and management over a period of 6 months. Data management included continuously monitoring data collection, uploading and consolidation processes in the field,

For peer review only - http://bmjopen.bmj.com/site/about/guidelines.xhtml

validating quality of the data, creating weights, removing inconsistencies, namely “jump” errors/outliers, absence of data, excess data and invalid data. Moreover, to increase reliability of the collected data, verification of data in field was organized among 500 randomly selected households from all governorates. Accuracy of recording categorical and continued variables was checked using range and logic functions. The team also provided advice on software support and reported any problems or interview errors to the data collection field supervisors.

1.7.3 Data analysis

Weightage and Adjustment for sampling variation

Survey data analyses have to take into account whether the results are representative of the sample alone (unweighted analysis) or of the entire target population (weighted analysis). Since the primary objective was to be able to determine the estimates for the whole country, a weighted analysis was considered necessary. Weights adjusting for this complex survey design were required to decrease the risk of biases resulting from diverse factors. The sample weight is comprised of the inverse probability of selection. The household weights took into account the selection probability of the clusters within each stratum and the size (the number of households) of the cluster. The sample weight was also adjusted for non-response at the household level. The individual weight assumed that adults in the same cluster were selected by simple random sampling but the calculation scheme did not take into account the household size. This approach could have biased any key indicators, which was strongly associated with the household size. The individual weight was also adjusted for non-response.

Means, medians, proportions, standard errors, and 95% confidence intervals (95% CI) values were calculated to estimate central and dispersion measures and used to assess prevalence differences of NCD risk factors. Statistical procedures for data calculation and analyses were performed through two programs: EpiInfo in collaboration with WHO, and IBM SPSS (Version 20). All the figures and indicators in the tables were calculated using SPSS complex samples analysis. The figures presented as footnote (with an asterism) under each table were calculated after using population proportion weight. To allow for international comparisons of Oman survey results, age- and sex-adjusted overall values were calculated for all indicators using the direct method and the WHO standard population. Values are presented as footnotes on the tables to limit confusion with the national unadjusted data. It should also be noted that the estimates shown for governorates in the tables should be treated with caution as they represent the respondents in the respective governorate, and not the governorate itself.

For peer review only - http://bmjopen.bmj.com/site/about/guidelines.xhtml

For peer review only

1.8 Ethical considerations

Two informed consent forms, one for filling in the questionnaire and performing physical measurements (e.g. STEP 1 and STEP 2) and another for taking blood and urine samples for biomarkers (STEP 3), were requested to be signed by each participant. To enhance participation, an information letter was sent to all selected households in advance of data collection, identifying purpose, benefits and the voluntary participation in the survey.

To guarantee the high level of confidentiality and data security, every eligible subject was granted a unique identification number which was used for any reference from the register, with the exception of providing a personal feedback to a particular eligible subject for medical reasons.

Prior to its implementation, the survey was approved by the Research and Ethical Review & Approval Committee (RERAC) of the Ministry of Health.

For peer review only

Table S1. Comparison of the general characteristics of the study participants with those of the national sample of the 2017 WHO STEPS Survey carried out in Oman.

	Salt and Potassium Survey mean (SD)			2017 WHO STEPS National Survey † mean (95% CI)		
	**All (n=569)**	**Men (n=193)**	**Women (n=376)**	**All (n=6,582)**	**Men (n=3,365)**	**Women (n=3,217)**
Age (yrs)	39.4 (13.1)	38.7 (14.3)	39.8 (12.5)	38.2 (37.4-39.0)	38.0 (37.1-38.8)	38.6 (37.3-39.8)
Height (cm)	159.4 (11.2)	167.9 (9.7)	154.9 (9.2)	-	167.4 (166.6-168.2)	156.1 (155.5-156.6)
Weight (kg)	74.9 (21.5)	81.4 (22.5)	71.4 (20.1)	-	74.9 (73.6-76.2)	68.1 (66.6-69.7)
BMI (kg/m²)	29.3 (7.2)	28.9 (7.6)	29.5 (7.0)	27.3 (26.9-27.6)	26.7 (26.3-27.2)	27.9 (27.3-28.5)
Waist circ. (cm)	93.8 (15.7)	95.0 (15.7)	93.2 (15.7)	-	90.0 (89.0-91.0)	87.6 (86.1-89.1)
Hip circ. (cm)	104.5 (15.0)	102.6 (13.7)	105.5 (15.6)	-	101.2 (98.8-103.5)	102.5 (99.2-105.7)
Systolic BP (mmHg)	125.9 (18.2)	134.0 (17.0)	121.7 (17.3)	124.9 (124.0-125.7)	130.3 (129.2-131.4)	119.1 (117.7-120.4)
Diastolic BP (mmHg)	80.9 (10.7)	83.4 (11.5)	79.7 (10.1)	79.5 (78.9-80.1)	81.5 (80.6-82.5)	77.4 (76.7-78.1)
Pulse rate (b/min)	79.8 (10.5)	78.5 (11.8)	80.4 (9.8)	79.4 (78.7-80.2)	77.7 (76.4-78.9)	81.3 (80.6-82.0)
Hypertension (%)	30.8	39.9	26.1	27.5	33.1	21.5

†https://www.who.int/ncds/surveillance/steps/Oman_STEPS_2017_Data_Book.pdf?ua=1

For peer review only - http://bmjopen.bmj.com/site/about/guidelines.xhtml

Table S2. Characteristics of excluded participants and comparison with those included in the final analysis.

**Variable**	**Included (n=569)**	**Excluded (n=159)**	**P value***
Age (years)	39.4 (13.1)	37.5 (13.9)	0.037
Height (cm)	159.4 (11.2)	158.7 (8.6)	>0.05
Weight (kg)	74.9 (21.5)	73.4 (18.3)	>0.05
BMI (kg/m²)	29.3 (7.2)	29.1 (7.0)	>0.05
Waist circumference (cm)	93.8 (15.7)	92.3 (17.9)	>0.05
Hip circumference (cm)	104.5 (15.0)	102.6 (16.4)	>0.05
Systolic blood pressure (mm Hg)	125.9 (18.2)	124.2 (18.8)	>0.05
Diastolic blood pressure (mm Hg)	80.9 (10.7)	79.1 (11.6)	0.025
Pulse rate (b/min)	79.8 (10.5)	80.9 (10.5)	>0.05
Hypertension (%)	27.4	23.9	

Results are mean (SD), *by Mann-Whitney U-test
Hypertension: SBP/DBP >=140/90 mmHg

For peer review only

STROBE Statement—Checklist of items that should be included in reports of ***cross-sectional studies***

	Item No	Recommendation	Page No
**Title and abstract**	1	(*a*) Indicate the study's design with a commonly used term in the title or the abstract	1,2
		(*b*) Provide in the abstract an informative and balanced summary of what was done and what was found	2
**Introduction**			
Background/rationale	2	Explain the scientific background and rationale for the investigation being reported	4
Objectives	3	State specific objectives, including any prespecified hypotheses	5
**Methods**			
Study design	4	Present key elements of study design early in the paper	5-6
Setting	5	Describe the setting, locations, and relevant dates, including periods of recruitment, exposure, follow-up, and data collection	5-7
Participants	6	(*a*) Give the eligibility criteria, and the sources and methods of selection of participants	5-7
Variables	7	Clearly define all outcomes, exposures, predictors, potential confounders, and effect modifiers. Give diagnostic criteria, if applicable	5-7
Data sources/ measurement	8*	For each variable of interest, give sources of data and details of methods of assessment (measurement). Describe comparability of assessment methods if there is more than one group	5-7
Bias	9	Describe any efforts to address potential sources of bias	5-7
Study size	10	Explain how the study size was arrived at	7
Quantitative variables	11	Explain how quantitative variables were handled in the analyses. If applicable, describe which groupings were chosen and why	7
Statistical methods	12	(*a*) Describe all statistical methods, including those used to control for confounding	7
		(*b*) Describe any methods used to examine subgroups and interactions	N/A
		(*c*) Explain how missing data were addressed	7
		(*d*) If applicable, describe analytical methods taking account of sampling strategy	7
		(*e*) Describe any sensitivity analyses	Suppl Material
**Results**			
Participants	13*	(a) Report numbers of individuals at each stage of study—eg numbers potentially eligible, examined for eligibility, confirmed eligible, included in the study, completing follow-up, and analysed	8-9
		(b) Give reasons for non-participation at each stage	8-9
		(c) Consider use of a flow diagram	Figure 2
Descriptive data	14*	(a) Give characteristics of study participants (eg demographic, clinical, social) and information on exposures and potential confounders	8, Tab 1, 2

For peer review only - http://bmjopen.bmj.com/site/about/guidelines.xhtml

		(b) Indicate number of participants with missing data for each variable of interest	Suppl.material
Outcome data	15*	Report numbers of outcome events or summary measures	Tab 2, 3, 4, 5
Main results	16	(*a*) Give unadjusted estimates and, if applicable, confounder-adjusted estimates and their precision (eg, 95% confidence interval). Make clear which confounders were adjusted for and why they were included	7-9<br>Tab 2-5
		(*b*) Report category boundaries when continuous variables were categorized	Tab 2-3
		(*c*) If relevant, consider translating estimates of relative risk into absolute risk for a meaningful time period	N/A
Other analyses	17	Report other analyses done—eg analyses of subgroups and interactions, and sensitivity analyses	Suppl. material
**Discussion**			
Key results	18	Summarise key results with reference to study objectives	9
Limitations	19	Discuss limitations of the study, taking into account sources of potential bias or imprecision. Discuss both direction and magnitude of any potential bias	10-11
Interpretation	20	Give a cautious overall interpretation of results considering objectives, limitations, multiplicity of analyses, results from similar studies, and other relevant evidence	10-12
Generalisability	21	Discuss the generalisability (external validity) of the study results	10-12
**Other information**			
Funding	22	Give the source of funding and the role of the funders for the present study and, if applicable, for the original study on which the present article is based	3

*Give information separately for exposed and unexposed groups.

Note: An Explanation and Elaboration article discusses each checklist item and gives methodological background and published examples of transparent reporting. The STROBE checklist is best used in conjunction with this article (freely available on the Web sites of PLoS Medicine at http://www.plosmedicine.org/, Annals of Internal Medicine at http://www.annals.org/, and Epidemiology at http://www.epidem.com/). Information on the STROBE Initiative is available at www.strobe-statement.org.

For peer review only

For peer review only - http://bmjopen.bmj.com/site/about/guidelines.xhtml
